# A genome-wide analysis of the small auxin-up RNA (*SAUR)* gene family in cotton

**DOI:** 10.1186/s12864-017-4224-2

**Published:** 2017-10-23

**Authors:** Xihua Li, Guoyuan Liu, Yanhui Geng, Man Wu, Wenfeng Pei, Honghong Zhai, Xinshan Zang, Xingli Li, Jinfa Zhang, Shuxun Yu, Jiwen Yu

**Affiliations:** 1 State Key Laboratory of Cotton Biology, Institute of Cotton Research of CAAS, Anyang, 455000 China; 2 College of Agronomy, Northwest A&F University, Yangling, 712100 China; 3 Department of Plant and Environmental Sciences, New Mexico State University, Las Cruces, 88003 USA

**Keywords:** *Gossypium* spp., Small auxin-up RNA (*SAUR*), Gene expression patterns, Fiber development

## Abstract

**Background:**

Small auxin-up RNA (*SAUR*) gene family is the largest family of early auxin response genes in higher plants, which have been implicated in the regulation of multiple biological processes. However, no comprehensive analysis of *SAUR* genes has been reported in cotton (*Gossypium* spp.).

**Results:**

In the study, we identified 145, 97, 214, and 176 *SAUR* homologous genes in the sequenced genomes of *G. raimondii*, *G. arboreum*, *G. hirsutum*, and *G. barbadense*, respectively. A phylogenetic analysis revealed that the *SAUR* genes can be classified into 10 groups. A further analysis of chromosomal locations and gene duplications showed that tandem duplication and segmental duplication events contributed to the expansion of the *SAUR* gene family in cotton. An exon-intron organization and motif analysis revealed the conservation of SAUR-specific domains, and the auxin responsive elements existed in most of the upstream sequences. The expression levels of 16 *GhSAUR* genes in response to an exogenous application of IAA were determined by a quantitative RT-PCR analysis. The genome-wide RNA-seq data and qRT-PCR analysis of selected *SAUR* genes in developing fibers revealed their differential expressions. The physical mapping showed that 20 *SAUR* genes were co-localized with fiber length quantitative trait locus (QTL) hotspots. Single nucleotide polymorphisms (SNPs) were detected for 12 of these 20 genes between *G. hirsutum* and *G. barbadense*, but no SNPs were identified between two backcross inbred lines with differing fiber lengths derived from a cross between the two cultivated tetraploids.

**Conclusions:**

This study provides an important piece of genomic information for the *SAUR* genes in cotton and lays a solid foundation for elucidating the functions of *SAUR* genes in auxin signaling pathways to regulate cotton growth.

**Electronic supplementary material:**

The online version of this article (10.1186/s12864-017-4224-2) contains supplementary material, which is available to authorized users.

## Background

Auxin, widely distributed in higher plants, influences nearly all aspects of plant growth and development through regulating cell division, expansion, differentiation, and patterning [1]. Studies in transcripts have revealed that early auxin responsive genes are induced by auxin within minutes [2]. Most of early regulated auxin responsive genes are classified into three families: Aux/IAA, Gretchen Hagen3 (GH3), and small auxin-up RNA (SAUR) [3]. Among these early auxin response genes, *SAURs* are considered to be the most abundant. Apart from transcriptional regulations by auxin, many *SAURs* are also regulated post-transcriptionally as a conserved downstream destabilizing element (DST) in the 3′-untranslated region that confers high mRNA instability [4].

Since the first *SAUR* gene was identified in elongating soybean hypocotyl sections [5], members of this gene family have been identified by genome-wide analyses in diverse plant species, such as *Arabidopsis* [3], rice [6], sorghum [7], tomato [8], potato [8], maize [9], citrus [10], and ramie [11]. In total, more than 674 *SAUR* genes were identified from different species, but only a small portion of them have been functionally characterized. In *Arabidopsis*, the overexpression of AtSAUR19 subfamily proteins (AtSAUR19 to AtSAUR24) with a N-terminal tag resulted in root waving, increased hypocotyl elongation and leaf size, defective apical hook maintenance, and altered tropic responses [12]. A further analysis showed that AtSAUR19 stimulated plasma-membrane (PM) H^+^-ATPase activity to promote cell expansion by inhibiting the PP2C-D phosphatases [13, 14]. Other AtSAUR63 (AtSAUR61 to AtSAUR68 and AtSAUR75) subfamily proteins also led to long hypocotyls, petals and stamen filaments in transgenic *Arabidopsis* [15]. Gene *AtSAUR36* has been reported to have a role in promoting leaf senescence [16]. In rice, the up-regulation of the *OsSAUR39* gene negatively regulated auxin biosynthesis and transport [17]. Three other SAUR proteins, i.e., AtSAUR76, AtSAUR77 and AtSAUR78 affected ethylene receptor signaling and promoted plant growth and development upon auxin responses [18]. Recently, many light-induced and/or repressed *SAUR* genes were reported to mediate differential growth of cotyledons and hypocotyls [19]. These results have shown that SAUR proteins regulate diverse aspects of plant growth and development.

Cotton is one of the most important economic crops for its natural textile fiber in the world, as it produces the cotton fiber, a highly elongated cell derived from the ovule epidermis. Previous reports have shown that auxin plays an important role in fiber development [20, 21]. Specifically, the number of lint fibers was significantly increased with an overexpressed *iaaM* gene during fiber initiation in ovules [22]. However, no *SAUR* genes have been reported in cotton thus far. The genome sequences of the two tetraploid cotton species *Gossypium hirsutum -* AD1 [23, 24] and *G. barbadense* - AD2 [25, 26] and the two closest living extant relatives to their descendants *G. raimondii* - D5 [27, 28] and *G. arboreum* - A2 [29] provide an important genomic resource for a genome-wide analysis of gene families and other related genetic studies [30–32]. In this study, a genome-wide identification of *SAUR* genes in the four species with currently sequenced genomes was performed to characterize the *SAUR* gene family with respect to their structural, genomic and gene expression features. The results obtained in this study will provide new data for further studies on auxin signaling in cotton growth and development.

## Methods

### Gene retrieval and characterization analysis

The genome sequences of *G. raimondii* (JGI_v2.1), *G. arboreum* (BGI_v2.0), *G. hirsutum* acc. TM-1 (NAU-NBI_v1.1) and *G. barbadense* acc. 3-79 (HAU-NBI_v1.0) were downloaded from the CottonGen website [33]. To identify potential SAUR proteins in the four cotton species, all the SAUR amino acid sequences from *Arabidopsis*, rice, sorghum, tomato, and potato, maize, citrus and ramie were used as query in local BLAST (with an E-value cut off of 1e-5) searches individually against the four cotton species genome databases. Next, candidate sequences were inspected with the HMMER software 3.0 with the HMM profiles of auxin-inducible signature domain structure (PF02519) and the pfam database [34] to confirm the presence of the conserved SAUR domain. Then, the ProtParam tool [35] was used to analyze the physicochemical parameters (i.e., length, molecular weight, and isoelectric point) of SAUR proteins [35]. Subcellular localization prediction was conducted using the CELLO v2.5 server [36].

### Phylogenetic analysis

Multiple alignments for all of the available and predicted SAUR full-length protein sequences were performed using ClustalX2 with a manually adjustment where appropriate for the alignment of the SAUR domain. A phylogenetic tree was constructed using the Neighbor Joining (NJ) method of MEGA 6.0 [37] with the pairwise deletion option and a Poisson correction model. For a statistical reliability analysis, bootstrap tests were performed with 1000 replicates.

### Chromosomal locations and gene duplication analysis

The physical chromosome locations of all *SAUR* genes were obtained from the genome sequence databases. Mapchart 2.2 [38] software was used to generate the chromosomal location image. The predicted SAUR proteins were first aligned by ClustalW2 at EMBL-EBI (http://www.ebi.ac.uk/Tools/msa/clustalw2/) prior to a gene duplication analysis. Gene duplication events were defined according to the following conditions: the alignment region covered more than 80% of the longer gene and the identity of the aligned regions was over 80% [30].

### Gene structure and conserved motif analysis

The gene exon/intron structure was analyzed using the Gene Structure Display Server (GSDS) [39] by comparing the cDNAs with their corresponding genomic DNA sequences. The Multiple Em for Motif Elicitation (MEME; version 4.11.2) program was used to analyze the protein sequences of the four cotton species *G. raimondii*, *G. arboreum*, *G. hirsutum*, and *G. barbadense* designated GrSAURs, GaSAURs, GhSAURs, and GbSAURs, respectively. The following parameter settings were used: size distribution, zero or one occurrence per sequence; motifs count, 5; and motif width, between 6 and 37 wide.

### Search for upstream sequence elements

For the promoter analysis, 2000 bp of genomic DNA sequences upstream of the start codon (ATG) of each *GhSAUR* genes were downloaded from their genome sequence. The PLACE database [40] was used to search for cis-acting regulatory elements in the putative promoter regions.

### Gene expression analysis

The expression patterns of genes coding for *GhSAURs* in Upland cotton were analyzed in three developmental stages of two backcross inbred lines (BILs) (NMGA-062 and NMGA-105): 0 days post anthesis (DPA) ovules, 3 DPA ovules, and 10 DPA fibers, and two developmental stages of cultivar Xuzhou 142 and its fuzzless-lintless mutant Xuzhou 142 *fl*: −3 and 0 DPA ovules. The two BILs genotypes were derived from an interspecific backcrossing for two generations between Upland SG747 as the recurrent parent and *G. barbadense* Giza75 followed by selfing, and they had a significant difference in fiber length [41, 42]. All the genotypes were grown in the Experimental Farm, Institute of Cotton Research (ICR), Chinese Academy of Agricultural Sciences (CAAS), Anyang, Henan province, China. Flowers at 0 DPA were tagged and harvested at −3, 0, 3 and 10 DPA to dissect ovules at −3, 0, and 3 DPA and developing fibers at 10 DPA. Three biological replications with 15 flowers per replication were used for each sampling stage. Ovules or fibers were immediately frozen in liquid nitrogen after dissection in the field and stored at −80 °C until use. RNA from each tissue sample was extracted using an RNAprep Pure Plant kit per manufacturer’s instructions (Tiangen, China). RNA quality and quantity were checked using an Agilent 2100 Bioanalyzer, then cDNA libraries were constructed. Fragments of 200-700 nt in size were paired-end sequenced using an Illumina HiSeq 2000 platform in BGI (Shenzhen, China) following the manufacturer’s instructions. After removing adapter sequences and low-quality reads including these with more than 5% unknown nucleotides or with more than 20% nucleotides of sequencing quality ≤10, the clean transcriptome sequencing data were submitted to the NCBI Sequence Read Archive (SRA) with the accession numbers SRP038911 and SRP039385 for NMGA-062 and NMGA-105, and accession number SRP056184 for Xuzhou 142 and Xuzhou 142 *fl*. Fragments per kilobase of transcript per million fragments (FPKM value) were calculated to normalize the expression level of each expressed *GhSAUR* gene based on gene length and the number of mapped reads. The formula is FPKM=$\frac{10^6C}{NL/{10}^3}$, where C is the number of fragments that are uniquely aligned to a specific gene, N is the total number of fragments that are uniquely aligned to all genes, and L is the number of bases on the specific gene. The expression profiles were clustered using the Cluster 3.0 software [43].

### Quantitative RT-PCR analysis

To further characterize the expression of selected *GhSAUR* genes, tissue samples were collected at −3, 0, 3, 5, 7, 10, 15, 20, and 25 DPA ovaries (i.e., bolls) from NMGA-062 only and dissected for ovules at −3, 0 and 3 DPA and fiber at other stages. The tissues were immediately frozen in liquid nitrogen and stored at −80 °C until used for total RNA extraction. Root, stem, and leaf samples were also harvested. For each tissue sample, three biological replications were used.

To study the responses of selected *GhSAURs* to IAA application, seed from cultivar CCRI 10 (*G. hirsutum*) was planted in potting soil at 25 °C in a culture room with a 16-h light/8-h dark cycle. Young plants at the four-true leaf stage were treated with 100 mM IAA, and control plants were sprayed with an equal volume of ddH_2_O. Leaves were then harvested at 0, 5, 10 and 30 min, and 1 h after the treatment for RNA extraction. Three biological replications (10 plants per replication) were used for each time point.

Total RNA was isolated from various tissue samples with a Tiangen RNAprep Pure Plant kit (Tiangen, China) according to the manufacturer’s instructions. The first-strand cDNA fragment was synthesized from total RNA using PrimeScript®RT Reagent kit (Takara, Japan). Then the cDNA templates were diluted 8 fold and used for quantitative RT-PCR (qRT-PCR). Gene specific primers (Additional file 1: Table S1) were designed using the Primer 5.0 software. The histone-3 gene (AF024716) was used as the internal control, as this gene as a reference gene has been commonly used in numerous studies in plants including cotton to verify different gene expression levels in various tissue samples [44, 45]. The qRT-PCR experiment with three replicates was performed on a Mastercycler® ep realplex (Eppendorf, German) in a volume of 20 μL containing 10 μL of 2 × UltraSYBR Mixture (With ROX) (CWBIO, China), 6.2 μL of RNase-Free water, 3 μL of cDNA template, 400 nM of forward and reverse primers into 96-well plates. The thermal cycling conditions were as follows: an initial denaturation step of 10 min at 95 °C, followed by 40 cycles of 15 s at 95 °C for denaturation, 25 s at 60 °C for annealing and 30 s at 72 °C for extension. Then, the melting curve analysis was performed. The relative expression levels of genes were calculated using the 2^-△△CT^ method and normalized to the histone-3 gene. The results were statistically analyzed using a t test.

### Co-localization of *SAUR* genes with quantitative trait loci (QTL) for fiber length (FL) and single nucleotide polymorphism (SNP) identification of FL-QTL co-localized *SAUR* genes

To co-localize *GhSAURs* with QTL for FL, we downloaded molecular markers for FL QTL hotspots reported in interspecific *G. hirsutum* × *G. barbadense* populations [46] and markers for 4 FL QTLs reported in an BIL population containing the two BILs (NMGA-062 and NMGA-105) differing in fiber length [41]. Based on the anchoring marker’s location in the *G. hirsutum* TM-1 genome [24], the positions of the corresponding QTL hotspots for FL in chromosomes and the 4 FL QTL regions identified in the BIL population were determined. Chromosome locations of *SAUR* genes within the FL QTL regions (25 cM) were considered to be the targeted co-localized *SAUR* genes.

To identify SNPs, FL-QTL co-localized *SAUR* genes in allotetraploid *G. hirsutum* genome [24] were compared with homologous/allelic genes from two *G. barbadense* genomes [25, 26] *G. raimondii* genome [27], and *G. arboreum* genome [29] using a local Blastn program. Then, genes from the A2 genome and the At subgenome of AD1 and AD2 were aligned with ClustalX2 using default parameters, as with the genes from the D5 genome and the Dt subgenome of AD1 and AD2. SNPs between allotetraploids and diploids were identified manually for each QTL co-localized *SAUR* gene.

## Results

### Identification of *SAUR* gene family in two diploid and two allotetraploid cotton

With all the SAUR amino acid sequences from *Arabidopsis* (67), rice (56), sorghum (71), tomato (99), potato (134), maize (79), citrus (70) and ramie (71) as query, a Blast search against the CDS data identified a total of 157, 107, 227, and 192 predicted *SAUR* sequences in *G. raimondii*, *G. arboreum*, *G. hirsutum*, and *G. barbadense*, respectively. The putative *SAUR* members were then analyzed for conserved domains using the HMMER and Pfam programs, leading to the identification of 145, 97, 214, and 176 *SAUR* genes in *G. raimondii*, *G. arboreum*, *G. hirsutum*, and *G. barbadense*, respectively (Table 1; Additional files 2, 3 and 4: Table S2, S3, S4). These genes were named consecutively from *GrSAUR1* to *GrSAUR145*, *GaSAUR1* to *GaSAUR97*, *GhSAUR1* to *GhSAUR214*, and *GbSAUR1* to *GbSAUR176* for the four species, respectively, according to the order of their corresponding chromosomal locations. More than 97.5% of the 632 identified *SAUR* genes encode proteins ranging between 64 to 185 amino acids (AA), except for 16 genes with different lengths, i.e., less than 64 or more than 185 AA (i.e., *GrSAUR140* encoding protein with 53 AA, *GbSAUR31*, *GrSAUR109*, *GbSAUR27*, *GbSAUR158*, *GrSAUR40*, *GbSAUR91*, *GhSAUR126*, *GrSAUR134*, *GbSAUR161*, *GaSAUR43*, *GbSAUR13*, *GrSAUR138*, *GbSAUR125*, *GhSAUR114* and *GbSAUR34* encoding proteins with 190, 198, 199, 204, 206, 226, 228, 232, 232, 232, 239, 240, 242, 252, 295 and 376 AA, respectively). The predicted *SAUR* genes encode proteins with the predicted molecular weight (Mw) ranging from 6.06 to 43.11 kDa and the theoretical isoelectric point (pI) varying between 4.69 and 11.42. The protein subcellular localization prediction showed that 397 of the 632 SAUR proteins were located in the nucleus, while others were plasma membrane, cytoplasmic, mitochondrial, chloroplast, or extracellular localized.

**Table 1:** The *SAUR* gene family in *Gossypium raimondii*

Gene ID	Name	Chromosome number	Location	Intron	Length AA	MW/kDa	PI	Predicted subcellular localization
GrSAUR1	Gorai.001G017500.1	Chr1	1,615,768-1,617,374(+)	1	157	17.92	5.97	Nuclear(1.616)/Cytoplasmic(1.523)
GrSAUR2	Gorai.001G017600.1	Chr1	1,629,855-1,630,662(−)	0	124	13.93	5.59	Nuclear(2.037)/Mitochondrial(1.408)
GrSAUR3	Gorai.001G056100.1	Chr1	5,438,812-5,439,758(+)	0	174	19.53	8.9	Nuclear(3.349)
GrSAUR4	Gorai.001G244500.1	Chr1	48,767,330-48,768,039(−)	0	104	11.89	8.91	Mitochondrial(2.554)
GrSAUR5	Gorai.001G244700.1	Chr1	48,849,478-48,849,772(−)	0	71	8.82	5.7	Extracellular(1.110)/Cytoplasmic(1.063)
GrSAUR6	Gorai.001G245000.1	Chr1	48,885,401-48,885,678(−)	1	80	9.17	10.46	Mitochondrial(2.153)
GrSAUR7	Gorai.001G245100.1	Chr1	48,949,120-48,949,404(−)	0	94	10.56	9.25	Mitochondrial(1.764)/PlasmaMembrane(1.598)
GrSAUR8	Gorai.001G245200.1	Chr1	48,953,747-48,953,998(+)	0	83	9.21	8.69	Mitochondrial(2.051)
GrSAUR9	Gorai.001G245300.1	Chr1	48,959,152-48,959,467(−)	0	95	10.70	8.71	Mitochondrial(1.715)/Nuclear(1.086)
GrSAUR10	Gorai.001G245400.1	Chr1	49,022,365-49,022,661(−)	1	86	9.67	8.98	Mitochondrial(1.735)/Nuclear(1.232)
GrSAUR11	Gorai.001G245500.1	Chr1	49,029,187-49,029,450(−)	0	87	9.87	10.29	Mitochondrial(2.957)
GrSAUR12	Gorai.001G245600.1	Chr1	49,036,609-49,036,207(−)	0	98	11.02	9.58	Mitochondrial(2.247)
GrSAUR13	Gorai.001G245700.1	Chr1	49,037,471-49,038,414(+)	0	79	8.97	6.71	Nuclear(1.309)/Mitochondrial(1.055)
GrSAUR14	Gorai.001G245800.1	Chr1	49,039,305-49,040,136(+)	0	96	10.67	7.82	Chloroplast(1.497)/Mitochondrial(1.317)
GrSAUR15	Gorai.001G246200.1	Chr1	49,068,911-49,069,132(−)	0	73	8.29	5.87	Cytoplasmic(1.044)
GrSAUR16	Gorai.001G246300.1	Chr1	49,083,746-49,084,276(−)	0	72	8.17	9.99	Mitochondrial(2.350)
GrSAUR17	Gorai.001G246400.1	Chr1	49,089,129-49,089,840(+)	0	86	9.37	9.65	Mitochondrial(2.239)
GrSAUR18	Gorai.001G246500.1	Chr1	49,093,160-49,094,276(+)	0	96	10.73	9.65	Mitochondrial(2.601)
GrSAUR19	Gorai.001G246600.1	Chr1	49,103,722-49,104,359(+)	0	101	11.52	9.56	Mitochondrial(3.427)
GrSAUR20	Gorai.001G246700.1	Chr1	49,113,411-49,114,457(+)	0	94	10.53	6.26	Mitochondrial(1.852)
GrSAUR21	Gorai.001G246800.1	Chr1	49,115,279-49,116,348(+)	0	94	10.46	6.39	Cytoplasmic(1.765)/Mitochondrial(1.270)
GrSAUR22	Gorai.002G036000.1	Chr2	280,592-2,807,117(+)	0	144	17.05	8.41	Nuclear(2.870)
GrSAUR23	Gorai.002G106000.1	Chr2	14,004,508-14,005,911(+)	0	141	16.09	9.34	Mitochondrial(1.938)
GrSAUR24	Gorai.002G124000.1	Chr2	18,059,123-18,060,363(+)	0	106	12.06	7.81	Mitochondrial(1.608)/Nuclear(1.500)
GrSAUR25	Gorai.002G124300.1	Chr2	18,174,772-18,175,224(−)	0	150	17.25	9.71	Mitochondrial(2.042)
GrSAUR26	Gorai.002G162000.1	Chr2	37,452,379-37,453,133(+)	0	103	11.78	9.74	Mitochondrial(2.171)
GrSAUR27	Gorai.002G202800.1	Chr2	54,437,591-54,438,058(−)	0	155	17.46	8.98	Mitochondrial(1.307)/Nuclear(1.228)
GrSAUR28	Gorai.002G202900.1	Chr2	54,439,265-54,439,708(−)	0	147	16.32	9.71	Mitochondrial(1.306)/PlasmaMembrane(1.221)
GrSAUR29	Gorai.002G203000.1	Chr2	54,441,032-54,441,484(−)	0	150	16.87	9.59	Mitochondrial(1.562)/PlasmaMembrane(1.028)/Nuclear(1.009)
GrSAUR30	Gorai.002G203100.1	Chr2	54,442,712-54,443,320(−)	0	151	17.02	9.27	Mitochondrial(1.582)/PlasmaMembrane(1.248)
GrSAUR31	Gorai.003G168900.1	Chr3	43,938,904-43,939,884(−)	0	160	18.83	10.61	Mitochondrial(2.278)/Nuclear(1.583)
GrSAUR32	Gorai.004G044200.1	Chr4	3,855,770-3,856,615(+)	0	121	14.26	7.3	Nuclear(2.002)
GrSAUR33	Gorai.004G123800.1	Chr4	32,179,516-32,180,140(+)	1	132	15.66	6.85	Mitochondrial(1.572)/Nuclear(1.337)
GrSAUR34	Gorai.004G149700.1	Chr4	42,322,441-42,322,797(−)	0	118	14.00	9.01	Nuclear(1.977)/Mitochondrial(1.434)
GrSAUR35	Gorai.004G163300.1	Chr4	45,433,271-45,433,723(−)	1	132	15.54	9.79	Mitochondrial(1.904)
GrSAUR36	Gorai.004G163400.1	Chr4	45,497,584-45,498,096(−)	0	170	19.63	10.24	Mitochondrial(2.920)
GrSAUR37	Gorai.004G201200.1	Chr4	52,760,164-52,761,207(+)	0	163	18.99	10.17	Mitochondrial(2.278)
GrSAUR38	Gorai.004G269500.1	Chr4	60,455,793-60,456,903(−)	0	142	15.91	7.82	Mitochondrial(1.415)
GrSAUR39	Gorai.004G285600.1	Chr4	61,641,514-61,641,950(+)	1	101	11.42	8.95	Nuclear(1.681)/Mitochondrial(1.473)/Cytoplasmic(1.115)
GrSAUR40	Gorai.005G249200.1	Chr5	62,880,875-62,882,624(+)	1	206	23.97	10.25	Mitochondrial(1.433)/Nuclear(1.317)
GrSAUR41	Gorai.005G249400.1	Chr5	62,904,501-62,905,022(−)	0	79	8.92	7.88	Mitochondrial(1.395)
GrSAUR42	Gorai.005G249500.1	Chr5	62,906,555-62,906,794(−)	0	79	8.90	8.93	Mitochondrial(1.189)/Nuclear(1.011)
GrSAUR43	Gorai.005G249700.1	Chr5	62,909,228-62,910,023(−)	0	79	8.95	7.89	Mitochondrial(1.276)/Nuclear(1.242)
GrSAUR44	Gorai.005G249800.1	Chr5	62,911,828-62,912,064(−)	0	78	8.85	7.89	Mitochondrial(1.438)/Nuclear(1.297)
GrSAUR45	Gorai.005G249900.1	Chr5	62,916,783-62,917,022(−)	0	79	8.91	6.71	Mitochondrial(1.261)/Nuclear(1.177)
GrSAUR46	Gorai.005G250100.1	Chr5	62,926,267-62,927,447(+)	0	79	8.94	9.21	Mitochondrial(1.812)
GrSAUR47	Gorai.005G250200.1	Chr5	62,934,108-62,934,684(+)	0	79	8.83	6.53	Extracellular(1.406)/Nuclear(1.177)/Mitochondrial(1.039)
GrSAUR48	Gorai.005G250300.1	Chr5	62,935,399-62,936,252(+)	0	113	12.99	9.03	PlasmaMembrane(3.361)
GrSAUR49	Gorai.005G250400.1	Chr5	62,937,212-62,938,166(+)	0	78	8.81	7.89	Nuclear(1.690)/Mitochondrial(1.319)
GrSAUR50	Gorai.005G250500.1	Chr5	62,939,729-62,940,314(+)	0	79	8.91	7.89	Mitochondrial(1.545)/Nuclear(1.283)
GrSAUR51	Gorai.005G250600.1	Chr5	62,941,478-62,941,910(+)	0	79	8.81	6.71	Mitochondrial(1.311)/Nuclear(1.032)
GrSAUR52	Gorai.005G250700.1	Chr5	62,949,815-62,950,331(+)	0	79	8.92	8.93	Mitochondrial(1.237)
GrSAUR53	Gorai.005G250800.1	Chr5	62,951,581-62,952,293(−)	0	92	10.33	6.55	Mitochondrial(1.594)/PlasmaMembrane(1.012)
GrSAUR54	Gorai.005G250900.1	Chr5	62,960,626-62,960,916(−)	0	96	10.68	9.3	Mitochondrial(2.249)
GrSAUR55	Gorai.005G251000.1	Chr5	62,969,862-62,969,303(+)	0	96	10.64	8.66	Mitochondrial(1.469)/Nuclear(1.215)/Extracellular(1.154)
GrSAUR56	Gorai.005G251100.1	Chr5	62,985,151-62,985,441(+)	0	96	10.76	8.98	Mitochondrial(2.622)
GrSAUR57	Gorai.005G251200.1	Chr5	62,989,841-62,990,113(−)	0	90	10.32	7.81	Nuclear(1.458)
GrSAUR58	Gorai.005G251400.1	Chr5	63,001,840-63,002,157(−)	0	105	12.09	7.88	Nuclear(1.720)/Mitochondrial(1.716)
GrSAUR59	Gorai.005G251500.1	Chr5	63,005,563-63,006,419(+)	0	105	12.11	9.21	Mitochondrial(2.949)
GrSAUR60	Gorai.005G251600.1	Chr5	63,014,837-63,015,348(+)	0	94	10.57	7.76	Mitochondrial(1.577)/Extracellular(1.068)
GrSAUR61	Gorai.005G251700.1	Chr5	63,016,684-63,017,267(+)	0	104	11.72	9.03	Mitochondrial(2.483)
GrSAUR62	Gorai.005G256400.1	Chr5	63,318,324-63,318,851(+)	0	138	16.05	9.52	Mitochondrial(1.593)/Nuclear(1.232)
GrSAUR63	Gorai.005G256500.1	Chr5	63,320,204-63,320,849(+)	0	147	16.52	9.01	Nuclear(1.268)
GrSAUR64	Gorai.005G256600.1	Chr5	63,345,303-63,346,064(−)	0	147	16.61	9.42	Nuclear(1.484)
GrSAUR65	Gorai.005G256700.1	Chr5	63,347,114-63,348,038(−)	0	147	16.64	9.12	Nuclear(1.348)
GrSAUR66	Gorai.005G256800.1	Chr5	63,349,316-63,349,771(−)	0	151	17.25	9.28	PlasmaMembrane(2.087)
GrSAUR67	Gorai.005G256900.1	Chr5	63,351,050-63,351,454(−)	0	134	15.32	9.37	PlasmaMembrane(1.359)/Nuclear(1.193)/Mitochondrial(1.169)
GrSAUR68	Gorai.005G257000.1	Chr5	63,352,122-63,352,535(−)	0	137	15.89	8.89	Nuclear(1.742)
GrSAUR69	Gorai.005G257100.1	Chr5	63,354,207-63,354,620(−)	0	137	15.99	9.52	Mitochondrial(1.658)/Nuclear(1.285)
GrSAUR70	Gorai.005G257200.1	Chr5	63,356,605-63,357,048(−)	0	147	16.47	9.32	Nuclear(1.401)/Chloroplast(1.342)/Mitochondrial(1.080)
GrSAUR71	Gorai.006G007200.1	Chr6	1,498,825-1,499,363(+)	0	106	12.63	8.6	Nuclear(2.192)
GrSAUR73	Gorai.006G154300.1	Chr6	41,306,397-41,307,777(−)	2	127	14.51	9.21	Nuclear(1.623)/Extracellular(1.064)
GrSAUR72	Gorai.006G154300.2	Chr6	41,306,397-41,307,777(−)	0	127	14.42	5.91	Cytoplasmic(1.635)
GrSAUR74	Gorai.006G163400.1	Chr6	42,370,463-42,371,381(+)	0	133	15.08	5.07	Mitochondrial(1.441)/Nuclear(1.432)/Cytoplasmic(1.128)
GrSAUR75	Gorai.006G163500.1	Chr6	42,377,888-42,378,232(+)	1	81	9.65	9.88	Mitochondrial(1.532)/Cytoplasmic(1.287)/Nuclear(1.193)
GrSAUR76	Gorai.006G163600.1	Chr6	42,404,212-42,405,041(+)	0	133	15.05	5.07	Mitochondrial(1.543)/Nuclear(1.368)
GrSAUR77	Gorai.006G163800.1	Chr6	42,412,170-42,412,587(+)	1	126	14.30	8.49	Nuclear(1.932)/Mitochondrial(1.702)
GrSAUR78	Gorai.006G163900.1	Chr6	42,419,577-42,420,926(+)	0	112	12.88	4.85	Cytoplasmic(1.491)/Nuclear(1.050)
GrSAUR79	Gorai.006G164000.1	Chr6	42,423,009-42,423,594(+)	0	123	14.07	7.83	Nuclear(2.049)/Mitochondrial(1.486)
GrSAUR80	Gorai.006G164100.1	Chr6	42,425,630-42,426,271(+)	0	126	14.55	7.83	Mitochondrial(1.905)/Nuclear(1.315)
GrSAUR81	Gorai.006G165600.1	Chr6	42,539,210-42,539,581(+)	0	123	14.15	9.08	Extracellular(2.919)
GrSAUR82	Gorai.007G089900.1	Chr7	6,550,230-6,550,733(+)	0	167	18.97	10.1	Mitochondrial(2.525)
GrSAUR83	Gorai.007G195900.1	Chr7	19,409,462-19,410,253(+)	0	105	12.00	8.62	Mitochondrial(1.684)/Cytoplasmic(1.511)
GrSAUR84	Gorai.008G032500.1	Chr8	3,910,820-3,911,852(+)	0	162	18.28	9.71	Mitochondrial(1.971)/Nuclear(1.789)
GrSAUR85	Gorai.008G032600.1	Chr8	3,952,535-3,953,523(−)	0	104	11.88	8.51	Mitochondrial(2.231)
GrSAUR86	Gorai.008G113100.1	Chr8	34,515,914-34,516,922(+)	0	164	18.72	10.64	Mitochondrial(2.851)
GrSAUR87	Gorai.008G128100.1	Chr8	36,977,552-36,978,549(+)	0	122	14.34	8.5	Nuclear(2.376)
GrSAUR88	Gorai.008G157200.1	Chr8	41,984,144-41,985,508(+)	0	154	17.45	9.34	Nuclear(2.361)
GrSAUR89	Gorai.008G157200.2	Chr8	41,984,144-41,985,508(+)	0	154	17.45	9.34	Nuclear(2.361)
GrSAUR90	Gorai.008G208300.1	Chr8	49,321,783-49,322,349(−)	0	139	15.64	9.32	Nuclear(3.111)
GrSAUR91	Gorai.008G236200.1	Chr8	52,182,178-52,183,143(−)	0	164	19.19	10.46	Mitochondrial(2.156)/Nuclear(1.528)
GrSAUR92	Gorai.008G265900.1	Chr8	54,517,459-54,518,713(−)	0	122	14.36	6.87	Nuclear(2.160)
GrSAUR93	Gorai.009G037700.1	Chr9	2,776,385-2,777,283(+)	0	163	18.43	7.73	Nuclear(1.758)/Mitochondrial(1.092)
GrSAUR94	Gorai.009G037800.1	Chr9	2,778,553-2,779,157(−)	0	123	13.74	5.35	Nuclear(1.624)/Mitochondrial(1.303)
GrSAUR95	Gorai.009G144300.1	Chr9	10,942,378-10,943,104(+)	0	140	15.57	5.02	Nuclear(1.689)/Mitochondrial(1.031)
GrSAUR96	Gorai.009G189200.1	Chr9	14,553,243-14,553,545(+)	0	100	11.45	10.38	Mitochondrial(2.168)
GrSAUR97	Gorai.009G195800.1	Chr9	15,048,472-15,050,245(+)	0	105	12.04	6.83	Mitochondrial(1.586)/Nuclear(1.551)
GrSAUR98	Gorai.009G195800.2	Chr9	15,048,472-15,050,245(+)	0	105	12.04	6.83	Mitochondrial(1.586)/Nuclear(1.551)
GrSAUR99	Gorai.009G196000.1	Chr9	15,088,996-15,089,460(−)	0	154	17.69	10.04	Mitochondrial(2.329)
GrSAUR100	Gorai.009G270400.1	Chr9	22,574,039-22,575,114(+)	0	104	11.90	9.06	Mitochondrial(2.106)/Nuclear(1.457)
GrSAUR101	Gorai.009G270500.1	Chr9	22,595,674-22,596,117(−)	0	147	17.02	9.57	Mitochondrial(1.673)/Nuclear(1.267)
GrSAUR102	Gorai.009G289300.1	Chr9	24,863,485-24,863,904(+)	0	82	9.59	9.3	Nuclear(1.735)/Mitochondrial(1.401)
GrSAUR103	Gorai.009G352900.1	Chr9	45,477,526-45,477,954(−)	0	142	16.29	9.12	Mitochondrial(1.798)/Nuclear(1.133)
GrSAUR104	Gorai.009G400900.1	Chr9	57,808,384-57,808,761(−)	0	125	13.88	6.73	Nuclear(1.308)/Cytoplasmic(1.148)/Mitochondrial(1.145)
GrSAUR105	Gorai.009G409300.1	Chr9	61,459,699-61,458,422(+)	0	114	12.98	6.89	Chloroplast(1.175)/Mitochondrial(1.173)
GrSAUR106	Gorai.009G416100.1	Chr9	64,103,565-64,103,918(+)	0	117	13.55	8.46	Extracellular(2.548)
GrSAUR107	Gorai.010G005300.1	Chr10	238,684-239,735(+)	0	110	12.62	8.62	Nuclear(2.021)/Mitochondrial(1.406)
GrSAUR108	Gorai.010G005500.1	Chr10	275,408-275,866(−)	0	152	17.10	9.86	Mitochondrial(2.642)
GrSAUR109	Gorai.010G091700.1	Chr10	14,495,966-14,498,408(+)	3	198	22.32	5.29	Nuclear(1.433)/Cytoplasmic(1.237)
GrSAUR110	Gorai.010G091700.2	Chr10	14,496,288-14,498,454(+)	1	161	18.16	5.16	Nuclear(1.969)/Cytoplasmic(1.322)
GrSAUR114	Gorai.010G091700.3	Chr10	14,496,388-14,498,454(+)	1	161	18.16	5.16	Nuclear(1.969)/Cytoplasmic(1.322)
GrSAUR111	Gorai.010G091700.4	Chr10	14,496,345-14,498,408(+)	1	161	18.16	5.16	Nuclear(1.969)/Cytoplasmic(1.322)
GrSAUR113	Gorai.010G091700.5	Chr10	14,496,367-14,498,408(+)	1	161	18.16	5.16	Nuclear(1.969)/Cytoplasmic(1.322)
GrSAUR112	Gorai.010G091700.6	Chr10	14,496,367-14,498,408(+)	1	161	18.16	5.16	Nuclear(1.969)/Cytoplasmic(1.322)
GrSAUR115	Gorai.010G091700.7	Chr10	14,496,598-14,498,408(+)	1	161	18.16	5.16	Nuclear(1.969)/Cytoplasmic(1.322)
GrSAUR116	Gorai.010G091700.8	Chr10	14,496,730-14,498,408(+)	1	161	18.16	5.16	Nuclear(1.969)/Cytoplasmic(1.322)
GrSAUR117	Gorai.010G091700.9	Chr10	14,497,040-14,498,408(+)	1	161	18.16	5.16	Nuclear(1.969)/Cytoplasmic(1.322)
GrSAUR118	Gorai.010G092200.1	Chr10	14,625,769-14,626,573(−)	0	126	14.15	5.32	Mitochondrial(1.386)/Nuclear(1.328)
GrSAUR119	Gorai.010G141400.1	Chr10	34,863,658-34,864,380(+)	0	159	18.02	9.32	Nuclear(2.768)
GrSAUR120	Gorai.010G170300.1	Chr10	49,488,730-49,489,183(+)	0	95	11.19	8.66	Mitochondrial(1.707)/Nuclear(1.373)
GrSAUR121	Gorai.010G235800.1	Chr10	60,631,341-60,631,637(+)	0	98	11.76	9.73	Cytoplasmic(2.066)/Mitochondrial(1.394)
GrSAUR122	Gorai.011G002200.1	Chr11	193,299-193,996(−)	0	176	19.75	9.01	Nuclear(2.687)
GrSAUR123	Gorai.011G052800.1	Chr11	4,142,829-4,143,791(+)	0	119	13.88	10.07	Nuclear(2.189)/Mitochondrial(1.517)
GrSAUR124	Gorai.011G052800.2	Chr11	4,142,866-4,143,738(+)	0	119	13.88	10.07	Nuclear(2.189)/Mitochondrial(1.517)
GrSAUR125	Gorai.011G056900.1	Chr11	4,500,795-4,501,702(+)	0	119	13.43	7.73	Mitochondrial(1.538)/Nuclear(1.274)/Chloroplast(1.092)
GrSAUR126	Gorai.011G099000.1	Chr11	10,972,095-10,973,058(+)	0	104	11.83	9.23	Cytoplasmic(1.774)/Mitochondrial(1.435)
GrSAUR127	Gorai.012G037000.1	Chr12	4,571,265-4,571,576(−)	0	103	12.24	6.62	Nuclear(1.767)/Cytoplasmic(1.069)
GrSAUR128	Gorai.012G153700.1	Chr12	32,746,689-32,747,015(+)	0	108	12.54	9.43	Mitochondrial(2.120)
GrSAUR129	Gorai.012G153800.1	Chr12	32,752,110-32,752,454(+)	0	114	13.39	9.57	Mitochondrial(2.160)
GrSAUR130	Gorai.013G141300.1	Chr13	38,291,563-38,293,037(+)	0	143	16.31	8.9	Nuclear(1.590)/Mitochondrial(1.264)
GrSAUR131	Gorai.013G141300.2	Chr13	38,291,616-38,293,037(+)	0	143	16.31	8.9	Nuclear(1.590)/Mitochondrial(1.264)
GrSAUR132	Gorai.013G222500.1	Chr13	54,241,671-54,242,678(+)	0	124	13.89	4.78	Nuclear(1.349)/Mitochondrial(1.105)/Chloroplast(1.084)
GrSAUR133	Gorai.013G268800.1	Chr13	58,076,512-58,076,950(−)	0	140	16.09	6.59	Cytoplasmic(1.835)/Nuclear(1.509)
GrSAUR134	Gorai.N011800.1	scaffold_36	3329-4346(+)	0	232	26.12	9.26	PlasmaMembrane(2.573)
GrSAUR135	Gorai.N011900.1	scaffold_36	5114-5805(−)	0	140	16.18	9.57	Mitochondrial(1.418)/PlasmaMembrane(1.023)/Nuclear(1.022)
GrSAUR136	Gorai.N012000.1	scaffold_36	7594-8037(−)	0	147	16.52	9.06	PlasmaMembrane(1.131)/Nuclear(1.029)
GrSAUR137	Gorai.N012100.1	scaffold_36	9050-9965(−)	0	147	16.73	9.19	Nuclear(2.010)
GrSAUR138	Gorai.N012200.1	scaffold_36	13,475-13,932(−)	1	240	27.46	8.63	PlasmaMembrane(3.312)
GrSAUR139	Gorai.N012300.1	scaffold_36	11,601-14,876(−)	0	99	11.53	10	Mitochondrial(1.666)/PlasmaMembrane(1.257)
GrSAUR140	Gorai.N014500.1	scaffold_108	3-164(+)	0	53	6.06	9.69	Mitochondrial(1.772)
GrSAUR141	Gorai.N014600.1	scaffold_108	1558-2077(+)	0	79	8.94	9.3	Mitochondrial(1.680)
GrSAUR142	Gorai.N014700.1	scaffold_108	2935-3846(+)	0	81	9.15	6.55	Nuclear(1.375)/Mitochondrial(1.293)
GrSAUR143	Gorai.N014800.1	scaffold_108	5215-12,132(+)	1	79	8.91	7.89	Mitochondrial(1.707)/Nuclear(1.032)
GrSAUR144	Gorai.N014900.1	scaffold_108	6574-7195(+)	0	79	8.90	8.89	Mitochondrial(1.519)
GrSAUR145	Gorai.N015000.1	scaffold_108	9410-9987(+)	0	78	8.81	6.71	Nuclear(1.433)/Extracellular(1.053)/Mitochondrial(1.017)

### Phylogenetic analysis of the *SAUR* gene family

**Fig. 1:**
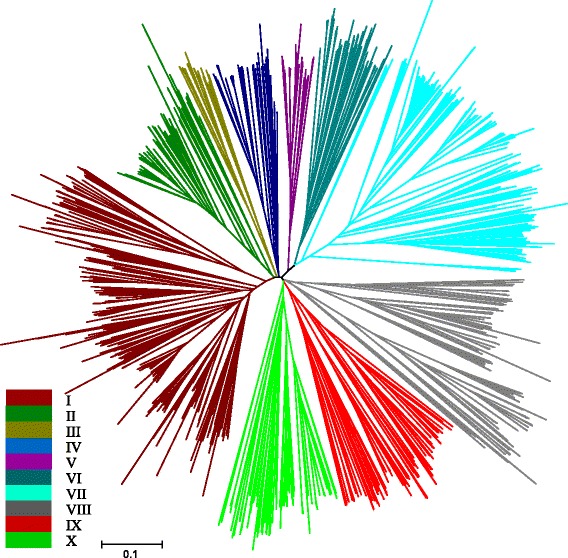
A Phylogenetic tree of SAUR proteins from *Gossypium raimondii*, *G. arboreum*, *G. hirsutum*, *G. barbadense*, *Arabidopsis*, rice, maize, tomato, potato, sorghum, citrus, and ramie. The phylogenetic tree was generated using MEGA 6.0 with the Neighbour-Joining (NJ) method with 1000 bootstrap replicates. Different colored line marks groups I - X of the SAURs

To study the phylogenetic relationship of the SAUR family, we performed a phylogenetic analysis of 647 SAUR protein sequences from *Arabidopsis*, rice, maize, tomato, potato, sorghum, citrus, and ramie and 632 cotton SAURs by generating a phylogenetic tree. As shown in Fig. 1, the SAUR proteins can be placed into 10 distinct groups, designated from group I to X, which were based on their sequence similarities with orthologs in other plants. Group I and VII contained 365 and 237 SAUR members, respectively, and constituted the two largest groups in the phylogeny, while group III and V contained only 24 and 30 members, respectively. Each group contained SAURs from at least 9 plant species. Interestingly, group VI only contained SAURs from dicot species, i.e., *G. raimondii*, *G. arboreum*, *G. hirsutum*, *G. barbadense*, *Arabidopsis*, tomato, potato, citrus, and ramie as seen in Additional file 5: Table S5.

### Chromosomal distribution and duplication events of *SAUR* genes in cotton

**Fig. 2:**
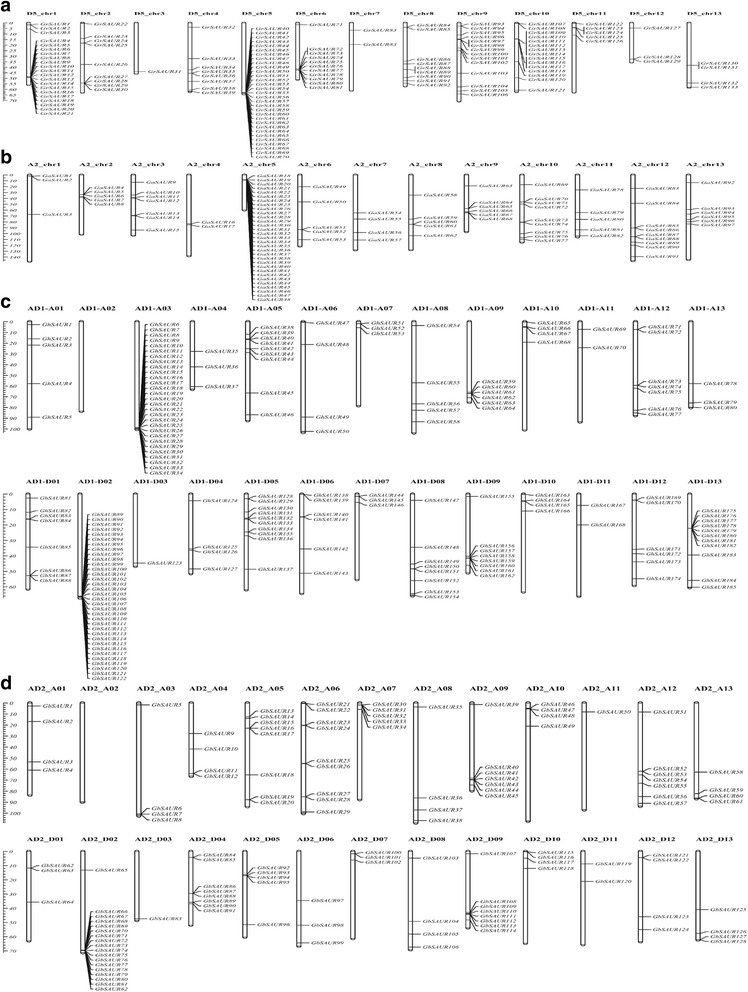
Distribution of *SAUR* genes on *Gossypium raimondii* (**a**), *G. arboreum* (**b**), *G. hirsutum* (**c**), and *G. barbadense* (**d**) chromosomes. The scale represents megabases (Mb). The chromosome numbers of *G. raimondii* (D5_chr1 - D5_chr13), *G. arboreum* (A2_chr1 - A2_chr13), *G. hirsutum* (AD1_A01 - AD1_A13, AD1_D01 - AD1_D13), and *G. barbadense* (AD2_A01 - AD2_A13, AD2_D01 - AD2_D13) are indicated above each vertical bar

Using the genome sequences of the four cotton species as references, the identified 632 *SAUR* genes were mapped onto chromosomes or scaffolds. Of which, 543 of the 632 *SAURs* were assigned to chromosomes, while the remaining 89 *SAURs* were located in unmapped scaffolds (Fig. 2). Of the 133 *GrSAUR*s mapped to 13 chromosomes of *G. raimondii*, chromosome D5_chr5 (with 31 genes) and chromosome D5_chr1 (with 21 genes) had the most number of *SAUR* genes, while D5_chr3 had only 1 *SAUR* gene. The 97 predicted *SAUR* genes in *G. arboreum* were also distributed unevenly across its 13 chromosomes, with chromosome A2_chr5 (similar to the homoeologous chromosome D5_chr5 in *G. raimondii*) harboring more genes (31) and chromosome A2_chr4 harboring the least genes (2). A total of 185 *GhSAUR* genes were mapped to 25 chromosomes of the *G. hirsutum* genome with 1 to 34 genes per chromosome, except for no *SAUR* genes identified on chromosome AD1_A02 in the At subgenome. Similarly, more *SAUR* genes were clustered on chromosomes AD1_A03, AD1_D02, AD1_D05, and AD1_D13. A total of 128 *GbSAUR* genes distributed unevenly over all the *G. barbadense* genome except for chromosome AD2_A02. The number of genes per chromosome ranged from 1 (chromosome AD2_A11) to 18 (chromosome AD2_D02).

**Fig. 3:**
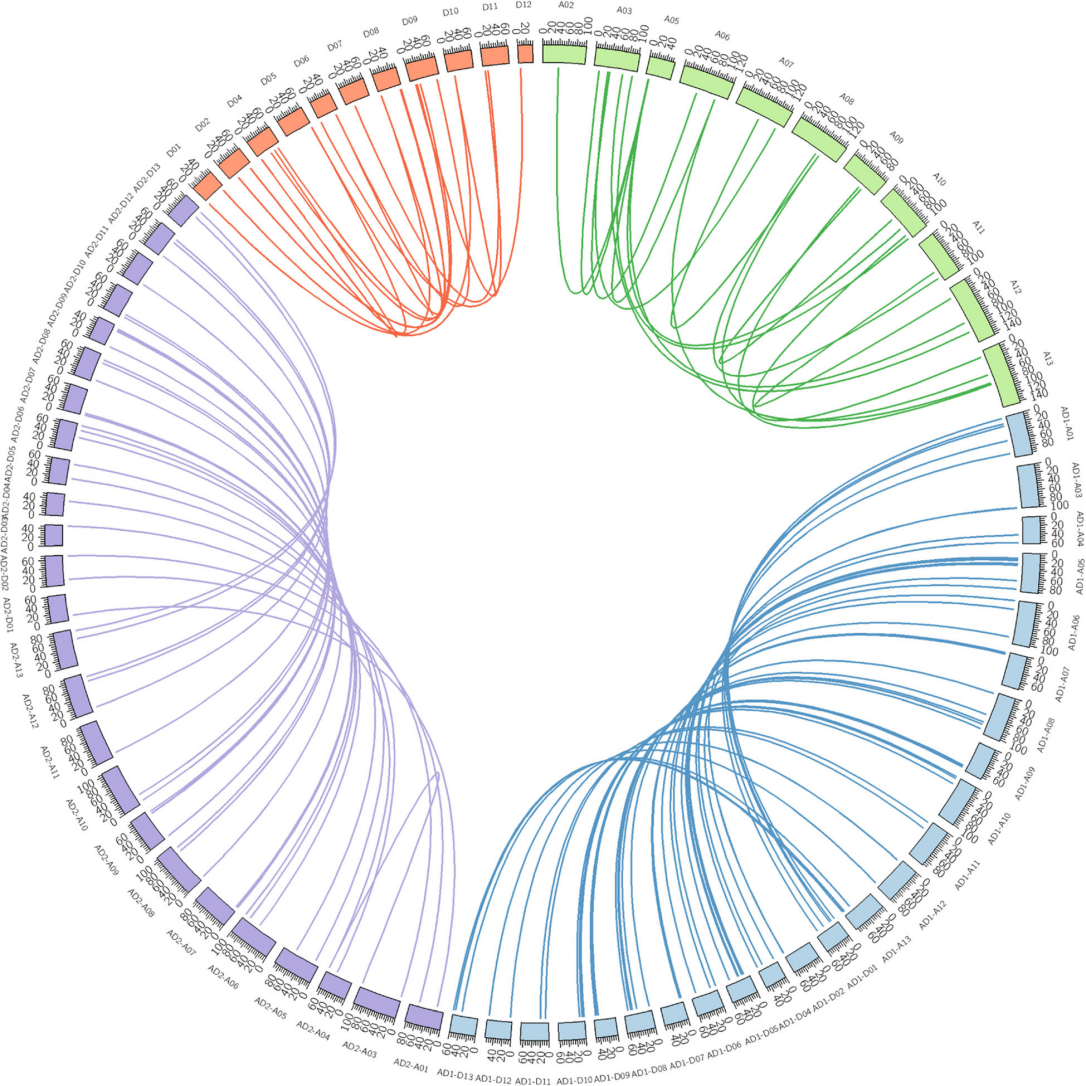
The Circos diagram of paralogous gene pairs identified in *GrSAURs*, *GaSAURs*, *GhSAURs*, and *GbSAURs*. The chromosomes of *Gossypium raimondii*, *G. arboreum*, *G. hirsutum*, and *G. barbadense* were filled with red, green, blue, and purple colors, respectively. A line between two genes indicates a paralog

To elucidate the expanded mechanism of the *SAUR* gene family, we performed a gene duplication event analysis including tandem duplication and segmental duplication in the four cotton species. After multiple and pairwise alignments of *GrSAURs*, *GaASURs*, *GhSAURs* and *GbSAURs*, we chose the paralogous genes with the criteria described in previous studies [30]. As a result, 98, 54, 187 and 144 putative paralogous *SAUR* genes with high gene identity and similarity were found, accounting for 67.6%, 55.7%, 87.4% and 81.8% of the entire *SAUR* gene family in *G. raimondii*, *G. arboreum*, *G. hirsutum*, and *G. barbadense*, respectively. We observed that tandem duplication and segmental duplication events contributed to the expansion of the *SAUR* gene family in cotton. The details for the duplicated gene pairs were listed in Additional file 6: Table S6. In *G. raimondii*, 14 pairs of tandem duplication and 21 pairs of segmental duplication events were detected. The clusters of tandem duplication were mainly on chromosome D2_chr5. In *G. arboreum*, 3 clusters of *SAUR* genes were produced by tandem duplications, and the clusters of genes were distributed on the same chromosome (i.e., A2_chr5). In the same species, 17 pairs of *SAUR* genes were produced by segmental duplications and the two genes in each pair were from different chromosomes. In *G. hirsutum*, 9 clusters of genes with tandem duplications were detected mainly on chromosome AD1_A03 and AD1_D02, and 75 pairs of genes with segmental duplication events were detected with the two genes from each pair distributed on different chromosomes. The most majority of *GbSAUR* genes in *G. barbadense* were found to occur from tandem duplication or segmental duplication events. There are 23 pairs of tandem duplication clusters existed mainly on chromosome AD2_A05 and AD2_D02. 23 gene pairs with segmental duplication all distributed on different chromosomes. Interestingly, most of the tandem duplication events in *GhSAURs* occurred on chromosome AD1_D02. It is apparent that homeologous chromosome 5 in *G. raimondii* (D5) and *G. arboreum* (A2), and chromosome D02 in *G. hirsutum* (AD1) and *G. barbadense* (AD2) all harbored more *SAUR* genes derived from tandem duplications. Chromosome D02 from AD1 and AD2 may be also homeologous to A2_chr5. The tandem duplication gene pairs were shown in Fig. 3 except for genes that were not localized to a specific chromosome.

### Gene structure and conserved motifs

**Fig. 4:**
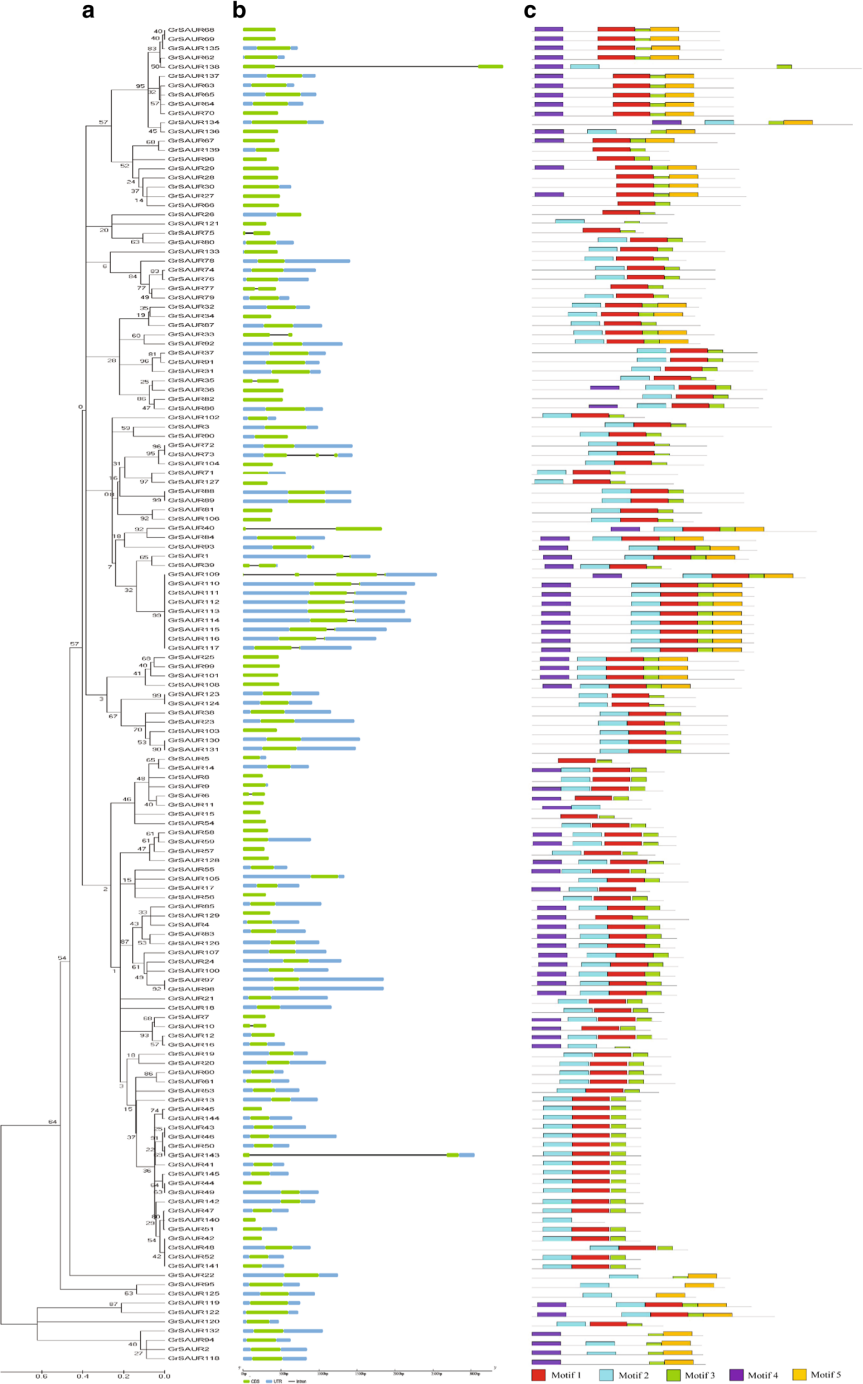
Phylogenetic relationships, gene structure and motif compositions of the *Gossypium raimondii SAUR* genes. **a** The phylogenetic tree was constructed using MEGA 6.0 with the Neighbour-Joining (NJ) method with 1000 bootstrap replicates. **b** Exon/intron structures of *SAUR* genes from *G. raimondii*. The introns, CDS and UTRs are represented by black lines, green and blue boxes respectively. The scale bar represents 0.5 kb. **c** Protein motif. Each motif is represented in the colored box

To gain an insight into the diversification of the *SAUR* genes in cotton, the exon/intron organization and conserved motifs were further analyzed. Based on the evolutionary relationships (Fig. 4a, Additional files 7, 8 and 9: Figure S1A, S2A, S3A), the detailed structure features of *SAUR* genes were shown in Fig. 4b and Additional files 7, 8 and 9: Figure S1B, S2B, S3B. In general, more than 90.7% of *SAUR* genes lacked introns, 19 genes in *GrSAURs*, 4 genes in *GaSAURs*, 11 genes in *GhSAURs*, and 19 genes in *GbSAURs* each had 1 intron. Only 4 genes, namely *GrSAUR73*, *GaSAUR76*, *GhSAUR114*, and *GbSAUR34* each had 2 introns and another 2 genes (*GrSAUR109* and *GbSAUR13*) each had 3 introns. Conserved motifs in the 632 SAUR proteins were identified using the MEME online tool (Fig. 4c, Additional files 7, 8 and 9: Figure S1C, S2C, S3C). Motifs 1, 2, and 3 constitute the conserved SAUR-specific domain of approximately 60 residues in the central region of the sequences and were identified in most of the predicted SAUR proteins. Motifs 4 and 5 accounted for 39.7% and 25.8% of the SAUR members, respectively, suggesting that these features might have contributed to some specific functions in the *SAUR* family.

Similar to the histidine-rich (H-rich) regions found in *Arabidopsis*, sorghum, tomato, and potato [3, 7, 8], the H-rich regions were identified in the sequences of 39 predicted cotton *SAUR* genes. They are 7 *GrSAURs*, 6 *GaSAURs*, 14 *GhSAURs*, and 12 *GbSAURs*. The multiple alignments among these 39 SAUR proteins were shown in Additional file 10: Figure S4, and the H-rich regions were located on both or either of the N-terminal and C-terminal sequences.

### Promoter regions of *GhSAUR* genes

The scanning of cis-acting regulatory DNA elements within promoter regions (2.0 kb from the start codon) of 165 randomly chosen *GhSAUR* genes was performed using the PLACE database. The results revealed that the promoters of the *SAUR* gene family contain numerous DNA elements predicted to be auxin signaling transduction related cis-elements. At least one of the seven major auxin-responsive cis-elements – S000024, S000026, S000234, S000270, S000273, S000360, and S000370, has been found in the promoter regions of the *SAURs*, except for 12 predicted *GhSAURs* genes. Another two regulatory sequences, i.e., Ca^2+^-responsive cis-element (S000501) and calmodulin-binding/CGCG box (S000507) were found in 47 predicted *GhSAURs* genes (Additional file 11: Table S7).

### Responses of *SAUR* genes in leaves to an exogenous IAA application

**Fig. 5:**
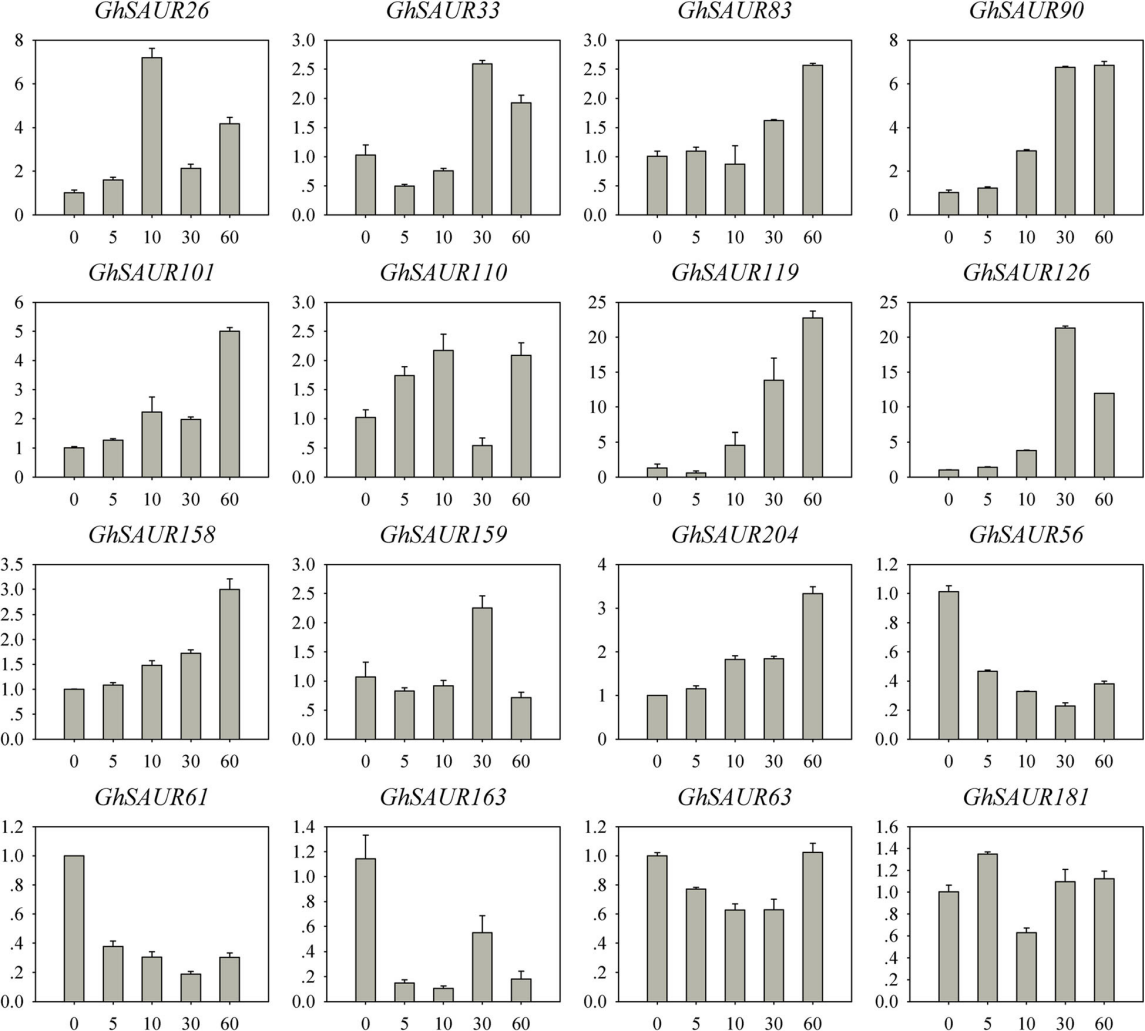
Expression patterns of *GhSAUR* genes in leaves under an IAA treatment. The x-axis represents different minutes (0, 5, 10, 30, and 60) after IAA treatment, and the y-axis indicates the relative expression levels. Error bars show the standard deviation of three biological replicates

The expression of *SAUR* genes is regulated at multiple levels in other reported species [3]. We analyzed the expression of 16 *GhSAUR* genes in leaves under exogenous IAA treatment (Fig. 5). 11 of these genes were up-regulated at 5 min to 1 h after the IAA treatment, while 3 genes (*GhSAUR56*, *GhSAUR61*, and *GhSAUR163*) were down-regulated by the exogenous IAA application. Another 2 genes (*GhSAUR63* and *GhSAUR181*) showed a relatively stable expression regardless of IAA treatment. Therefore, the response of *SAUR* genes to IAA treatment in leaves varies, depending on *SAUR* genes.

### Expression characterization of *GhSAUR* genes in developing ovules and fibers

**Fig. 6:**
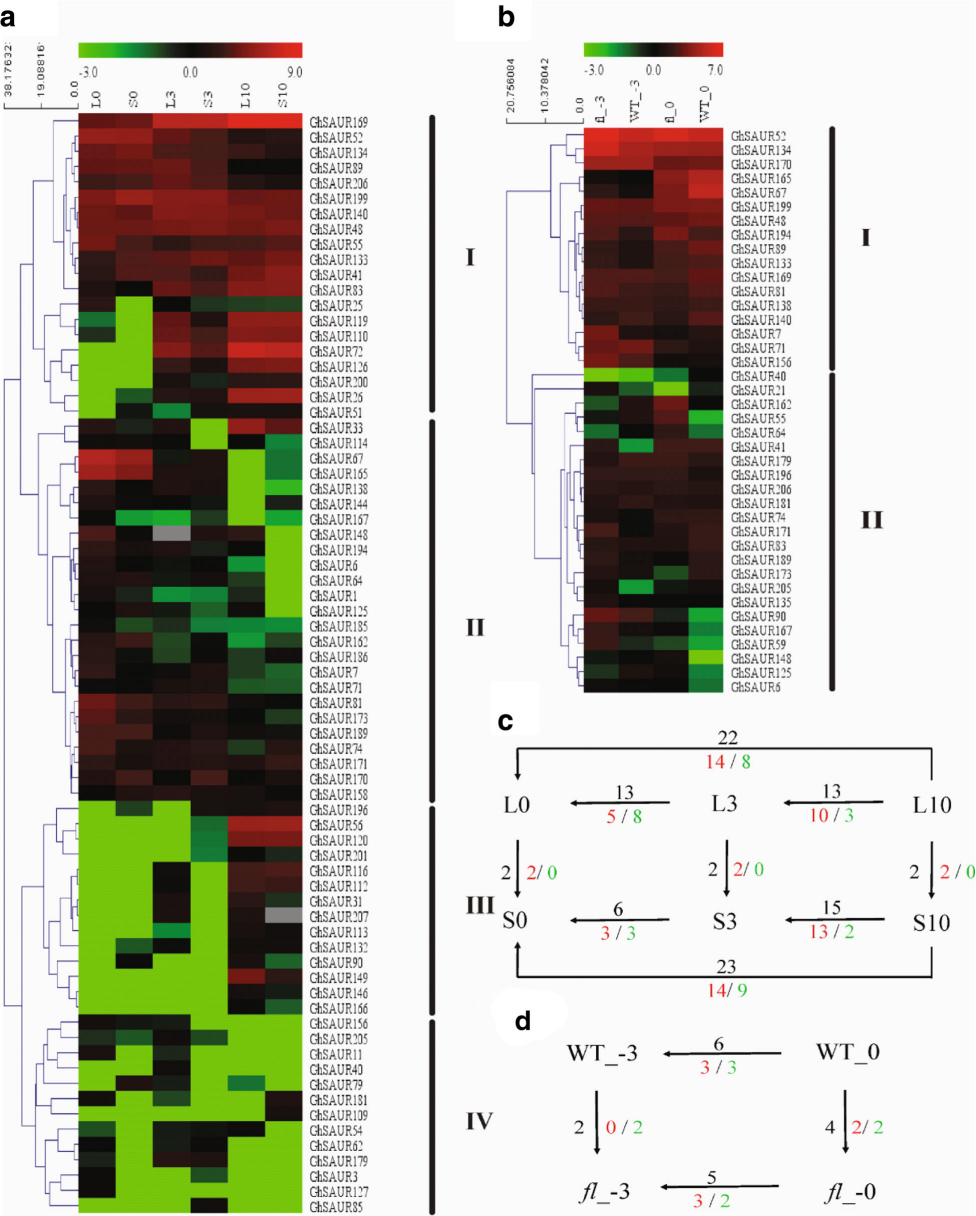
Expression profiles of *GhSAUR* genes based on RNA-seq data of two backcross inbred lines (BILs) and Xuzhou142 (WT) and Xuzhou142 fiberless and fuzzless (*fl*) mutant. **a** Transcript levels of 72 *GhSAURs* in three stages of two BILs. **b** Transcript levels of 40 *GhSAURs* in −3 and 0 DPA ovules of Xuzhou142 (WT) and its *fl* mutant. **c** The number of differentially expressed *SAUR* genes between different stages of BILs NMGA-062 with longer fibers (L) and NMGA-105 with shorter fibers (S). For example, L0 represents 0 DPA ovules of NMGA-062. **d** The number of differentially expressed *SAUR* genes between different stages of Xuzhou142 (WT) and Xuzhou142 *fl* mutant (*fl*). For example, WT_-3 represents −3 DPA ovules of Xuzhou142

The RNA-seq transcriptome data from the two backcross inbred lines (BILs) NMGA-062 and NMGA-105 at different developmental stages (0 DPA and 3 DPA ovules, and 10 DPA fibers) and Xuzhou142 and Xuzhou142 *fl* mutant at −3 and 0 DPA ovules were used to analyze the expression patterns of candidate *GhSAUR* genes in Upland cotton. The NMGA-062 had a greater fiber length than NMGA-105. Among the 214 *GhSAURs*, 72 genes had an FPKM ≥1 in at least one of the three developmental stages of the two BILs and were used to analyze the relative expression of each gene. Based on a cluster analysis, these *SAUR* genes showed four major patterns (Fig. 6a). The first group is composed of 20 genes showing an overall higher level in 0 DPA and 3 DPA ovules and 10 DPA fibers, when compared with other genes. In the second group, 25 genes showed a progressive decrease from 0 DPA to 10 DPA, while 14 genes increased from 0 DPA to 10 DPA in the third group. The other 13 genes in group four showed an overall lower expression level. With the absolute value of log2-fold change ≥1 which was also statistically significant as the standard to judge differently expressed genes (DEGs), we found that 6 *GhSAUR* genes were DEGs when compared between the two BILs at the same development stage, while 30 *GhSAUR* genes were found to be DEGs among the three different developmental stages (Fig. 6c). As shown in Fig. 6, common DEGs were detected among different comparisons.

In another RNA-seq transcriptome profiling, only 40 *SAUR* genes were found to be expressed in −3 or 0 DPA ovules of Xuzhou142 and its *fl* mutant (Fig. 6b). Genes showed a relatively higher level of expression in group one than in group two. Of 10 genes showing significant differential expressions, 2 and 4 DEGs were detected between Xuzhou 142 (WT) and its *fl* mutant, at −3 and 0 DPA ovules, respectively, and 6 and 5 DEGs were detected between −3 and 0 DPA ovules in Xuzhou 142 (WT) and its *fl* mutant, respectively. Interestingly,3 of the 5-6 DEGs between −3 and 0 DPA ovules are in common in the two genotypes (Fig. 6d).

**Fig. 7:**
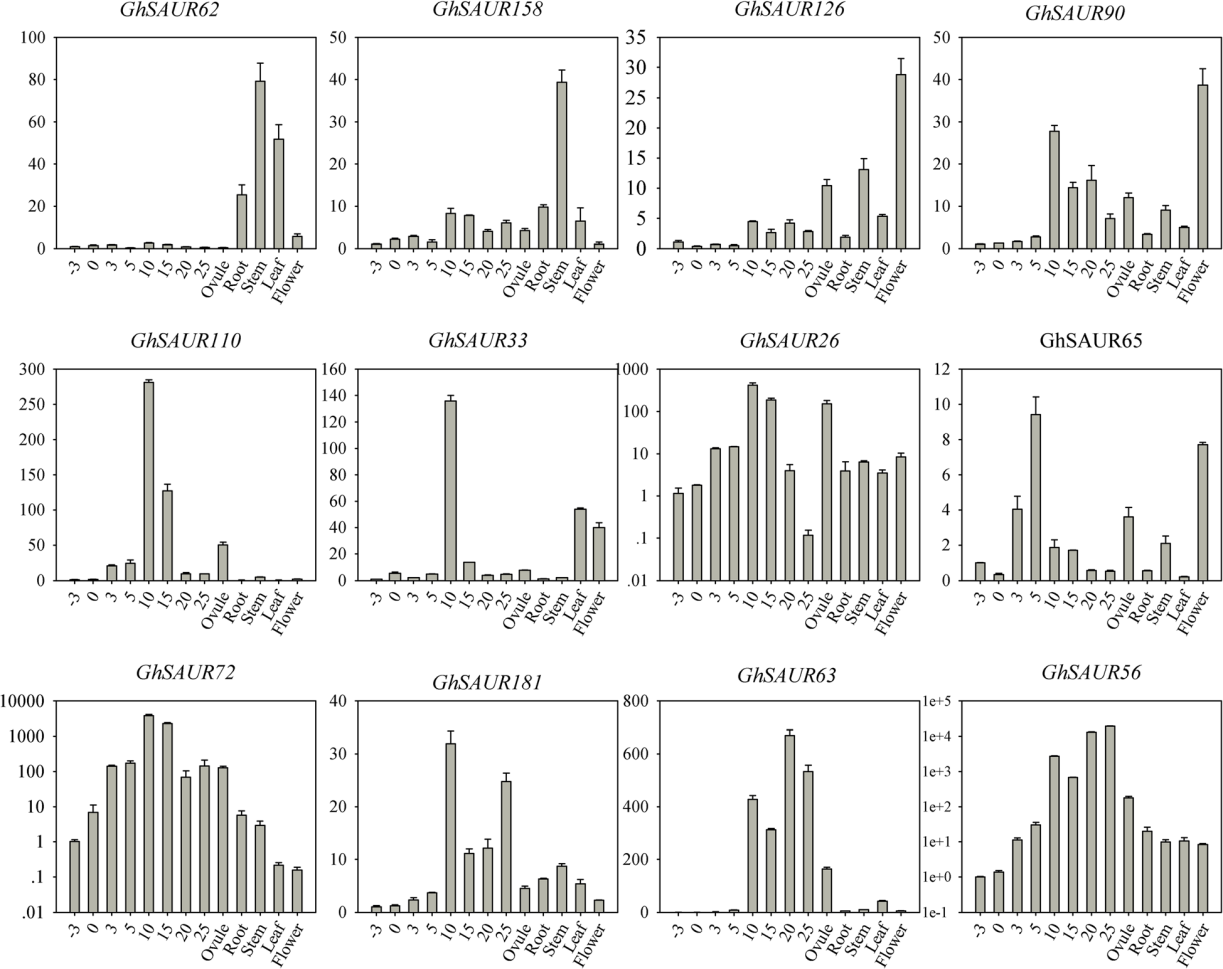
Expression patterns of *GhSAUR* genes in different tissues and developmental stages of NMGA-062 based on quantitative RT-PCR (qRT-PCR). The x-axis represents different developmental stages (−3, 0, and 3 DPA ovules; 5, 10, 15, 20, and 25 DPA fibers; Ovule, 10 DPA ovule; Root; Stem; Leaf; Flower), and the y-axis indicates the relative expression levels as determined by qRT-PCR. The error bars shown are the standard deviation of three biological replicates

To further study the expression profiles based on RNA-seq, quantitative RT-PCR (qRT-PCR) was conducted for 12 *SAUR* genes in 5 organs and 8 fiber developmental stages of NMGA-062 (Fig. 7). The results showed that some *SAUR* genes exhibited diverse expression profiles, while others showed similar expression patterns. Specifically, four genes, *GhSAUR62*, *GhSAUR158*, *GhSAUR126*, and *GhSAUR90*, were exclusively highly expressed in stems or flowers. The expression of *GhSAUR110*, *GhSAUR33*, *GhSAUR26*, *GhSAUR65*, *GhSAUR72*, and *GhSAUR181* increased between −3 and 20 DPA in that it peaked in fibers at 10 or 15 DPA, and then decreased at 20 DPA fibers. Another two genes, *GhSAUR63* and *GhSAUR56*, were increased from −3 to 25 DPA with high expressions at 25 DPA fibers.

### Co-localization of *SAURs* with QTL for FL

**Fig. 8:**
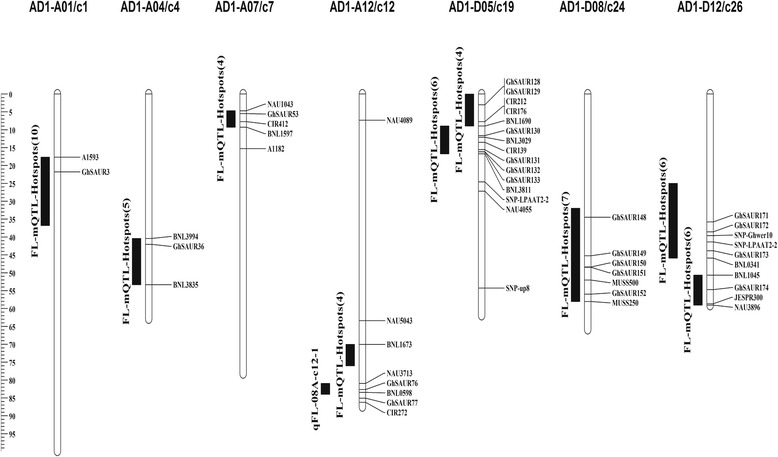
A co-localization analysis of *SAUR* genes with fiber length quantitative trait loci (QTL). Only genes co-localized with the FL QTL are shown in the figure

To better understand the potential function of *SAUR* genes related to fiber length (FL), we co-localized the *SAUR* genes with reported FL quantitative trait loci (QTL). As a result, 20 genes were mapped with the anchored FL QTL or FL QTL hotspots within a 25-cM region (Fig. 8). There was 1 gene (*GhSAUR3*) on chromosome AD1-A01, 1 gene (*GhSAUR36*) on AD1-A04, 1 gene (*GhSAUR53*) on AD1-A07, and 2 genes (*GhSAUR76* and *GhSAUR77*) on AD1-A12 located within the FL QTL hotspots in the At subgenome. While 6 genes (i.e., *GhSAUR128*, *GhSAUR129*, *GhSAUR130*, *GhSAUR131*, *GhSAUR132*, and *GhSAUR133*) on chromosome AD1-D05, 5 genes (i.e., *GhSAUR148*, *GhSAUR149*, *GhSAUR150*, *GhSAUR151*, and *GhSAUR152*) on AD1-D08, 4 genes (i.e., *GhSAUR171*, *GhSAUR172*, *GhSAUR173*, and *GhSAUR174*) on AD1-D12 were located within the FL QTL hotspots in the Dt subgenome. Of these co-localized *SAUR* genes, only 3 genes were differentially expressed between the two BILs differing in fiber length. For example, *GhSAUR149* was up-regulated in 10 DPA fibers of the BIL with longer fibers as compared with the BIL with shorter fibers, and its expression in the long fiber BIL was also higher in 10 DPA fibers than in 0 DPA ovules. However, *GhSAUR148* in the long fiber BIL was down-regulated at 3 DPA ovules than at 0 DPA ovules; And *GhSAUR173* in the long fiber BIL was also down-regulated at 3 ovules and 10 DPA fibers than 0 DPA ovules.

### Identification of single nucleotide polymorphisms (SNP) in *SAUR* genes co-localized with FL

Sequence variations in the predicted *SAUR* genes among the sequenced *G. raimondii* (D5), *G. arboreum* (A2, Shixiya1), *G. hirsutum* (AD1, TM-1) and *G. barbadense* (AD2, 3-79 and Xinhai 21) were further analyzed (Additional file 12: Figure S5). 12 genes had 1-7 SNPs between AD1 and AD2, while other 8 genes were identical between AD1 and AD2. Among the SNP-containing *SAUR* genes, *GhSAUR3* and *GhSAUR77* from AD1 on the At subgenome have identical SNP sequences to the homologous genes in A2. Among 9 genes with homologous genes on D5, the SNP sites of *GhSAUR174* in AD2 were identical to the homologous D5 genes, while the SNP sites of *GhSAUR132*, *GhSAUR133*, *GhSAUR149* and *GhSAUR171* in AD1 were identical to the homologous genes in D5. Interestingly, the SNP sites of homologous *GhSAUR128*, *GhSAUR148*, *GhSAUR150* and *GhSAUR152* genes in both AD1 and AD2 shared with D5 depending on SNP sites. 7 SNPs in *GhSAUR53* from the At subgenome were detected between AD1 and AD2, but its sequence was different from both A2 and D5.

To understand if the co-localized *SAURs* are genetically associated with fiber elongation, a sequence comparison between NMGA-062 and NMGA-105 was performed, but no SNPs were identified. The results indicated that *SAURs* are not genetically related to fiber length, implying that the differences in fiber length between the two species are unlikely related to the natural sequence variations in the *SAUR* genes.

## Discussion

### *SAUR* gene family in cotton

Previous reports have suggested that the *SAUR* family regulates a series of cellular, physiological, and developmental processes in response to hormonal and environmental signals in higher plants [1]. However, the molecular network that links specific hormonal and environmental signals is still unknown. With the availability of genome sequences, a genome-wide identification and annotation of *SAUR* genes has been performed in *Arabidopsis* (72), rice (58), sorghum (71), tomato (99), potato (134), maize (79), citrus (70), mulberry (62), hemp (56), and ramie (71) [3, 6–11]. In this study, 145, 97, 214, and 176 *SAUR* genes in four sequenced cotton species, *G. raimondii*, *G. arboreum*, *G. hirsutum*, and *G. barbadense*, respectively, were identified *in-silico* and characterized. Compared with most of the *SAUR* gene family numbers in other reported species, more members were existent in cotton. It suggests that the *SAUR* family in cotton experienced an extensive expansion during its evolutionary history. It was reported that tandem and segmental duplication events contributed to the expansion of the *SAUR* family in Solanaceae species and maize [8, 9]. In this current study, 55.7 - 87.4% of the *SAUR* gene family members in cotton were likely from tandem duplication and segmental duplication events. The duplication events occurred about 115-146 and 13-20 million year ago in *G. arboreum* and *G. raimondii*, respectively, which was followed by the formation of the *G. hirsutum* and *G. barbadense* from the hybridization of the two extant progenitors relatives and polyploidization event 1.5 million years ago [23, 26]. Therefore, the *SAUR* gene number in the tetraploids *G. hirsutum* and *G. barbadense* is likely to depend on the number of *G. arboreum* and *G. raimondii*. Segmental duplications can further contribute to the expansion of the *SAUR* family. The chromosomal distribution of *SAUR* gene family also showed that genes in this family were not randomly distributed on the genome, but in tandem arrays of extremely related paralogous genes, as reported in other species [3, 6]. Thus, tandem duplication and segmental duplication events also contributed to expansion of *SAUR* family in cotton, as other reported gene families [30, 32].

### Genomic structure of *SAUR* family genes

The majority of *SAUR* gene family lacks introns. Only one *SAUR* gene in *Arabidopsis* has an intron, while none of the *OsSAURs* in rice harbors any intron. Among other species, 6 out of 58 *SAUR* genes in maize, 3 out of 99 *SAUR* genes in tomato, 9 out of *SAUR* genes in potato, 10 out of 70 *SAUR* genes in citrus contained introns based on the sequenced genomes [3, 6, 8–10]. Similar phenomenon in cotton was found in this study in that about 9.3% of the *SAUR* genes in cotton carried introns. As the occurrence of alternative splice in intronless genes is usually low, the function of certain *SAUR* family genes is likely stable.

The motif analysis of the sequenced cotton species showed the existence of the conserved 60 amino acid domain with three motifs specific to the SAUR proteins, similar to SAURs in tomato, potato, tobacco, rice, sorghum and *Arabidopsis* [8]. Although SAUR proteins have variable N- and C-terminal extensions, the relatively short sequence lengths render a high level of similarity between *SAUR* genes.

### Expression profiles and putative functions of GhSAURs

As an important type of auxin responsive genes, *SAURs* participate in the auxin signal pathway. The cis-element analysis in putative promoter regions of the *GhSAURs* showed that most genes possess at least one type of auxin-responsive cis-elements. We analyzed the expression levels of randomly chosen 16 *GhSAUR* genes under IAA treatment. The expression levels of 11 analyzed genes were up-regulated, while 3 were down-regulated after the treatment. This confirmed previous reports that *SAUR* genes were upregulated or repressed to some extent following an auxin treatment [1, 11].

Auxin plays an important role in fiber development, as shown by a previous report that the lint yield and fiber fineness were improved with the overexpression of the IAA biosynthetic gene *iaaM*, driven by the promoter from the petunia MADS box gene Floral Binding protein 7 [22]. The application of auxin increased fiber units and promoted fiber initiation in in-vitro cultured cotton ovules [20]. In this study, the expression profiles of *SAUR* genes in two BILs (differing in fiber length) were analyzed in fiber initiation and elongation stage. We found many *SAUR* genes in group II and III showed differential expressions from 0 DPA to 10 DPA (Fig. 6). We suggest that these genes may be regulated during fiber initiation and elongation. We also showed that several genes were differentially expressed at the fiber initiation stage between Xuzhou142 and its *fl* mutant.

We also investigated the gene expression patterns of 12 *SAUR* genes in various tissues. As Fig. 7 showed, apart from four genes that had a relatively high expression level in stems and flowers, the other tested genes had high levels in different fiber development stages (−3 to 25 DPA). The alignment of these genes with *Arabidopsis* and rice *SAUR* genes showed that *GhSAUR33* had a high similarity with *AtSAUR61*-*AtSAUR68* subfamily and *OsSAUR54*. In *Arabidopsis,* transgenic plants expressing *SAUR63:GFP* or *SAUR63:GUS* fusions had long hypocotyls, petals and stamen filaments, while overexpressed artificial microRNAs targeting *SAUR63* subfamily led to reduced hypocotyls and stamen filament elongation. The results indicated that these *AtSAUR* genes regulated cell expansion to change the hypocotyl growth [15]. *OsSAUR54* was preferentially expressed in rice stigma, and may promote pollen tube growth [47]. Cotton fibers are single-celled trichomes, and grow via a similar mode to pollen tubes in that the fiber cell elongation depends on cell expansion [48]. Our qRT-PCR result and the function of orthologous genes of *GhSAUR33* indicated that it may have a similar function in cotton fiber elongation.

In cotton, the auxin signaling pathway was associated with the dedifferentiation and redifferentiation during somatic embryogenesis in a transcript profiling analysis on *SAUR* genes [49]. In another study, *SAUR* genes were found down-regulated in a dwarf cotton genotype when compared with the wild type, indicating their involvement in the growth of plant height [50]. In *Arabidopsis*, *SAUR* genes were reported to regulate plant growth and development via regulating cell expansion [12–14], shade avoidance responses [51], tropic growth [52], root growth [18], auxin transport [15], and leaf growth and senescence [16]. As a plant specific gene family, *SAURs* in cotton are also likely to have diverse functions. However, no other researches about *SAUR* genes were reported in cotton except for the above two reports. Therefore, our study provides an important piece of information that will facilitate our understanding of specific functions of *SAURs* in cotton growth and development.

## Conclusions

This study provides a comprehensive analysis of *SAUR* gene family in sequenced genomes of four cotton species for the first time. The phylogenetic analysis of *SAUR*s classified the *SAUR* genes into 10 groups. A chromosomal location and gene duplication analysis revealed that duplication events have contributed to the expansion of the *SAUR* gene family in cotton. Most studied *GhSAUR* genes showed differential expressions in leaves in response to auxin applications. A further expression analysis using RNA-seq transcriptome and qRT-PCR showed various expression patterns of *SAUR* genes in early developmental ovules and fibers. Although 20 *SAURs* are co-localized with fiber length quantitative trait loci (QTL), no sequence variations were identified between two interspecific backcross inbred lines (BILs) with different fiber length, suggesting an unlikely genetic involvement of these *SAURs* genes in fiber elongation.

## Additional files

Additional file 1: Table S1. Primers used for qRT-PCR analysis. (XLSX 9 kb)

Additional file 2: Table S2. *SAUR* gene family in *Gossypium arboreum*. (XLS 47 kb)

Additional file 3: Table S3. *SAUR* gene family in *Gossypium hirsutum*. (XLSX 28 kb)

Additional file 4: Table S4. *SAUR* gene family in *Gossypium barbadense*. (XLSX 25 kb)

Additional file 5: Table S5. The number of *SAUR* genes from 12 species classified into groups I - X. (XLSX 9 kb)

Additional file 6: Table S6. The paralogous pairs of *SAUR* genes in *Gossypium raimondii*, *G. arboreum*, *G. hirsutum*, and *G. barbadense*. (XLSX 12 kb)

Additional file 7: Figure S1. Phylogenetic relationships, gene structure and motif compositions of the *Gossypium arboreum SAUR* genes. (A) The phylogenetic tree was constructed using MEGA 6.0 with the Neighbour-Joining (NJ) method with 1000 bootstrap replicates. (B) Exon/intron structures of *SAUR* genes from *G. arboreum*. The introns, CDS are represented by black lines, green and blue boxes respectively. The scale bar represents 0.5 kb. (C) Protein motif. Each motif is represented in the colored box. (PDF 2081 kb)

Additional file 8: Figure S2. Phylogenetic relationships, gene structure and motif compositions of the *Gossypium hirsutum SAUR* genes. (A) The phylogenetic tree was constructed using MEGA 6.0 with the Neighbour-Joining (NJ) method with 1000 bootstrap replicates. (B) Exon/intron structures of *SAUR* genes from *G. hirsutum*. The introns, CDS are represented by black lines, green and blue boxes respectively. The scale bar represents 0.5 kb. (C) Protein motif. Each motif is represented in the colored box. (PDF 509 kb)

Additional file 9: Figure S3. Phylogenetic relationships, gene structure and motif compositions of the *Gossypium barbadense SAUR* genes. (A) The phylogenetic tree was constructed using MEGA 6.0 with the Neighbour-Joining (NJ) method with 1000 bootstrap replicates. (B) Exon/intron structures of *SAUR* genes from *G. hirsutum*. The introns, exons and UTRs are represented by black lines, green and blue boxes respectively. The scale bar represents 0.5 kb. (C) Protein motif. Each motif is represented in the colored box. (PDF 677 kb)

Additional file 10: Figure S4. A multiple sequence alignment of histidine-rich SAUR proteins in *Gossypium raimondii*, *G. arboreum*, *G. hirsutum*, and *G.* ***barbadense*** **.** The histidine-rich regions are indicated by lines above the sequences. (PDF 769 kb)

Additional file 11: Table S7. Cis-elements in the promoters of *GhSAUR* genes. (XLSX 15 kb)

Additional file 12: Figure S5. Sequence alignment of the predicted *SAUR* genes between four sequenced *Gossypium* species. The red boxes indicate sequence variations of *G. hirsutum* (TM-1) and *G. barbadense* (3-79 and Xinhai 21). Gh1 indicate TM-1 sequenced by Zhang et al. (2015). Gb1 and Gb2 indicate 3-79 and Xinhai21 sequenced by Yuan et al. (2015) and Liu et al. (2015), respectively. Gr and Ga indicate two diploid genomes sequenced by Paterson et al. (2013) and Li et al. (2014), respectively. (PDF 335 kb)

## Abbreviations

BILs: Backcross inbred lines
DEGs: Differentially expressed genes
DPA: Days post anthesis
FL: Fiber length
FPKM: Fragments per kilobase of transcript per million fragments
QTL: Quantitative trait loci
SAUR: Small auxin-up RNA
SNP: Single nucleotide polymorphism

## References

[1] Ren H, Gray WM (2015). SAUR proteins as effectors of hormonal and environmental signals in plant growth. Mol Plant.

[2] Abel S, Oeller PW, Theologis A (1994). Early auxin-induced genes encode short-lived nuclear proteins. Proc Natl Acad Sci U S A.

[3] Hagen G, Guilfoyle T (2002). Auxin-responsive gene expression: genes, promoters and regulatory factors. Plant Mol Biol.

[4] Newman TC, Ohme-Takagi M, Taylor CB, Green PJ (1993). DST sequences, highly conserved among plant SAUR genes, target reporter transcripts for rapid decay in tobacco. Plant Cell.

[5] McClure BA, Guilfoyle T (1987). Characterization of a class of small auxin-inducible soybean polyadenylated RNAs. Plant Mol Biol.

[6] Jain M, Tyagi AK, Khurana JP (2006). Genome-wide analysis, evolutionary expansion, and expression of early auxin-responsive SAUR gene family in rice (*Oryza sativa*). Genomics.

[7] Wang S, Bai Y, Shen C, Wu Y, Zhang S, Jiang D, Guilfoyle TJ, Chen M, Qi Y (2010). Auxin-related gene families in abiotic stress response in Sorghum Bicolor. Funct Integr Genomics.

[8] Wu J, Liu S, He Y, Guan X, Zhu X, Cheng L, Wang J, Lu G (2012). Genome-wide analysis of SAUR gene family in Solanaceae species. Gene.

[9] Chen Y, Hao X, Cao J (2014). Small auxin upregulated RNA (SAUR) gene family in maize: identification, evolution, and its phylogenetic comparison with Arabidopsis, rice, and sorghum. J Integr Plant Biol.

[10] Xie R, Dong C, Ma Y, Deng L, He S, Yi S, Lv Q, Zheng Y (2015). Comprehensive analysis of SAUR gene family in citrus and its transcriptional correlation with fruitlet drop from abscission zone A. Funct Integr Genomics.

[11] Huang X, Bao Y, Wang BO, Liu L, Chen J, Dai L, Baloch SU, Peng D (2016). Identification of small auxin-up RNA (SAUR) genes in Urticales plants: mulberry (Morus Notabilis), hemp (*Cannabis sativa*) and ramie (*Boehmeria nivea*). J Genet.

[12] Spartz AK, Lee SH, Wenger JP, Gonzalez N, Itoh H, Inze D, Peer WA, Murphy AS, Overvoorde PJ, Gray WM (2012). The SAUR19 subfamily of SMALL AUXIN UP RNA genes promote cell expansion. Plant J.

[13] Spartz AK, Ren H, Park MY, Grandt KN, Lee SH, Murphy AS, Sussman MR, Overvoorde PJ, Gray WM (2014). SAUR inhibition of PP2C-D phosphatases activates plasma membrane H+−ATPases to promote cell expansion in Arabidopsis. Plant Cell.

[14] Spartz AK, Lor VS, Ren H, Olszewski NE, Miller ND, Wu G, Spalding EP, Gray WM: Constitutive expression of Arabidopsis small Auxin up RNA19 (SAUR19) in tomato confers auxin-independent hypocotyl elongation. Plant Physiol. 2017;173:1453-62. 10.1104/pp.16.01514 PMC5291034 27999086

[15] Chae K, Isaacs CG, Reeves PH, Maloney GS, Muday GK, Nagpal P, Reed JW (2012). Arabidopsis SMALL AUXIN UP RNA63 promotes hypocotyl and stamen filament elongation. Plant J.

[16] Hou K, Wu W, Gan SS (2013). SAUR36, a small auxin up RNA gene, is involved in the promotion of leaf senescence in Arabidopsis. Plant Physiol.

[17] Kant S, Bi YM, Zhu T, Rothstein SJ (2009). SAUR39, a small auxin-up RNA gene, acts as a negative regulator of auxin synthesis and transport in rice. Plant Physiol.

[18] Li ZG, Chen HW, Li QT, Tao JJ, Bian XH, Ma B, Zhang WK, Chen SY, Zhang JS (2015). Three SAUR proteins SAUR76, SAUR77 and SAUR78 promote plant growth in Arabidopsis. Sci Rep.

[19] Sun N, Wang J, Gao Z, Dong J, He H, Terzaghi W, Wei N, Deng XW, Chen H (2016). Arabidopsis SAURs are critical for differential light regulation of the development of various organs. Proc Natl Acad Sci U S A.

[20] Beasley CA (1973). Hormonal regulation of growth in unfertilized cotton ovules. Science.

[21] Guinn G, Brummett DL (1988). Changes in abscisic acid and indoleacetic acid before and after anthesis relative to changes in abscission rates of cotton fruiting forms. Plant Physiol.

[22] Zhang M, Zheng X, Song S, Zeng Q, Hou L, Li D, Zhao J, Wei Y, Li X, Luo M (2011). Spatiotemporal manipulation of auxin biosynthesis in cotton ovule epidermal cells enhances fiber yield and quality. Nat Biotechnol.

[23] Li F, Fan G, Lu C, Xiao G, Zou C, Kohel RJ, Ma Z, Shang H, Ma X, Wu J (2015). Genome sequence of cultivated Upland cotton (*Gossypium hirsutum* TM-1) provides insights into genome evolution. Nat Biotechnol.

[24] Zhang T, Hu Y, Jiang W, Fang L, Guan X, Chen J, Zhang J, Saski CA, Scheffler BE, Stelly DM (2015). Sequencing of allotetraploid cotton (*Gossypium hirsutum* L. acc. TM-1) provides a resource for fiber improvement. Nat Biotechnol.

[25] Yuan D, Tang Z, Wang M, Gao W, Tu L, Jin X, Chen L, He Y, Zhang L, Zhu L (2015). The genome sequence of Sea-Island cotton (*Gossypium barbadense*) provides insights into the allopolyploidization and development of superior spinnable fibres. Sci Rep.

[26] Liu X, Zhao B, Zheng HJ, Hu Y, Lu G, Yang CQ, Chen JD, Chen JJ, Chen DY, Zhang L (2015). *Gossypium barbadense* genome sequence provides insight into the evolution of extra-long staple fiber and specialized metabolites. Sci Rep.

[27] Paterson AH, Wendel JF, Gundlach H, Guo H, Jenkins J, Jin D, Llewellyn D, Showmaker KC, Shu S, Udall J (2012). Repeated polyploidization of Gossypium genomes and the evolution of spinnable cotton fibres. Nature.

[28] Wang K, Wang Z, Li F, Ye W, Wang J, Song G, Yue Z, Cong L, Shang H, Zhu S (2012). The draft genome of a diploid cotton *Gossypium raimondii*. Nat Genet.

[29] Li F, Fan G, Wang K, Sun F, Yuan Y, Song G, Li Q, Ma Z, Lu C, Zou C (2014). Genome sequence of the cultivated cotton *Gossypium arboreum*. Nat Genet.

[30] Wang X, Ma Q, Dou L, Liu Z, Peng R, Yu S (2016). Genome-wide characterization and comparative analysis of the MLO gene family in cotton. Plant Physiol Biochem.

[31] Zhang X, Wang L, Xu X, Cai C, Guo W (2014). Genome-wide identification of mitogen-activated protein kinase gene family in *Gossypium raimondii* and the function of their corresponding orthologs in tetraploid cultivated cotton. BMC Plant Biol.

[32] Niu E, Cai C, Zheng Y, Shang X, Fang L, Guo W (2016). Genome-wide analysis of CrRLK1L gene family in Gossypium and identification of candidate CrRLK1L genes related to fiber development. Mol Genet Genomics.

[33] Yu J, Jung S, Cheng CH, Ficklin SP, Lee T, Zheng P, Jones D, Percy RG, Main D (2014). CottonGen: a genomics, genetics and breeding database for cotton research. Nucleic Acids Res.

[34] Finn RD, Bateman A, Clements J, Coggill P, Eberhardt RY, Eddy SR, Heger A, Hetherington K, Holm L, Mistry J (2014). Pfam: the protein families database. Nucleic Acids Res.

[35] Artimo P, Jonnalagedda M, Arnold K, Baratin D, Csardi G, de Castro E, Duvaud S, Flegel V, Fortier A, Gasteiger E *et al*: ExPASy: SIB bioinformatics resource portal. Nucleic Acids Res 2012, 40(Web Server issue):W597-W603. http://web.expasy.org/protparam/10.1093/nar/gks400 PMC3394269 22661580

[36] Yu CS, Lin CJ, Hwang JK (2004). Predicting subcellular localization of proteins for gram-negative bacteria by support vector machines based on n-peptide compositions. Protein Sci.

[37] Tamura K, Stecher G, Peterson D, Filipski A, Kumar S (2013). MEGA6: Molecular evolutionary genetics analysis version 6.0. Mol Biol Evol.

[38] Voorrips RE (2002). MapChart: software for the graphical presentation of linkage maps and QTLs. J Hered.

[39] Hu B, Jin JP, Guo AY, Zhang H, Luo JC, Gao G. GSDS 2.0: an upgraded gene feature visualization server. Bioinformatics. 2015;31(8):1296-97. http://gsds.cbi.pku.edu.cn/. 10.1093/bioinformatics/btu817 PMC4393523 25504850

[40] Higo K, Ugawa Y, Iwamoto M, Korenaga T. Plant cis-acting regulatory DNA elements (PLACE) database. Nucleic Acids Research. 1999;27(1):297-300. https://sogo.dna.affrc.go.jp/cgi-bin/sogo.cgi?lang=en&pj=640&action=page&page=newplace. 10.1093/nar/27.1.297 PMC148163 9847208

[41] Yu J, Zhang K, Li S, Yu S, Zhai H, Wu M, Li X, Fan S, Song M, Yang D, et al. Mapping quantitative trait loci for lint yield and fiber quality across environments in a Gossypium hirsutum x Gossypium barbadense backcross inbred line population. Theor Appl Genet. 2013;126(1):275–87. 10.1007/s00122-012-1980-x 23064252

[42] Li X, Wu M, Liu G, Pei W, Zhai H, Yu J, Zhang J, Yu S. Identification of candidate genes for fiber length quantitative trait loci through RNA-Seq and linkage and physical mapping in cotton. BMC Genomics. 2017;18(1):427. 10.1186/s12864-017-3812-5 PMC5452627 28569138

[43] de Hoon MJL, Imoto S, Nolan J, Miyano S: Open source clustering software. Bioinformatics, 2004, 20(9):1453-54. http://bonsai.hgc.jp/~mdehoon/software/cluster/. 10.1093/bioinformatics/bth078 14871861

[44] Guan X, Pang M, Nah G, Shi X, Ye W, Stelly DM, Chen ZJ. miR828 and miR858 regulate homoeologous MYB2 gene functions in Arabidopsis trichome and cotton fibre development. Nat Commun. 2014;5:3050. 10.1038/ncomms4050 24430011

[45] Wang N, Ma J, Pei W, Wu M, Li H, Li X, Yu S, Zhang J, Yu J. A genome-wide analysis of the lysophosphatidate acyltransferase (LPAAT) gene family in cotton: organization, expression, sequence variation, and association with seed oil content and fiber quality. BMC Genomics. 2017;18(1):218. 10.1186/s12864-017-3594-9 PMC5333453 28249560

[46] Said JI, Song M, Wang H, Lin Z, Zhang X, Fang DD, Zhang J. A comparative meta-analysis of QTL between intraspecific Gossypium Hirsutum and interspecific Gossypium hirsutum x G. barbadense populations. Mol Genet Genomics. 2015;290(3):1003–25. 10.1007/s00438-014-0963-9 25501533

[47] Li M, Xu W, Yang W, Kong Z, Xue Y: Genome-wide gene expression profiling reveals conserved and novel molecular functions of the stigma in rice. Plant Physiol. 2007;144(4):1797-812. 10.1104/pp.107.101600 PMC1949881 17556504

[48] Qin YM, Zhu YX. How cotton fibers elongate: a tale of linear cell-growth mode. Curr Opin Plant Biol. 2011;14(1):106–11. 10.1016/j.pbi.2010.09.010 20943428

[49] Yang X, Zhang X, Yuan D, Jin F, Zhang Y, Xu J. Transcript profiling reveals complex auxin signalling pathway and transcription regulation involved in dedifferentiation and redifferentiation during somatic embryogenesis in cotton. BMC Plant Biol. 2012;12:110. 10.1186/1471-2229-12-110 PMC3483692 22817809

[50] An W, Gong W, He S, Pan Z, Sun J, Du X. MicroRNA and mRNA expression profiling analysis revealed the regulation of plant height in Gossypium hirsutum. BMC Genomics. 2015;16:886. 10.1186/s12864-015-2071-6 PMC4628322 26517985

[51] de Wit M, Lorrain S, Fankhauser C: Auxin-mediated plant architectural changes in response to shade and high temperature. Physiol Plant 2014, 151(1):13-24. 10.1111/ppl.12099 24011166

[52] Qiu T, Chen Y, Li M, Kong Y, Zhu Y, Han N, Bian H, Zhu M, Wang J. The tissue-specific and developmentally regulated expression patterns of the SAUR41 subfamily of small auxin up RNA genes: potential implications. Plant Signal Behav. 2013;8(8):e25283. 10.4161/psb.25283 PMC3999058 23759547

