# Supplementary figures and images for: A genome-wide analysis of the small auxin-up RNA (SAUR) gene family in cotton
Source: BMC Genomics. 2017 Oct 23;18:815. doi: 10.1186/s12864-017-4224-2 (PMC5654091; doi:10.1186/s12864-017-4224-2)

A

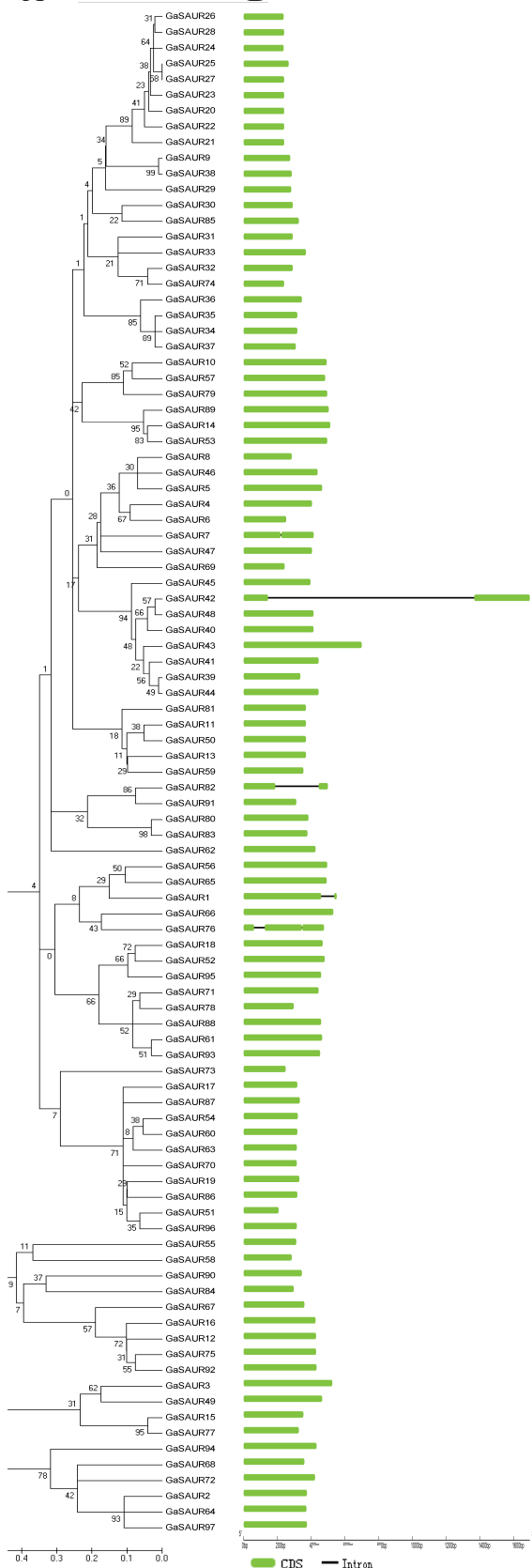

B

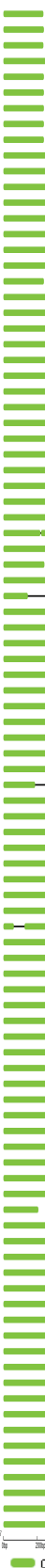

C

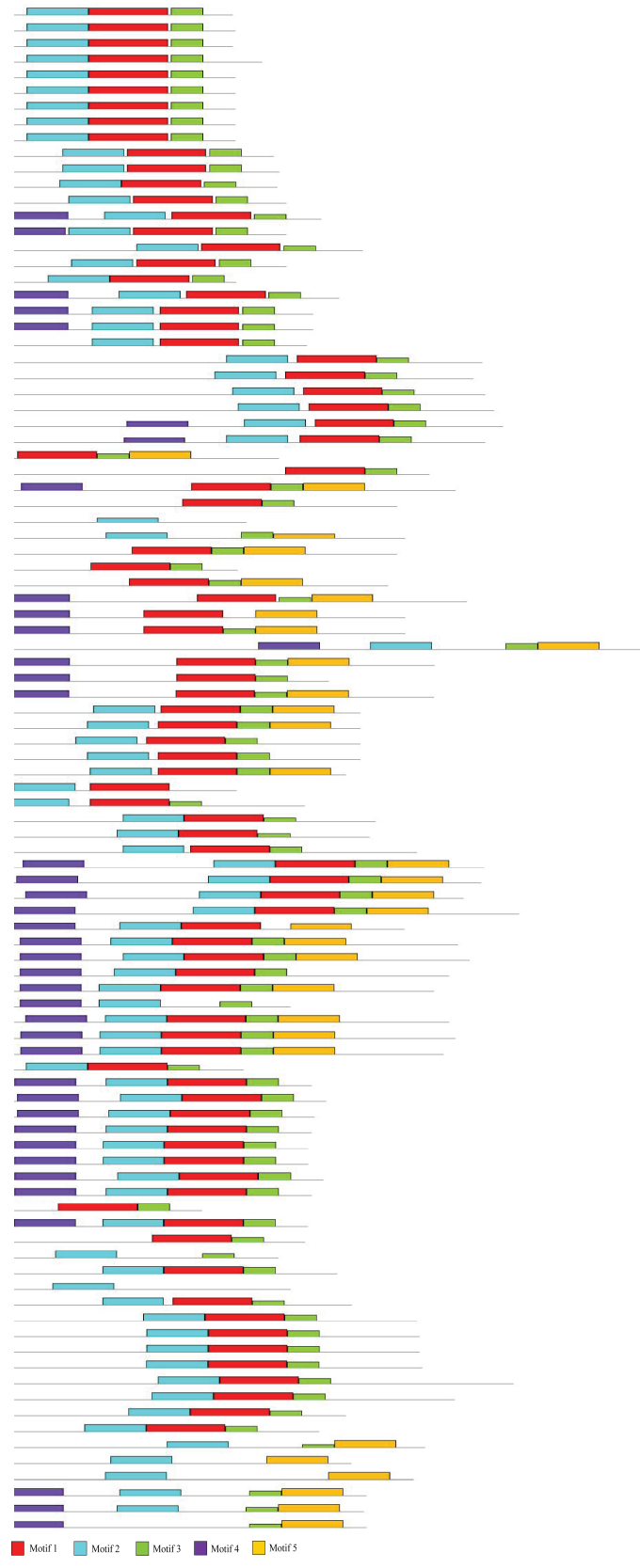

Supplement: Supplementary file 7 — Phylogenetic relationships, gene structure and motif compositions of the Gossypium arboreum SAUR genes. (A) The phylogenetic tree was constructed using MEGA 6.0 with the Neighbour-Joining (NJ) method with 1000 bootstrap replicates. (B) Exon/intron structures of SAUR genes from G. arboreum. The introns, CDS are represented by black lines, green and blue boxes respectively. The scale bar represents 0.5 kb. (C) Protein motif. Each motif is represented in the colored box. (PDF 2081 kb) [file 12864_2017_4224_MOESM7_ESM.pdf]

C

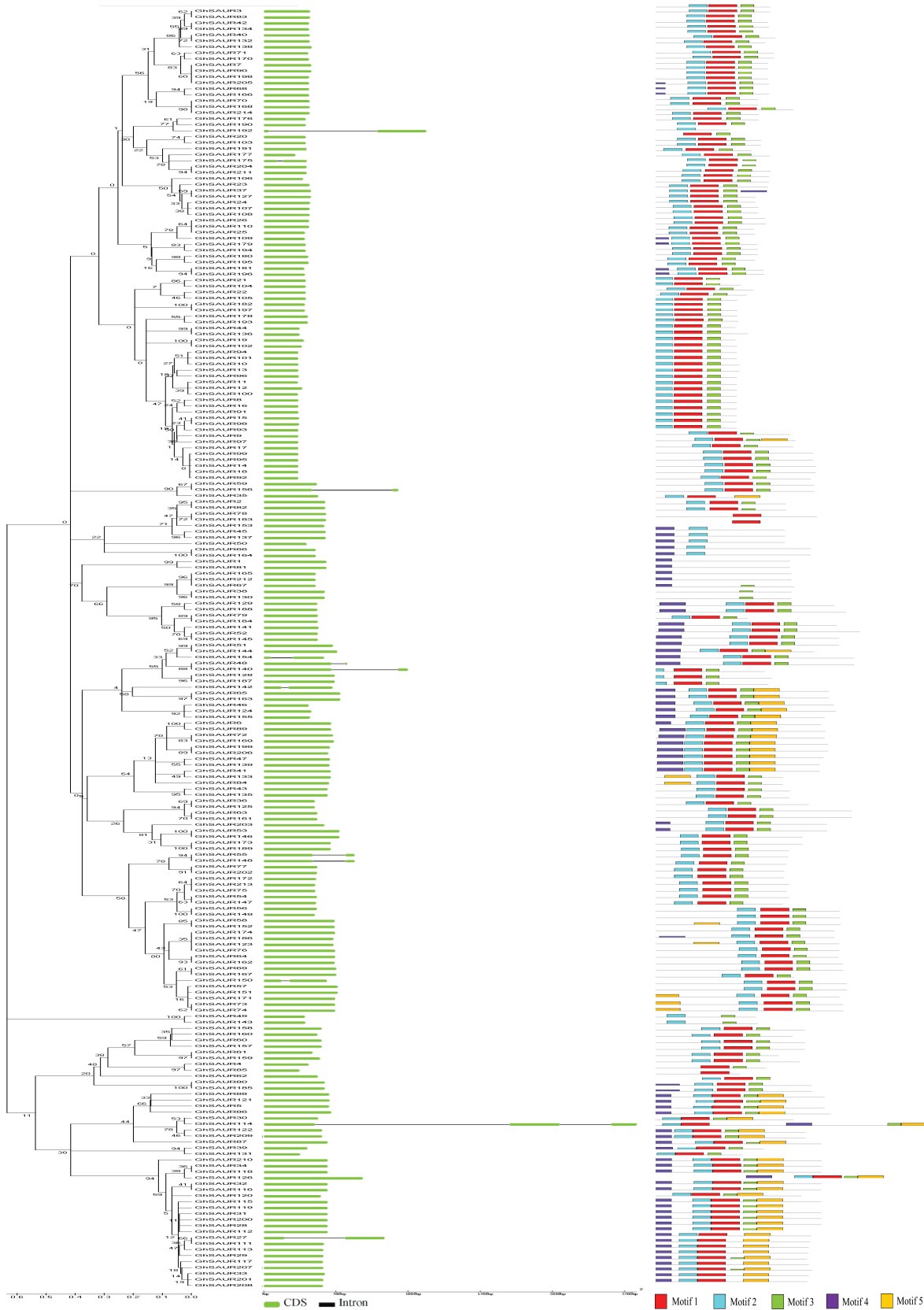

Supplement: Supplementary file 8 — Phylogenetic relationships, gene structure and motif compositions of the Gossypium hirsutum SAUR genes. (A) The phylogenetic tree was constructed using MEGA 6.0 with the Neighbour-Joining (NJ) method with 1000 bootstrap replicates. (B) Exon/intron structures of SAUR genes from G. hirsutum. The introns, CDS are represented by black lines, green and blue boxes respectively. The scale bar represents 0.5 kb. (C) Protein motif. Each motif is represented in the colored box. (PDF 509 kb) [file 12864_2017_4224_MOESM8_ESM.pdf]

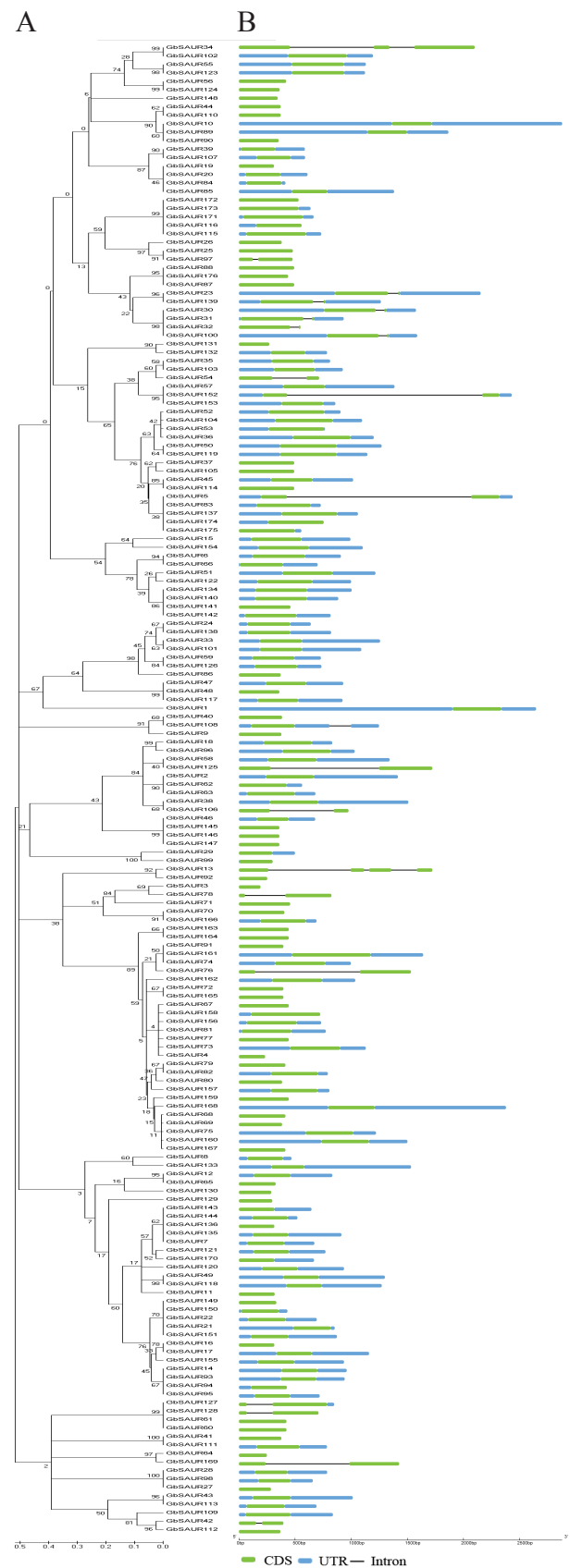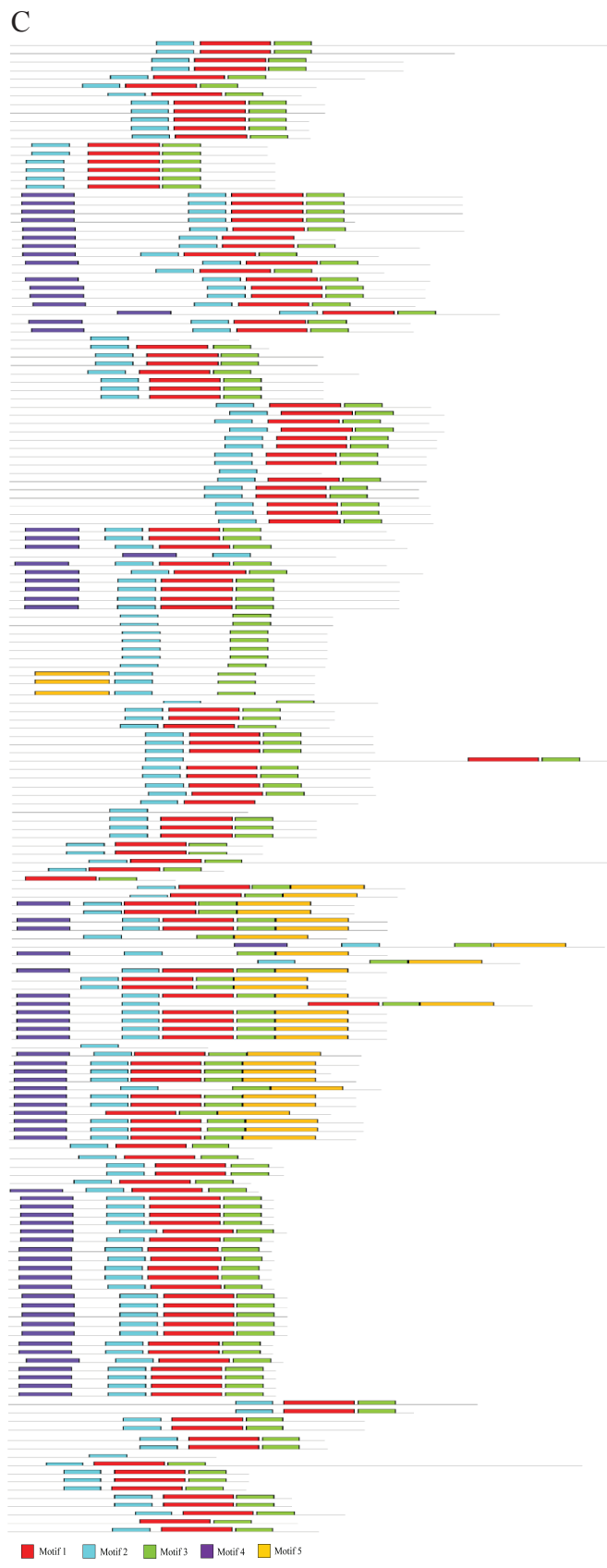

Supplement: Supplementary file 9 — Phylogenetic relationships, gene structure and motif compositions of the Gossypium barbadense SAUR genes. (A) The phylogenetic tree was constructed using MEGA 6.0 with the Neighbour-Joining (NJ) method with 1000 bootstrap replicates. (B) Exon/intron structures of SAUR genes from G. hirsutum. The introns, exons and UTRs are represented by black lines, green and blue boxes respectively. The scale bar represents 0.5 kb. (C) Protein motif. Each motif is represented in the colored box. (PDF 677 kb) [file 12864_2017_4224_MOESM9_ESM.pdf]
