# Supplementary material for: A genome-wide analysis of the small auxin-up RNA (SAUR) gene family in cotton
Source: BMC Genomics. 2017 Oct 23;18:815. doi: 10.1186/s12864-017-4224-2 (PMC5654091; doi:10.1186/s12864-017-4224-2)
Supplement: Supplementary file 10 — A multiple sequence alignment of histidine-rich SAUR proteins in Gossypium raimondii, G. arboreum, G. hirsutum, and G. barbadense . The histidine-rich regions are indicated by lines above the sequences. (PDF 769 kb) [file 12864_2017_4224_MOESM10_ESM.pdf]

```
                         H-rich
GhSAUR149 : MSSGEKSL-----RNFHLHLPHLHHH----HQGKKQTRDVPKGYLAIMVGSQGEER----QRFVVPVMYFNHPLFMRLLK :  67
GrSAUR87  : MGNGEKNS-----RNFHLHIPHLHHHHHH-HQGKKQAKGVPKGCLAIKVGPKEEEQ----QRFVVPVLYFNHPLFMQLLK :  70
GhSAUR202 : MGSGDNNKGSHHHVNFHLHIP--HLHGFH-HHEKKGLKDIPKGCLAIMVG-QGEEQ----QRFVIPVIYINHPLFMQLLK :  72
GaSAUR11  : MGSGEKSF-----RNFHLHLPHLHLHHHQHQQGKKQGSDVPKGCLAIKVGSEGEEQ----QRFVVPVIYFNHPLFMQLLK :  71
GaSAUR81  : MGSGDNNKGSHHHVNFHLHIP--HLHGFH-HHEKKDLKDIPKGCLAIMVG-QGEEQ----QRFVIPVIYINHPLFMQLLK :  72
GhSAUR46  : ------------------------------MEKAK--GKVKKGWLAVEVGLEEEDEQQGFQRFVIPISYLYHPLFKQLLD :  48
GaSAUR50  : MGNGEKNS-----RNFHLHIPHLHHHH----QGKKQAKGVPKGCLAIKVGPKEEEQ----QRFVVPVLYFNHPLFMQLLK :  67
GbSAUR54  : MGNGEKNS-----RNFHLHIPHLHHHHH---QGKKQAKGVPKGCLAIKVGPKEEEQ----QRFVVPVLYFNHPLFMQLLK :  68
GhSAUR155 : ------------------------------MQEDKKATKVKKGWLAVRVGLEEED--GGLKRFAIPISHLYHPLFKQLLD :  48
GhSAUR147 : MGSGEKSF-----RNFHLHLPHLHLHHHQHQQGKKQGSDVPKGCLAIKVGSEGEEQ----QRFVVPVIYFNHPLFMQLLK :  71
GhSAUR213 : MGNGEKNS-----RNFHLHIPHLHHHH----QGKKQAKGVPKGCLAIKVGPKEEEQ----QRFVVPVLYFNHPLFMQLLK :  67
GhSAUR148 : MGIGGDKQ---QHVSFHLHMPNLHLHGFH-HHENKDLKDIPKGCLAVLVG-QGQEQ----QRFVIPLIYITHPLFMQLLK :  71
GaSAUR13  : MSSGEKSL-----RNFQLHLPHLHHHRRHHHQGKKQTRDVPKGYLAIMVGSQGEER----QRFVVPVMCFNHPLFMRLLK :  71
GrSAUR32  : MGSGEKSF-----RNFHLHLPHLHLHHHQHQQGKKQGSDVPKGCLAIKVGSEGEEQ----QRFVVPVIYFNHPLFMQLLK :  71
GbSAUR107 : ------------------------------MQEDKKATTVKKGWLAVRVGLEEED--GGLKRFAIPISHLYHPLFKQLLD :  48
GbSAUR85  : ------------------------------MEKAK--GKVKKGWLAVEVGLEEEDEEQGFQRFVIPISYLYHPLFKQLLD :  48
GbSAUR35  : MGSGEKSF-----RNFHLHLPHLHLHHHQHQQGKKQGSDVPKGCLAIKVGSEGEEQ----QRFVVPVIYFNHPLFMQLLK :  71
GhSAUR75  : MGNGEKNS-----RNFHLHIPHLHHHH----QGKKQAKGVPKGCLAIKVGPKEEEQ----QRFVVPVLYFNHPLFMQLLK :  67
GaSAUR91  : ------------------------------MEKAK--GKVKKGWLAVEVGLEEEDEQHGFQRFVIPISYLYHPLFKQLLD :  48
GhSAUR55  : MGIGGDKQ---QHVSFHLHMPHLHLHGFH-HHENKDLKDIPKGCLAVLVG-QGQEQ----QRFVIPLIYITHPLFMQLLK :  71
GrSAUR33  : MGIGGEKQ---QHVSFHLHMPHLHLHGFH-HHENKDLKDIPKGCLAVLVG-QGQEQ----QRFVIPLIYITHPLFMQLLK :  71
GbSAUR153 : MGSGDNNKGSHHHVNFHLHIP--HLHGFH-HHEKKDLKDIPKGCLAIMVG-QGEEQ----QRFVIPVIYINHPLFMQLLK :  72
GbSAUR57  : MGSGDNNKGSHHHVNFHLHIP--HLHGFH-HHEKKDLKDIPKGCLAIMVG-QGEEQ----QRFVIPVIYINHPLFMQLLK :  72
GrSAUR127 : ------------------------------MEKAK--GKVKKGWLAVEVGLEEEDEEQGFQRFVIPISYLYHPLFKQLLD :  48
GbSAUR39  : ------------------------------MQEDKKATKVKKGWLAVQVGLEEED--GGLKRFAIPISHLYHPLFKQLLD :  48
GhSAUR172 : MGNGEKNS-----RNFHLHIPHLHHHHH---QGKKQAKGVPKGCLAIKVGPKEEEQ----QRFVVPVLYFNHPLFMQLLK :  68
GhSAUR56  : MSSGEKSL-----RNFQLHLPHLHHHCHHHHQGKKQTRDVPKGYLAIMVGSQGEER----QRFVVPVMYFNHPLFMRLLK :  71
GhSAUR54  : MGSGEKSF-----RNFHLHLPHLHLHHHQHQQGKKQGSDVPKGCLAIKVGSEGEEQ----QRFVVPVIYFNHPLFMQLLK :  71
GbSAUR84  : ------------------------------MEKAK--GKVKKGWLAVEVGLEEEDEQQGFQRFVIPISYLYHPLFKQLLD :  48
GbSAUR19  : ------------------------------MEKAK--GKVKKGWLAVEVGLEEEDEQQGFQRFVIPISYLYHPLFKQLLD :  48
GhSAUR77  : MGSGDNNKGSHHHVNFHLHIP--HLHGFH-HHEKKDLKDIPKGCLAIMVG-QGEEQ----QRFVIPVIYINHPLFMQLLK :  72
GbSAUR20  : ------------------------------MEKAK--GKVKKGWLAVEVGLEEEDEQQGFQRFVIPISYLYHPLFKQLLD :  48
GrSAUR92  : MGSGDNNKGSHHHVNFHLHIP--HLHGFH-HHEKKDLKDIPKGCLAIMVG-QGEEQ----QRFVIPVIYINHPLFMQLLK :  72
GrSAUR34  : MSSGEKSL-----RNFHLHLPHLHHH---HHQGKKQTRDVPKGYLAIMVGSQGEER----QRFVVPVMYFNHPLFMRLLK :  68
GaSAUR59  : MGIGGDKQ---QHVSFHLHMPHLHLHGFH-NHENKDLKDIPKGCLAVLVG-QGQEQ----QRFVIPLIYITHPLFMQLLK :  71
GbSAUR103 : MGSGEKSF-----RNFHIHLPHLHLHHHQHQQGKKQGSDVPKGCLAIKVGSEGEEQ----QRFVVPVIYFNHPLFMQLLK :  71
GrSAUR71  : ------------------------------MQEDKKTTKVKKGWLAVRVGLEEED--GGLKRFAIPISHLYHPLFKQLLD :  48
GbSAUR152 : MGSGDNNKGSHHHVNFHLHIP--HLHGFH-HHEKKDLKDIPKGCLAIMVG-QGEEQ----QRFVIPVIYINHPLFMQLLK :  72
GhSAUR124 : ------------------------------MEKAK--GKVKKGWLAVEVGLEEEDEEQGFQRFVIPISYLYHPLFKQLLD :  48

                                                          H-rich
GhSAUR149 : EAEEEYGFDQKGTITIPCHVEEFRNIRGLIDKEKSLHH-----------HHHHHH-----VGCFRV------------- : 117
GrSAUR87  : EAEEEYGFEQKGMITIPCHVEEFRNVRGLIDGEKSLH-----------HHHHHHHHH---VGCFRV------------- : 122
GhSAUR202 : VAEDEYGFDQKGPITIPCHVEEFRNVQGMIDKDRHHHN-------------HHHHHH----VWCFRV------------- : 122
GaSAUR11  : EAEEEYGFDQKGTITIPCNVQEFRNVRGLIDRENSLHQ-----------YHHHHHY----VWCFRV------------- : 122
GaSAUR81  : VAEDEYGFDQKGPITIPCHVEEFRNVQGMIDKDRHHHN-------------HHHHHH----VWCFRV------------- : 122
GhSAUR46  : KAYEVYGYHTKGPIKLPCSVDDFLNLKWQIEKESN-----------HHHHHHHHHHHLPLTLPFHSC------------- : 103
GaSAUR50  : EAEEEYGFEQKGMITIPCHVEEFRNVRGLIDGEKSLH-----------HHHHHHHHHHHVGCFRV------------- : 122
GbSAUR54  : EAEEEYGFEQKGMITIPCHVEEFRNVRGLIDVIQFMVPYIFGWWINGDDYRKENMMTKVDHNCSALIC---------- : 136
GhSAUR155 : KAYEVYGYHMTGPIRLPCSTDDFLSLKWRIERESNHHH---------HHHHHHHHHHPLTLSFNSC------------- : 106
GhSAUR147 : EAEEEYGFDQKGTITIPCNVQEFRNVRGLIDRENSLH-------------HHHHHY----VWCFRV------------- : 120
GhSAUR213 : EAEEEYGFEQKGMITIPCHVEEFRNVRGLIDGEKSLH-----------HHHHHHH----VGCFRV------------- : 118
GhSAUR148 : EAEEEYGFDHEGPITIPCHVQEFRNVQGMIDKDHHHHH-------------HHHGWR----NIKLQAYKRRWELGTFVGL : 134
GaSAUR13  : EAEEEYGFDQKGTITIPCHVEEFRNIRGLIDKEKYFHH-----------HHHHHH----VGCFRV------------- : 122
GrSAUR32  : EAEEEYGFDQKGTITIPCNVQEFRNVRGLIDRENSLH-----------HHHHHHY----VWCFRV------------- : 121
GbSAUR107 : KAYEVYGYHMTGPIRLPCSTDDFLNLKWRIEKESN-------------HHHHHHHHPLTLSFNSC------------- : 100
GbSAUR85  : KAYEVYGYHTKGPIKLPCSVDDFLNLKWQIEKESN-----------HHHHHHHHHHHLPLTLPFHSC------------- : 103
GbSAUR35  : EAEEEYGFDQKGTITIPCNVQEFRNVRGLIDRENSLHQ-----------YHHHHHY----VWCFRV------------- : 122
GhSAUR75  : EAEEEYGFEQKGMITIPCHVEEFRNVRGLIDGEKSLH-----------HHHHHHH----VGCFRV------------- : 118
GaSAUR91  : KAYEVYGYHTKGPIKLPCSVDDFLNLKWQIEKESN-----------HHHHHHHHHHHLPLTLPFHSC------------- : 103
GhSAUR55  : EAEEEYGFDHEGPITIPCHVQEFRNVQGMIDKDHHHHH-------------HHHGWR----NIKLQAYKRRWELGTFLGL : 134
GrSAUR33  : EAEEEYGFDHEGPITIPCHVQEFRNVQGMIDKDHHHHH-------------HHHGWR----NIKLQAYKRRWEFGTFV-- : 132
GbSAUR153 : VAEDEYGFDQKGPITIPCHVEEFRNVQGMIDKDRHHHN-------------HHHHHH----VWCFRV------------- : 122
GbSAUR57  : VAEDEYGFDQKGPITIPCHVEEFRNVQGMIDKDRHHHN-------------HHHHHH----VWCFRV------------- : 122
GrSAUR127 : KAYEVYGYHTKGPIKLPCSVDDFLNLKWQIEKESN-----------YHHHHHHHHHHLPLTLPFHSC------------- : 103
GbSAUR39  : KAYEVYGYHMTGPIRMPCSTDDFLNLKWRIEKESN-------------HHHHHHHHPLTLSFNSC------------- : 100
GhSAUR172 : EAEEEYGFEQKGMITIPCHVEEFRNVRGLIDGEKSLH-----------HHHHHHQH---VGCFRV------------- : 120
GhSAUR56  : EAEEEYGFDQKGTITIPCHVEEFRNIRGLIDKEKYFHH-----------HHHHHH----VGCFRV------------- : 122
GhSAUR54  : EAEEEYGFDQKGTITIPCNVQEFRNVRGLIDRENSLHQ-----------YHHHHHY----VWCFRV------------- : 122
GbSAUR84  : KAYEVYGYHTKGPIKLPCSVDDFLNLKWQIEKESN-----------HHHHHHHHHHHLPLTLPFHSC------------- : 103
GbSAUR19  : KAYEVYGYHTKGPIKLPCSVDDFLNLKWQIEKESN-----------HHHHHHHHHHHLPLTLPFHSC------------- : 103
GhSAUR77  : VAEDEYGFDQKGPITIPCHVEEFRNVQGMIDKDRHHHNH-----------HHHHHH----VWCFRV------------- : 123
GbSAUR20  : KAYEVYGYHTKGPIKLPCSVDDFLNLKWQIEKESN-----------HHHHHHHHHHHLPLTLPFHSC------------- : 103
GrSAUR92  : VAEDEYGFDQKGPITIPCHVEEFRNVQGMIDKDRHHHN-------------HHHHHH----VWCFRV------------- : 122
```
