# Supplementary material for: A genome-wide analysis of the small auxin-up RNA (SAUR) gene family in cotton
Source: BMC Genomics. 2017 Oct 23;18:815. doi: 10.1186/s12864-017-4224-2 (PMC5654091; doi:10.1186/s12864-017-4224-2)
Supplement: Supplementary file 12 — Sequence alignment of the predicted SAUR genes between four sequenced Gossypium species. The red boxes indicate sequence variations of G. hirsutum (TM-1) and G. barbadense (3-79 and Xinhai 21). Gh1 indicate TM-1 sequenced by Zhang et al. (2015). Gb1 and Gb2 indicate 3-79 and Xinhai21 sequenced by Yuan et al. (2015) and Liu et al. (2015), respectively. Gr and Ga indicate two diploid genomes sequenced by Paterson et al. (2013) and Li et al. (2014), respectively. (PDF 335 kb) [file 12864_2017_4224_MOESM12_ESM.pdf]

GhSAUR3

```
Gh1-GhSAUR3          ......ATGGCTATCAGCAGGAAATCAAACAAATTGGCACAAACAGCAATGATAAAGCAAATCCTGAAAAGATGTTCCAGCTTAGGGAAGAAACAAAGCT   94
Ga-GaSAUR54          ......ATGGCTATCAGCAGGAAATCAAACAAATTGGCACAAACAGCAATGATAAAGCAAATCCTGAAAAGATGTTCCAGCTTAGGGAAGAAACAAAGCT   94
Gb1-GbSAUR155        ATGAGAATGGCTATCAGCAGGAAATCAAACAAATTGGCACAAACAGCAATGATAAAGCAAATCCTGAAAAGATGTTCCAGCTTAGGGAAGAAACAAAGCT  100
Gb2-GOBAR_DD27584    ATGAGAATGGCTATCAGCAGGAAATCAAACAAATTGGCACAAACAGCAATGATAAAGCAAATCCTGAAAAGATGTTCCAGCTTAGGGAAGAAACAAAGCT  100

Gh1-GhSAUR3          ACGCTGATGAACAAGGACTCCCTTTGGATGTCCCGAAAGGTCATTTTGTGGTATATGTTGGTGAAAACAGAAGCAGATATATAGTTCCCATCTCCTTCTT  194
Ga-GaSAUR54          ACGCTGATGAACAAGGACTCCCTTTGGATGTCCCGAAAGGTCATTTTGTGGTATATGTTGGTGAAAACAGAAGCAGATATATAGTTCCCATCTCCTTCTT  194
Gb1-GbSAUR155        ACGGTGAAGAACAAGGACTCCCTTTGGATGTCCCGAAAGGTCATTTTGTGGTATATGTTGGTGAAAACAGAAGCAGATATATAGTTCCCATCTCCTTTTT  200
Gb2-GOBAR_DD27584    ACGGTGAAGAACAAGGACTCCCTTTGGATGTCCCGAAAGGTCATTTTGTGGTATATGTTGGTGAAAACAGAAGCAGATATATAGTTCCCATCTCCTTTTT  200

Gh1-GhSAUR3          GACCCGACCCGAGTTTCAAAGCTTGCTTCATCAAGCTGAAGAAGAATTCGGGTTTGATCACGACATGGGCCTCACCATTCCTTGTGAAGAAGTTGTTTTC  294
Ga-GaSAUR54          GACCCGACCCGAGTTTCAAAGCTTGCTTCATGAAGCTGAAGAAGAATTCGGGTTTGATCACGACATGGGCCTCACCATTCCTTGTGAAGAAGTTGTTTTC  294
Gb1-GbSAUR155        GACCCGACCCGAGTTTCAAAGCTTGCTTCATCAGGCTGAAGAAGAATTCGGGTTTGATCACGACATGGGCCTCACCATTCCTTGTGAAGAAGTTCTTTTC  300
Gb2-GOBAR_DD27584    GACCCGACCCGAGTTTCAAAGCTTGCTTCATCAGGCTGAAGAAGAATTCGGGTTTGATCACGACATGGGCCTCACCATTCCTTGTGAAGAAGTTCTTTTC  300

Gh1-GhSAUR3          CAGTCTCTAACGTCCATGCTCAGATA  320
Ga-GaSAUR54          CAGTCTCTAACGTCCATGCTCAGATA  320
Gb1-GbSAUR155        CAGTCTCTAACGTCCATGCTCAGATA  326
Gb2-GOBAR_DD27584    CAGTCTCTAACGTCCATGCTCAGATA  326
```

GhSAUR36

```
Gh1-GhSAUR36         ATGAAGGGGAAATTTCTAAAGGCATGCATTACCAAATGGAGGAAGATGGGGAATAGAGTCATACCTTGTGGCAGTTGCGAATACTGTTATGAGTGGGCAA  100
Ga-GaSAUR15          ATGAAGGGGAAATTTCTAAAGGCATGCATTACCAAATGGAGGAAGATGGGGAATAGAGTCATACCTTGTGGCAGTTGCGAATACTGTTATGAGTGGGCAA  100
Gb1-GbSAUR10         ATGAAGGGGAAATTTCTAAAGGCATGCATTACCAAATGGAGGAAGATGGGGAATAGAGTCATACCTTGTGGCAGTTGCGAATACTGTTATGAGTGGGCAA  100
Gb2-GOBAR_AA02272    ATGAAGGGGAAATTTCTAAAGGCATGCATTACCAAATGGAGGAAGATGGGGAATAGAGTCATACCTTGTGGCAGTTGCGAATACTGTTATGAGTGGGCAA  100

Gh1-GhSAUR36         AGTGGCCTAATACCAAGAATGAAGAGAGTTCAATCCCGAGAGATGTGCCAAAGGGTCACTTGGTAGTATATGTTGGTGAAAACTATAAGAGGTTTGTAAT  200
Ga-GaSAUR15          AGTGGCCTAATACCAAGAATGAAGAGAGTTCAATCCCGAGAGATGTGCCAAAGGGTCACTTGGTAGTATATGTTGGTGAAAACTATAAGAGGTTTGTAAT  200
Gb1-GbSAUR10         AGTGGCCTAATACCAAGAATGAAGAGAGTTCAATCCCGAGAGATGTGCCAAAGGGTCACTTGGTAGTATATGTTGGTGAAAACTATAAGAGGTTTGTAAT  200
Gb2-GOBAR_AA02272    AGTGGCCTAATACCAAGAATGAAGAGAGTTCAATCCCGAGAGATGTGCCAAAGGGTCACTTGGTAGTATATGTTGGTGAAAACTATAAGAGGTTTGTAAT  200

Gh1-GhSAUR36         TAAACTTACCTTGCTCAAGCACCCACTGTTCAAGGCATTGCTAGATCAAGCTCAGGATGAATATGATTTTACCACAGACCCCAAACTCTGCATTCCTTGT  300
Ga-GaSAUR15          TAAACTTACCTTGCTCAAGCACCCACTGTTCAAGGCATTGCTAGATCAAGCTCAGGATGAATATGATTTTACCACAGACCCCAAACTCTGCATTCCTTGT  300
Gb1-GbSAUR10         TAAACTTACCTTGCTCAAGCACCCACTGTTCAAGGCATTGCTAGATCAAGCTCAGGATGAATATGATTTTACCACAGACCCCAAACTCTGCATTCCTTGT  300
Gb2-GOBAR_AA02272    TAAACTTACCTTGCTCAAGCACCCACTGTTCAAGGCATTGCTAGATCAAGCTCAGGATGAATATGATTTTACCACAGACCCCAAACTCTGCATTCCTTGT  300

Gh1-GhSAUR36         GATGAAAGCCTTTTCCTCGAAGTTGTTCGATGCGCTAGCTCTCCTCAAGATTG  353
Ga-GaSAUR15          GATGAAAGCCTTTTCCTCGAAGTTGTTCGATGCGCTAGCTCTCCTCAAGATTG  353
Gb1-GbSAUR10         GATGAAAGCCTTTTCCTCGAAGTTGTTCGATGCGCTAGCTCTCCTCAAGATTG  353
Gb2-GOBAR_AA02272    GATGAAAGCCTTTTCCTCGAAGTTGTTCGATGCGCTAGCTCTCCTCAAGATTG  353
```

GhSAUR53

```
Gh1-GhSAUR53     ATGGATGTTGAAAAGATGAAACTGAAGAAAAACATGTTGGCTAAGGCCTGGGACAGATGCAAATCTCTTGGAAATAGTGGCAAGAACTCATTGGTGAATC  100
Gb1-GbSAUR102    ATGGATGCTGAAAAGTTGAAACTGAAGAAAAACATGTTGGCTAAGGCCTGGGACAGATGCAAATCTCTTGGCAATAGTGGCAAGAACTCATCGGTGAATC  100

Gh1-GhSAUR53     CATTGATGAAGAAGAGCAAATCATGTCATATTCCTTCCTCATCCATGGATGATCATAAAAAGAAAAACAAGGTGGCTCCGGAAGGGTGTTTTTCGGTATA  200
Gb1-GbSAUR102    CATTGATGAAGAAGAGCAAATCATGTCATATTCCTTCCTCATCCATGGAAGATCATAAAAAGAAAAACAAGGTGGCTCCGGAAGGGTGTTTTTCGGTATA  200

Gh1-GhSAUR53     CGTTGGATCTGAAAGGCAAAGATTCGTCATCAAGACCGAATTGGTTAACCATCCATTGTTCAAGATGTTGCTTGAAGACGCTGAGCTTGAATATGGGTTC  300
Gb1-GbSAUR102    CGTTGGATCTGAAAGGCAAAGATTCGTCATCAAGACCGAATTGGTTAACCATCCATTGTTCAAGATGTTGCTTGAAGATGCTGAGCTTGAATATGGGTTC  300

Gh1-GhSAUR53     AATAGTGAAGGTCCTCTTCTTCTTCCTTGTGATGTTGATTTGTTTTATAAAGTTTTGGCAGAGATGGATGATAATGGTGATGATGGAGAAATGAGTACTC  400
Gb1-GbSAUR102    AACAGTGAAGGTCCTCTTCTTCTTCCTTGTGATGTTGATTTGTTTTATAAAGTTTTGGCAGAGATGGATGATAATGGTGATGATGGAGAAATGAGTACTC  400

Gh1-GhSAUR53     GTTTCATTTGTAGCTTTGGGTGCAGTCCTTCTCGTCATCGTTTAAGTTCAAGCAGTATGAATAAAGGATATGGATCGTATAAAGCTCTTTGTTCATCTCC  500
Gb1-GbSAUR102    GTTTCATTTGTAGCTTTGGGTGCAGTCCTTCTCGTCATCGTTTAAGTTCAAGCAGTATGAATAAAGGATATGGATCGTATAAAGCTCTTTGTTCATCTCC  500

Gh1-GhSAUR53     AATGATTAAATTGAATAGTTGTTA  524
Gb1-GbSAUR102    AATGATTAAATTGAATAGTTGTTA  524
```

GhSAUR76

```
Gh1-GhSAUR76        ...ATGAAGAAAATTAGAGGGTTCAAGCTTGGGCGCAAGCTAGTGAAGGTGTTCAAATGGATAATCCCACCTAGAAGAAGAAACTATCGGAATTGTTTCT   97
Gb1-GbSAUR175       ATTATGAAGAAAATTAGAGGGTTCAAGCTTGGGCGCAAGCTAGTGAAGGTGTTCAAATGGATAATCCCACCTAGAAGAAGAAACTATCGGAATTGTTTCT  100
Gb2-GOBAR_AA08791   ...ATGAAGAAAATTAGAGGGTTCAAGCTTGGGCGCAAGCTAGTGAAGGTGTTCAAATGGATAATCCCACCTAGAAGAAGAAACTATCGGAATTGTTTCT   97

Gh1-GhSAUR76        TGAGGCATCCGACTCGAAGTTACAACCCTTTATCAAGACTCTGCTCTTTCGCGACGTTTCTTCGACGGGGAACCAAAAGGCTGTGTAATTCGGACTCGGA  197
Gb1-GbSAUR175       TGAGGCATCCGACTCGAAGTTACAACCCTTTATCAAGACTCTGCTCTTTCGCGACGTTTCTTCGACGGGGAACCAAAAGGCTGTGTAATTCGGACTCGGA  200
Gb2-GOBAR_AA08791   TGAGGCATCCGACTCGAAGTTACAACCCTTTATCAAGACTCTGCTCTTTCGCGACGTTTCTTCGACGGGGAACCAAAAGGCTGTGTAATTCGGACTCGGA  197

Gh1-GhSAUR76        TCCGGGTTACATTCAATTGGGTGAAAAGCGAGTGAAGCGGGTAGGAGTGCCGAAGGGGCACCTTGCAGTGTACGTGGGGGAATCAGACGGTAACATGAGG  297
Gb1-GbSAUR175       TCCGGGTTACATTCAATTGGGTGAAAAGCGAGTGAAGCGGGTAGGAGTGCCGAAGGGGCACCTTGCAGTGTACGTGGGGGAATCAGACGGTAACATGAGG  300
Gb2-GOBAR_AA08791   TCCGGGTTACATTCAATTGGGTGAAAAGCGAGTGAAGCGGGTAGGAGTGCCGAAGGGGCACCTTGCAGTGTACGTGGGGGAATCAGACGGTAACATGAGG  297

Gh1-GhSAUR76        AGGGTGGTAGTGCCTGTGATTTATTTCAATCACCCGCTATTTGGGGAGCTGTTGAAGGAAGCGGAGCTGGTTTACGGGTTCAACCAATCGGGTGGGATCA  397
Gb1-GbSAUR175       AGGGTGGTAGTGCCTGTGATTTATTTCAATCACCCGCTATTTGGGGAGCTGTTGAAGGAAGCGGAGCTGGTTTACGGGTTCAACCAATCGGGTGGGATCA  400
Gb2-GOBAR_AA08791   AGGGTGGTAGTGCCTGTGATTTATTTCAATCACCCGCTATTTGGGGAGCTGTTGAAGGAAGCGGAGCTGGTTTACGGGTTCAACCAATCGGGTGGGATCA  397

Gh1-GhSAUR76        CGTTACCATGTGGGATTTCGGAGTTCGAGAAGGTTAAGATGAGAATTGCCGATTGGGATCATTGTCGACGGAAACAACATCGTCATTATTTTTATTG     494
Gb1-GbSAUR175       CGTTACCATGTGGGATTTCGGAGTTCGAGAAGGTTAAGATGAGAATTGCCGATTGGGATCATTGTCGACGGAAACAACATCGTCATTATTTTTATTG     497
Gb2-GOBAR_AA08791   CGTTACCATGTGGGATTTCGGAGTTCGAGAAGGTTAAGATGAGAATTGCCGATTGGGATCATTGTCGACGGAAACAACATCGTCATTATTTTTATTG     494
```

GhSAUR77

```
Gh1-GhSAUR77   ATGGGGAGTGGAGATAACAACAAAGGTTCCCATCACCATGTGAATTTTCACCTTCATATACCACACCTTCATGGCTTCCATCATCATGAGAAGAAGGACC  100
Ga-GaSAUR81    ATGGGGAGTGGAGATAACAACAAAGGTTCCCATCACCATGTGAATTTTCACCTTCATATACCACACCTTCATGGCTTCCATCATCATGAGAAGAAGGACC  100
Gb1-GbSAUR57   ATGGGGAGTGGAGATAACAACAAAGGTTCCCATCACCATGTGAATTTTCACCTTCATATACCACACCTTCATGGCTTCCATCATCATGAGAAGAAGGACC  100

Gh1-GhSAUR77   TGAAAGATATCCCGAAAGGGTGTTTGGCTATCATGGTAGGTCAAGGTGAAGAGCAACAACGGTTCGTGATTCCGGTGATTTATATTAACCACCCGCTGTT  200
Ga-GaSAUR81    TGAAAGATATCCCAAAAGGGTGTTTGGCTATCATGGTAGGTCAAGGTGAAGAGCAACAACGGTTCGTGATTCCGGTGATTTATATTAACCACCCGCTGTT  200
Gb1-GbSAUR57   TCAAAGATATCCCGAAAGGGTGTTTGGCTATCATGGTAGGTCAAGGTGAAGAGCAACAACGGTTCGTGATTCCGGTGATTTATATTAACCACCCGCTGTT  200

Gh1-GhSAUR77   TATGCAGCTGTTGAAGGTAGCCGAGGATGAGTATGGGTTCGATCAGAAAGGACCTATTACCATTCCTTGCCATGTCGAGGAGTTTCGTAATGTTCAAGGG  300
Ga-GaSAUR81    TATGCAGCTGTTGAAGGTAGCCGAGGATGAGTATGGGTTCGATCAGAAAGGACCTATTACCATTCCTTGCCATGTCGAGGAGTTTCGTAATGTTCAAGGG  300
Gb1-GbSAUR57   TATGCAGCTGTTGAAGGTAGCCGAGGATGAGTATGGGTTCGATCAGAAAGGACCTATTACCATTCCTTGCCATGTCGAGGAGTTTCGTAATGTTCAAGGG  300

Gh1-GhSAUR77   ATGATCGATAAAGATCGTCACCATCATAATCATCATCATCATCATCACCATGTTTGGTGTTTTAGGGTTTG                              371
Ga-GaSAUR81    ATGATCGATAAAGATCGTCACCATCATAATCATCATCATCAT...CACCATGTTTGGTGTTTTAGGGTTTG                              368
Gb1-GbSAUR57   ATGATCGATAAAGATCGTCACCATCATAATCATCATCATCAT...CACCATGTTTGGTGTTTTAGGGTTTG                              368
```

GhSAUR128

```
Gh1-GhSAUR128   ATGGATCATGGCAACAAGTCGACGGGAATCAAGCAGATCGTTAGACTCAAAGGGATGCTTCAAAAATGGCAAACTGCGACATTTAGGTCCCGACCAAATT  100
Gr-GrSAUR93     ATGGATCATGGCAACAAGTCGACGGGAATCAAGCAGATCGTTAGACTCAAAGGGATGCTTCAAAAATGGCAAACTGCGACATTTAGGTCCCGACCAAATT  100
Gb1-GbSAUR88    ATGGATCATGGCAACAAGTCGACGGGAATCAAGCAGATCGTTAGACTCAAAGGGATGCTTCAAAAATGGCAAACTGCGACATTTAGGTCCCGACCAAATT  100

Gh1-GhSAUR128   CCCACAACTCACAACGAAACCATGGCCATGGGGGTGTGTCGCGATCAATCAACAAAGTTCAAACAGAAAAAATGTATTGCGATTCCGACGAGGATGGATG  200
Gr-GrSAUR93     CCCACAACTCACAACGAAACCATGGCCATGGGGGTGTGTCGCGATCAATCAACAAAGTTCAAACAGAAAAAATGTATTGCGATTCCGACGAGGATGGATG  200
Gb1-GbSAUR88    CCCACAACTCACAACGAAACCATGGCCATGGGGGTGTGTCGCGATCAATCAACAAAGTTCAAACAGAAAAAATGTATTGCGATTCCGACGAGGATGGATG  200

Gh1-GhSAUR128   CTACAGCCCTGAACCAGCACCTGATGTTCCAAAAGGATATTTCGCAGTGTATGTAGGGCCTGAGCTTCGAAGGTTCATCATCCCCACCAGCTACCTTAGC  300
Gr-GrSAUR93     CTACAGCCCTGAACCAGCACCTGATGTTCCAAAAGGATATTTCGCAGTGTATGTAGGGCCTGAGCTTCGAAGGTTCATCATCCCCACCAGCTACCTTAGC  300
Gb1-GbSAUR88    CTACAGCCCTGAACCAGCACCTGATGTTCCAAAAGGATATTTCGCAGTGTATGTAGGGCCTGAGCTTCGAAGGTTCATCATCCCCACCAGCTACCTTAGC  300

Gh1-GhSAUR128   CACCCAGTTTTCAAAATATTGCTCCAACAAGCTGAGGAGGAATTCGGATATGATCACAATGGTGCCCTTACTCTCCCTTGTGAGATTGAGACCTTCAAAT  400
Gr-GrSAUR93     CACCCAGTTTTCAAAATATTGCTCCAACAAGCTGAGGAGGAATTCGGATATGATCACAATGGCGCCCTTACTCTCCCTTGTGAGATTGAGACCTTCAAAT  400
Gb1-GbSAUR88    CACCCAGTTTTCAAAATATTGCTCCAACAAGCTGAGGAGGAATTCGGATATGATCACAATGGTGCCCTTACTCTCCCTTGTGAGATTGAGACCTTCAAAT  400

Gh1-GhSAUR128   ATCTCCTCAAATACATAGATCAAAACCAACCTAAGAGTCACAATGTTGGAGGAGGAAGCCCTGTTTCCGAACATGTCCTAATTTGTTTTTA          491
Gr-GrSAUR93     ATCTCCTCAAATACATAGATCAAAACCAACCTAAGAGTCACAATGTTGGAGGAGGAAGCCCTGTTTCTGAACATGTCCTAATTTGTTTTTA          491
Gb1-GbSAUR88    ATCTCCTCAAATACATAGATCAAAACAAACCTAAGAGTCACAATGTTGGAGGAGGAAGCCCTGTTTCTGAACATGTCCTAATTTGTTTTTA          491
```

GhSAUR129

```
Gh1-GhSAUR129       ATGGCGAAAGGCAGCGACAAGCTAACGAAGCTTAAGTCAGTTCTCAAGAAGCTAAACTCATTCAACAACAAGCAAATCCGCCCTACGTCGAGCTCAGTAG   100
Gb1-GbSAUR86        ATGGCGAAAGGCAGCGACAAGCTAACGAAGCTTAAGTCAGTTCTCAAGAAGCTAAACTCATTCAACAACAAGCAAATCCGCCCTACGTCGAGCTCAGTAG   100
Gb2-GOBAR_DD18451   ATGGCGAAAGGCAGCGACAAGCTAACGAAGCTTAAGTCAGTTCTCAAGAAGCTAAACTCATTCAACAACAAGCAAATCCGCCCTACGTCGAGCTCAGTAG   100

Gh1-GhSAUR129       CGGCGTCGGCTTCGGATATCGACGAGGATTCATCGTCGGCAAATCTCCACCCCGTCTACGTTGGAAAATCTCGGAGGCGATACCTTATTAGCTCCGACAT   200
Gb1-GbSAUR86        CGGCGTCGGCTTCGGATATCGACGAGGATTCATCGTCGGCAAATCTCCACCCCGTCTACGTTGGAAAATCTCGGAGGCGATACCTTATTAGCTCCGACAT   200
Gb2-GOBAR_DD18451   CGGCGTCGGCTTCGGATATCGACGAGGATTCATCGTCGGCAAATCTCCACCCCGTCTACGTTGGAAAATCTCGGAGGCGATACCTTATTAGCTCCGACAT   200

Gh1-GhSAUR129       TATCAAAAGCCCTTTGTTCCGTGAACTCGCGGAATGGTCGCCGGGCGATAACGACGCCGTCATCAACGTGTCGTGCGAGGTTGTCTTGTTCGAACACTTG   300
Gb1-GbSAUR86        TATCAAAAGCCCTTTGTTCCGTGAACTCGCGGAATGGTCGCCGGGCGATAACGACGCCGTCATCAACGTGTCGTGCGAGGTTGTCTTGTTCGAACACTTG   300
Gb2-GOBAR_DD18451   TATCAAAAGCCCTTTGTTCCGTGAACTCGCGGAATGGTCGCCGGGCGATAACGACGCCGTCATCAACGTGTCGTGCGAGGTTGTCTTGTTCGAACACTTG   300

Gh1-GhSAUR129       CTTTGGATGCTCGAAAATGCTGATCCTCAGGCTGAGTCCTTGGAAGAACTAGTGGAGTTTTACGCATGTTG                                371
Gb1-GbSAUR86        CTTTGGATGCTCGAAAATGCTGATCCTCAGGCTGAGTCCTTGGAAGAACTAGTGGAGTTTTACGCATGTTG                                371
Gb2-GOBAR_DD18451   CTTTGGATGCTCGAAAATGCTGATCCTCAGGCTGAGTCCTTGGAAGAACTAGTGGAGTTTTACGCATGTTG                                371
```

GhSAUR130

```
Gh1-GhSAUR130       ATGGCGAAATTTGGGAAGCTAACGAAGCTCAAGTCTGCAATAAAGAGGTGGCCGTCTTTAACGAAGCTGACCCGTAGCAGCAGTGCCATAGCGGCTGCAG   100
Gr-GrSAUR95         ATGGCGAAATTTGGGAAGCTAACGAAGCTCAAGTCTGCAATAAAGAGGTGGCCGTCTTTAACGAAGCTGACCCGTAGCAGCAGTGCCATAGCGGCTGCAG   100
Gb2-GOBAR_DD29176   ATGGCGAAATTTGGGAAGCTAACGAAGCTCAAGTCTGCAATAAAGAGGTGGCCGTCTTTAACGAAGCTGACCCGTAGCAGCAGTGCCATAGCGGCTGCAG   100

Gh1-GhSAUR130       CAGAACCCGAAGGGAAGTCGGTTCCAAAGGGACTTCATGCAGTTTACGTCGGCAAGTCGCGGCGGAGGTACCTGGTAGGTTCCGAAATCATGTGCCACCC   200
Gr-GrSAUR95         CAGAACCCGAAGGGAAGTCGGTTCCAAAGGGACTTCATGCAGTTTACGTCGGCAAGTCGCGGCGGAGGTACCTGGTAGGTTCCGAAATCATGTGCCACCC   200
Gb2-GOBAR_DD29176   CAGAACCCGAAGGGAAGTCGGTTCCAAAGGGACTTCATGCAGTTTACGTCGGCAAGTCGCGGCGGAGGTACCTGGTAGGTTCCGAAATCATGTGCCACCC   200

Gh1-GhSAUR130       GTTGTTCCAGGAGCTGATTGATCGATCCTCTGGTGGTATGGATGATGATGGTGATGATGATCACGATCACTATCATTATGATGATGATGATAATGATGGT   300
Gr-GrSAUR95         GTTGTTCCAGGAGCTGATTGATCGATCCTCTGGTGGTATGGATGATGATGGTGATGATGATCACGATCACTATCATTATGACGATGATGATAATGATGGT   300
Gb2-GOBAR_DD29176   GTTGTTCCAGGAGCTGATTGATCGATCCTCTGGTGGTATGGATGATGATGGTGATGATGATCACGATCACTATCATTATGATGATGATGATAATGATGGT   300

Gh1-GhSAUR130       GGTGGCCATGAAGTTGTGGTTTCTTGTGAGGTTGTCTTGTTTGAACACTTGCTTTGGATGCTTGAGAACGATGGGGCTCAATTGGGGTCCATGGAAGAGC   400
Gr-GrSAUR95         GGTGGCCATGAAGTTGTGGTTTCTTGTGAGGTTGTCTTGTTTGAACACTTGCTTTGGATGCTTGAGAACGATGGGGCTCAATTGGGGTCCATGGAAGAGC   400
Gb2-GOBAR_DD29176   GGTGGCCATGAAGTTGTGGTTTCTTGTGAGGTTGTCTTGTTTGAACACTTGCTTTGGATGCTTGAGAACGATGGGGCTCAATTGGGGTCCATGGAAGAGC   400

Gh1-GhSAUR130       TGGTTGAGTTCTACACTTGCTA                                                                                 422
Gr-GrSAUR95         TGGTTGAGTTCTACACTTGCTA                                                                                 422
Gb2-GOBAR_DD29176   TGGTTGAGTTCTACACTTGCTA                                                                                 422
```

GhSAUR131

```
Gh1-GhSAUR131   ...................................................ATGGCTGGCTTTGGAAGAAGAACCATATCTATCAACCGCAAGCCAGCAA    49
Gr-GrSAUR96     ATGATAAGGTCGAAGAAACTGGAGGTGGCCCTCGCAAGGAAGTGGAAAACGATGGCTGGCTTTGGAAGAAGAACCATATCTATCAACCGCAAGCCAGCAA   100
Gb1-GbSAUR92    ...................................................ATGGCTGGCTTTGGAAGAAGAACCATATCTATCAACCGCAAGCCAGCAA    49

Gh1-GhSAUR131   GAGCAGGCCATTTTGTTGTCTACTCTTCGGACAAGAGACGTTTCGTTGTTCCCTTGGCATATCTCCGCACCAAGGTTTTCCAAGAGCTGTTAAGATTGTC   149
Gr-GrSAUR96     GAGCAGGCCATTTTGTTGTCTACTCTTCGGACAAGAGACGTTTCGTTGTTCCCTTGGCATATCTCCGCACCAAGGTTTTCCAAGAGCTGTTAAGATTGTC   200
Gb1-GbSAUR92    GAGCAGGCCATTTTGTTGTCTACTCTTCGGACAAGAGACGTTTCGTTGTTCCCTTGGCATATCTCCGCACCAAGGTTTTCCAAGAGCTGTTAAGATTGTC   149

Gh1-GhSAUR131   GGAGGAAGAGTTCGGAATGCCCAAGGATGGCCCTATAACTTTGCCATGCGATGCAGCAGTGTTGGAGTATGTCCTTTCTTTGCTCCGAAGTCACGTCTCT   249
Gr-GrSAUR96     GGAGGAAGAGTTCGGAATGCCCAAGGATGGCCCTATAACTTTGCCATGCGATGCAGCAGTGTTGGAGTATGTCCTTTCTTTGCTCCGAAGTCACGTCTCT   300
Gb1-GbSAUR92    GGAGGAAGAGTTCGGAATGCCCAAGGATGGCCCTATAACTTTGCCATGCGATGCAGCAGTGTTGGAGTATGTCCTTTCTTTGCTCCGAAGTCACGTCTCT   249

Gh1-GhSAUR131   TA                                                                                                     251
Gr-GrSAUR96     TA                                                                                                     302
Gb1-GbSAUR92    TA                                                                                                     251
```

GhSAUR132

```
Gh1-GhSAUR132   ATGGCTATCAGGAAATCAAACAAGTTGCCTCAAACAGCAGTCATCAAGCAAATCCTGAAAAGGTGCTCAAGCTTAGGAAAGAAACAGAGCTATGATGATG   100
Gr-GrSAUR97     ATGGCTATCAGGAAATCAAACAAGTTGCCTCAAACAGCAGTCATCAAGCAAATCCTGAAAAGGTGCTCAAGCTTAGGAAAGAAACAGAGCTATGATGATG   100
Gb1-GbSAUR94    ATGGCTATCAGGAAATCAAACAAGTTGCCTCAAACAGCAGTCATCAAGCAAATCCTGAAAAGGTGCTCAAGCTTAGGAAAGAAACAGAGCTATGATGATG   100

Gh1-GhSAUR132   AAGAAGGACTCCCTTTGGATGTCCCAAAAGGACATTTCGTTGTGTATGTTGGTGAAAACAGAAGCAGATACATTGTGCCCATTTCTTTCTTGAGCCGACC   200
Gr-GrSAUR97     AAGAAGGACTCCCTTTGGATGTCCCAAAAGGACATTTCGTTGTGTATGTTGGTGAAAACAGAAGCAGATACATTGTGCCCATTTCTTTCTTGAGCCGACC   200
Gb1-GbSAUR94    AAGAAGGACTCCCTTTGGATGTCCCAAAAGGACATTTCGTTGTGTATGTTGGTGAAAACAGAAGCAGATACATTGTGCCCATTTCTTTCTTGAGCCGACC   200

Gh1-GhSAUR132   GGAGTTCCAAAGCTTGCTTCATCAAGCTGAAGAAGAATTCGGTTTTGATCACGAGAGAGGACTCACCATTCCTTGTGAAGAAGTTGTTTTCCAGTCTCTA   300
Gr-GrSAUR97     GGAGTTCCAAAGCTTGCTTCATCAAGCTGAAGAAGAATTCGGTTTTGATCACGAGAGAGGACTCACCATTCCTTGTGAAGAAGTTGTTTTCCAGTCTCTA   300
Gb1-GbSAUR94    GGAGTTCCAAAGCTTGCTTCATCAAGCTGAAGAAGAATTCGATTTTGATCACGAGAGAGGACTCACCATTCCTTGTGAAGAAGTTGTTTTCCAGTCTCTA   300

Gh1-GhSAUR132   ACATCCATGCTCAGATG                                                                                      317
Gr-GrSAUR97     ACATCCATGCTCAGATG                                                                                      317
Gb1-GbSAUR94    ACATCCATGCTCAGATG                                                                                      317
```

GhSAUR133

```
Gh1-GhSAUR133      ATGTCGGCGGGGCTTGGAAAATGCGGTAAAATCCGCCACATTGTGAGGCTTCGCCAAATGTTGCGGCGGTGGAGGAACAACTCCCGCATGTCGGCCAGCC   100
Gr-GrSAUR99        ATGTCGGCGGGGCTTGGAAAATGCGGTAAAATCCGCCACATTGTGAGGCTTCGCCAAATGTTGCGGCGGTGGAGGAACAACTCCCGCATGTCGGCCAGCC   100
Gb2-GOBAR_AA12305  ATGTCGGCGGGGCTTGGAAAATGCGGTAAAATCCGCCACATTGTGAGGCTTCGCCAAATGTTGCGGCGGTGGAGGAACAAGTCCCGCATGTCGGCCAGTC   100

Gh1-GhSAUR133      GCATCCCATCCGATGTTCCGGCGGGGCACGTTGCGGTTTGCGTTGGGACGAGCTGCAGAAGATTCGTGGTGCGCGCCACGTACCTCAACCATCCCATCTT   200
Gr-GrSAUR99        GCATCCCATCCGATGTTCCGGCGGGGCACGTTGCGGTTTGCGTTGGGACGAGCTGCAGAAGATTCGTGGTGCGCGCCACGTACCTCAACCATCCCATCTT   200
Gb2-GOBAR_AA12305  GCATCCCATCCGATGTTCCGGCGGGGCACGTTGCAGTTTGCGTTGGGACGAGCTGCAGAAGATTCGTGGTGCGCGCCACGTACCTCAACCATCCCATTTT   200

Gh1-GhSAUR133      TAAGAAACTCCTTATCCAAGCCGAGGAAGAGTACGGCTTCACTAACCAAGGCCCATTGGCGATCCCCTGCGACGAGTCAGTTTTCGAAGAAGTGATCCGA   300
Gr-GrSAUR99        TAAGAAACTCCTTATCCAAGCCGAGGAAGAGTACGGCTTCACTAACCAAGGCCCATTGGCGATCCCCTGCGACGAGTCAGTTTTCGAAGAAGTGATCCGA   300
Gb2-GOBAR_AA12305  TAAGAGACTCCTTATCCAAGCCGAGGAAGAGTACGGCTTCACTAACCAAGGCCCATTGGCGATCCCCTGCGACGAGTCAGTTTTCGAAGAAGTGATCCGG   300

Gh1-GhSAUR133      TTTATTTCTCGCTCGGAGTCGGGTCACTTCGGTATGTTCGTGAACACCGAGGACTTTAAAGTGAAGTGTCACGTGGGAAGGAGTAAGCCTGATTTGTGGA   400
Gr-GrSAUR99        TTTATTTCTCGCTCGGAGTCGGGTCACTTCGGTATGTTCGTGAACACCGAGGACTTTAAAGTGAAGTGTCACGTGGGAAGGAGTAAGCCTGATTTGTGGA   400
Gb2-GOBAR_AA12305  TTTATTTCTCGCTCGGAGTCGGGTCACTCCGGTATGTTCGTGAACACCGAGGACTTTAAAGTGAAGTGTCACGTGGGAAGGAGTAAGCTTGATTTGTGGA   400

Gh1-GhSAUR133      CAGAATCGCGACCTTTACTTCATGGGTTTAAAGAAGTAGTGGGAAGAGAAAGACAACGGTTTTG                                       464
Gr-GrSAUR99        CAGAATCGCGACCTTTACTTCATGGGTTTAAAGAAGTAGTGGGAAGAGAAAGACAACGGTTTTG                                       464
Gb2-GOBAR_AA12305  CAGAATCGCGACCTTTACTTCATGGGTTTAAAGAAGTAGTGGGAAGAGAAAGACAACGGTTTTG                                       464
```

GhSAUR148

```
Gh1-GhSAUR148      ATGGGGATTGGAGGAGACAAGCAGCAGCACGTGAGCTTCCACCTTCATATGCCGAACCTTCATCTTCATGGGTTCCATCATCATGAGAACAAGGACCTCA   100
Gr-GrSAUR33        ATGGGGATTGGAGGAGAGAAGCAGCAGCACGTGAGCTTCCACCTTCATATGCCGCACCTTCATCTTCATGGGTTCCATCATCATGAGAACAAGGACCTCA   100
Gb2-GOBAR_DD01513  ATGGGGATTGGAGGAGACAAGCAGCAGCACGTGAGCTTCCACCTTCATATGCCGCACCTTCATCTTCACGGGTTCCACCATCATGAGAACAAGGACCTCA   100

Gh1-GhSAUR148      AAGATATCCCAAAGGGGTGCCTGGCGGTCCTGGTGGGTCAAGGCCAAGAGCAGCAAAGGTTTGTGATCCCGCTCATCTATATCACCCACCCCTTGTTCAT   200
Gr-GrSAUR33        AAGATATCCCAAAGGGGTGCCTGGCGGTCCTGGTGGGTCAAGGCCAAGAGCAGCAAAGGTTTGTGATCCCGCTCATCTATATCACCCACCCCTTGTTCAT   200
Gb2-GOBAR_DD01513  AAGATATCCCAAAGGGGTGCCTGGCGGTCCTGGTGGGTCAAGGCCAAGAGCAGCAAAGGTTTGTGATCCCGCTCATCTATATCACCCACCCCTTGTTCAT   200

Gh1-GhSAUR148      GCAGCTCTTGAAGGAAGCGGAGGAAGAGTATGGTTTCGATCATGAAGGCCCCATCACCATCCCTTGCCATGTCCAGGAGTTTCGCAATG.......TTCA   293
Gr-GrSAUR33        GCAGCTCTTGAAGGAAGCGGAGGAAGAGTATGGTTTCGATCATGAAGGCCCCATCACCATCCCTTGCCATGTCCAGGAGTTTCGCAATG.......TTCA   293
Gb2-GOBAR_DD01513  GCAGCTCTTGAAGGAAGCGGAGGAAGAGTATGGTTTCGATCATGAAGGCCCCATCACCATCCCTTGCCATGTCCAGGAGTTTCGCAATGATTATTCTTTA   300

Gh1-GhSAUR148      AGGGATGATCGATAAAGACCACCATCAC.....CACCACCACCAT...............CATGGCT.........GGAGGAATATAAAGTTGCAAGCGT   364
Gr-GrSAUR33        AGGGATGATCGATAAAGACCACCATCAC.....CACCACCACCAT...............CATGGCT.........GGAGGAATATAAAGTTGCAAGCGT   364
Gb2-GOBAR_DD01513  CACATAAATTCATTTGGTTTCTTTTTGTTCTTTCTCCATTTCCATGCTAATGTGAACGTATATGGTGTAAAATTGAGGAGGAATATAAAGTTGCAAGCGT   400

Gh1-GhSAUR148      ACAAGAGACGGTGGGAACTTGGAACCTTTGTGGGGTTATG                                                               404
Gr-GrSAUR33        ACAAGAGACGGTGGGAATTTGGAACCTTTGTG........                                                               396
Gb2-GOBAR_DD01513  ACAAGAGACGGTGGGAACTTGGAACCTTTGTGGGGTTATG                                                               440
```

GhSAUR149

```
Gh1-GhSAUR149      ATGAGTAGTGGAGAGAAAAGCTTGAGGAACTTCCATTTACACCTACCACACCTTCATCATCATCATCA......AGGGAAGAAACAAACGAGGGATGTGC    94
Gr-GrSAUR34        ATGAGTAGTGGAGAGAAAAGCTTGAGGAACTTCCATTTACACCTACCACACCTTCATCATCATCATCATCA...AGGGAAGAAACAAACGAGGGATGTGC    97
Gb2-GOBAR_DD26658  ATGAGTAGTGGAGAGAAAAGCTTGAGGAACTTCCATTTACACCTACCACACCTTCATCATCATCATCATCATCAAGGGAAGAAACAAACGAGGGATGTGC   100

Gh1-GhSAUR149      CAAAAGGGTATTTGGCAATCATGGTGGGATCACAAGGAGAGGAGCGACAGAGATTTGTGGTGCCTGTTATGTACTTCAATCACCCCTTGTTCATGCGACT   194
Gr-GrSAUR34        CAAAAGGGTATTTGGCAATCATGGTGGGATCACAAGGAGAGGAGCGACAGAGATTTGTGGTGCCTGTTATGTACTTCAATCACCCCTTGTTCATGCGACT   197
Gb2-GOBAR_DD26658  CAAAAGGGTATTTGGCAATCATGGTGGGATCACAAGGAGAGGAGCGACAGAGATTTGTGGTGCCTGTTATGTACTTCAATCACCCCTTGTTCATGCGACT   200

Gh1-GhSAUR149      GTTGAAAGAGGCGGAGGAAGAGTACGGGTTCGACCAGAAGGGAACCATCACAATCCCCTGTCATGTGGAAGAATTCAGGAATATACGGGGCTTGATTGAT   294
Gr-GrSAUR34        GTTGAAAGAGGCGGAGGAAGAGTACGGGTTCGACCAGAAGGGAACCATCACAATCCCCTGTCATGTGGAAGAATTCAGGAATATACGGGGCTTGATTGAT   297
Gb2-GOBAR_DD26658  GTTGAAAGAGGCGGAGGAAGAGTACGGGTTCGACCAGAAGGGAACCATCACAATCCCCTGTCATGTGGAAGAATTCAGGAATATACGGGGCTTGATTGAT   300

Gh1-GhSAUR149      AAGGAAAAGTCTCTTCATCATCATCATCACCATCATCATGTGGGTTGTTTTAGGGTTTGA.......................................   354
Gr-GrSAUR34        AAGGAAAAGTCTCTTCATCATCATCATCACCATCATCATGTGGGTTGTTTTAGGGTTTGA.......................................   357
Gb2-GOBAR_DD26658  AAGGAAAAGTCTCTTCATCATCATCATCACCATCATCATATGG.TTGACGAGGATGATGATAAAAAAGATGATGCGATAATGGTTACAGATGTTGCTTCC   399

Gh1-GhSAUR149      ............................                                                                           354
Gr-GrSAUR34        ............................                                                                           357
Gb2-GOBAR_DD26658  AATTGTAGTGACTTTGGGTTATTTTCTTA                                                                          428
```

GhSAUR150

```
Gh1-GhSAUR150      ATGAGAAAGATAAGAGGTTTCAAGATCAAAAAATGGTTGGTCCGGATCTCGAGGTGGTTCATTCGCAAAGCTCGGAACCCACGTGGGTACTATCGCCTAA   100
Gr-GrSAUR35        ATGAGAAAGATAAGAGGTTTCAAGATAAAAAAATGGTTGGTCCGGATCTTGAGGTGGTTCATTCGCAAAGCTCGGAATCCATGTGGGTACTATCGCCTGA   100
Gb2-GOBAR_DD15989  ................................ATGGTTGGTCCGGATCTCGAGGTGATTCATTCGCAAAGCTCGGAACCCACGTGGGTACTATCGCCTAA    68

Gh1-GhSAUR150      CACAATCAAAATCAGAAGCGTTTT.................GGGCTAAATCTATTTGGTCATCAAAGTCCGGGTTCAGTTATGTTCCTATTGGTCAAGGC   183
Gr-GrSAUR35        CACAATCAGAATCAGAAGCGTTTT.................GGGCTAAATCTATATGGTCATCAAAGTCCGGGTTCAGTTATGTTCCTATTGGTCAAGGC   183
Gb2-GOBAR_DD15989  CACAATCAAAATCAGAAGCGTTTTTCAACACTTGATAAAAGGGGCTAAATCTATTTGGTCATCAAAGTCCGGGTTCAGTTATGTTCCTATTGGTCAAGGC   168

Gh1-GhSAUR150      CCAATCAACGAGAAGCTTACGACAGTTCTGAAAGGACACTTGGTCGTCTATGTCGGTCAAAAAGACAACGATTACCAGAGAGTTTTGGTGTCAATTATTT   283
Gr-GrSAUR35        CCAATCAACGAGAAGCTTACGAAAGTTCTAAAAGGACACTTGGTCGTCTATGTCGGTCAAAAAGACAACGATTACCATAGAGTTTTGGTGTCAATTATTT   283
Gb2-GOBAR_DD15989  CCAATCAACGAGAAGCTTACGACAGTTCTGAAAGGACACTTGGTCGTCTATGTCGGTCAAAAAGACAACGATTACCATAGAGTTTTGGTGTCAATTATTT   268

Gh1-GhSAUR150      ATTTTAACCACCCTTTGTCCGACGAGCTATTAAGGGAAGCAGAAGAGGAGTATGGGTTTAGCCACCAAGGTGGTATCACCATTTCCTGCCTGTTCTTATA   383
Gr-GrSAUR35        ATTTTAACCACCCTTTGTCCGACGAGCTATTAAGGGAAGCAGAAGAGGAGTATGGGTTTAGCCACCAAGGTGGTATCACCATTTCCTGCCTGTTCTTAGA   383
Gb2-GOBAR_DD15989  ATTTTAACCACCCTTTGTCCGACGAGCTATTAAGGGAAGCAGAAGAGGAGTATGGGTTTAGCCACCAAGGTG.TTCAAGAGGGTCTAGACCTATATCGTG   367

Gh1-GhSAUR150      G....................................................................................   384
Gr-GrSAUR35        GTTCAAGAGGGTCTAG.....................................................................   399
Gb2-GOBAR_DD15989  GTTGGAATCGGTGGAAGGAAAATGGTATGGAAGCACCACCACCATTGAAAAAGGCTGAGCCTTGGCTGGTGCTAGTGTTATTATG   452
```

GhSAUR151

```
Gh1-GhSAUR151      ATGAGAAAGATAAGAGGTTTCAAGATCGGAAAACGGTTGGTCCGGATCTCGAGGTGGTTCATTCGCAAAGCTCGGCACCCACGTGGGTACTGTCGCCTGA   100
Gr-GrSAUR36        ATGAGAAAGATAAGAGGTTTCAAGATCGGAAAACGGTTGGTCCGGATCTCGAGGTGGTTCATTCGCAAAGCTCGGCACCCACGTGGGTACTGTCGCCTGA   100
Gb1-GbSAUR104      ATGAGAAAGATAAGAGGTTTCAAGATCGGAAAACGGTTGGTCCGGATCTCGAGGTGGTTCATTCGCAAAGCTCGGCACCCACGTGGGTACTGTCGCCTGA   100
Gb2-GOBAR_DD15990  ATGAGAAAGATAAGAGGTTTCAAGATCGGAAAACGGTTGGTCCGGATCTCGAGGTGGTTCATTCGCAAAGCTCGGCACCCACGTGGGTACTGTCGCCTGA   100

Gh1-GhSAUR151      CACAATCAGAATCAAAATCGTTTTGTAAGTCCAAGTCTTTCTCGAAGCTCATTAGCTGGGGTCAATGCTTGACAAAAAGGGCTAAATCTATATGCTCATC   200
Gr-GrSAUR36        CACAATCAGAATCAAAATCGTTTTGTAAGTCCAAGTCTTTCTCGAAGCTCATTAGCTGGGGTCAATGCTTGACAAAAAGGGCTAAATCTATATGCTCATC   200
Gb1-GbSAUR104      CACAATCAGAATCAAAATCGTTTTGTAAGTCCAAGTCTTTCTCGAAGCTCATTAGCTGGGGTCAATGCTTGACAAAAAGGGCTAAATCTATATGCTCATC   200
Gb2-GOBAR_DD15990  CACAATCAGAATCAAAATCGTTTTGTAAGTCCAAGTCTTTCTCGAAGCTCATTAGCTGGGGTCAATGCTTGACAAAAAGGGCTAAATCTATATGCTCATC   200

Gh1-GhSAUR151      AAAGCCCGGGTTGGGTTACGTTCCTATTGGTCAAGACCCAATCAACGAGAAGCTTACGGAAGTTCCGAAAGGACACTTGGCCATCTATATCGGCCAAAAA   300
Gr-GrSAUR36        AAAGCCCGGGTTGGGTTACGTTCCTATTGGTCAAGACCCAATCAACGAGAAGCTTACGGAAGTTCCGAAAGGACACTTGGCCATCTATATCGGCCAAAAA   300
Gb1-GbSAUR104      AAAGCCCGGGTTGGGTTACGTTCCTATTGGTCAAGACCCAATCAACGAGAAGCTTACGGAAGTTCCGAAAGGACACTTGGCCATCTATATCGGCCAAAAA   300
Gb2-GOBAR_DD15990  AAAGCCCGGGTTGGGTTACGTTCCTATTGGTCAAGACCCAATCAACGAGAAGCTTACGGAAGTTCCGAAAGGACACTTGGCCATCTATATCGGCCAAAAA   300

Gh1-GhSAUR151      GACGGCGATTACCATAGAGTTTTGGTGCCGGTTATTTATTTTAACCACCCTTTGTTTGGCGAGCTATTAAGGGAAGCCGAAGAGGAGTATGGGTTTAGCC   400
Gr-GrSAUR36        GACGGCGATTACCATAGAGTTTTGGTGCCGGTTATTTATTTTAACCACCCTTTGTTTGGCGAGCTGTTAAGGGAAGCCGAAGAGGAGTATGGGTTTAGCC   400
Gb1-GbSAUR104      GACGGCGATTACCATAGAGTTTTGGTGCCGGTTATTTATTTTAACCACCCTTTGTTTGGCGAGCTATTAAGGGAAGCCGAAGAGGAGTATGGGTTTAGCC   400
Gb2-GOBAR_DD15990  GACGGCGATTACCATAGAGTTTTGGTGCCGGTTATTTATTTTAACCACCCTTTGTTTGGCGAGCTATTAAGGGAAGCCGAAGAGGAGTATGGGTTTAGCC   400

Gh1-GhSAUR151      ACCAAGGAGGTATCACCATTCCCTGTCGATTCTCGGAGTTCGAGAGGGTCCAGACTCGTATCGCAGCTGGAACCGGTGGAAGGAAAATGGTATGGAAGCG   500
Gr-GrSAUR36        ACCAAGGAGGTATCACCATTCCCTGTCGATTCTCGGAGTTCGAGAGGGTCCAGACTCGTATCGCAGCTGGAACCGGTGGAAGGAAAATGGTATGGAAGCG   500
Gb1-GbSAUR104      ACCAAGGAGGTATCACCATTCCCTGTCGATTCTCGGAGTTCGAGAGGGTCCAGACTCGTATCGCAGCTGGAACCGGTGGAAGGAAAATGGTATGGAAGCG   500
Gb2-GOBAR_DD15990  ACCAAGGAGGTATCACCATTCCCTGTCGATTCTCGGAGTTCGAGAGGGTCCAGACTCGTATCGCAGCTGGAACCGGTGGAAGGAAAATGGTATGGAAGCG   500

Gh1-GhSAUR151      CCACCACCATTG   512
Gr-GrSAUR36        CCACCACCATTG   512
Gb1-GbSAUR104      CCACCACCATTG   512
Gb2-GOBAR_DD15990  CCACCACCATTG   512
```

GhSAUR152

```
Gh1-GhSAUR152   ATGAAGAAAATTAGAGGGTTCAAGCTTGGACGCAAGCTAGTGAAGGTATTCAAATGGGTAAGCCGACCCGGAAGAAAAAACTGCATGAATAGTTTGTTGA   100
Gr-GrSAUR37     ATGAAGAAAATTAGAGGGTTCAAGCTTGGACGCAAGCTAGTGAAGGTATTCAAATGGGTAAGCCGTCCCGGAAGAAAAAACTGCATGAATAGTTTGTTGA   100
Gb1-GbSAUR105   ATGAAGAAAATTAGAGGGTTCAAGCTTGGACGCAAGCTAGTGAAGGTATTCAAATGGGTAAGCCGACCCGGAAGAAAAAACTGCATGAATAGTTTGTTGA   100

Gh1-GhSAUR152   GACCTCCGACACCAAGCTACAACCCGTTATCCAGAATCTGGTCTTTTGCGAGGTTTCTTCGAGGTGGAAGCAAGGAACTGAGTAGTTGGAAGCTGGATCC   200
Gr-GrSAUR37     GACCTCCGACACCAAGCTACAACCCGTTATCCAGAATCTGGTCTTTTGCGAGGTTTCTTCGAGGTGGAAGGAAGGAACTGAGTAGTTGGAAGCTGGATCC   200
Gb1-GbSAUR105   GACCTCCGACACCAAGCTACAACCCGTTATCCAGAATCTGGTCTTTTGCGAGGTTTCTTCGAGGTGGAAGCAAGGAACTGAGTAGTTGGAAGCTGGATCC   200

Gh1-GhSAUR152   AGGTTATATCCAATTGGGTGAGAAGGGAGTGAAGCGGGTTGAGGTACCCAAGGGACATCTCGCAGTGTACGTTGGCGAATCAGAAGGTGAGACGAGGAGG   300
Gr-GrSAUR37     AGGTTATATCCAATTGGGTGAGAAGGGAGTGAAGCGGGTTGAGGTACCCAAGGGACATCTCGCAGTGTACGTTGGCGAATCAGAAGGTGAGACGAGGAGG   300
Gb1-GbSAUR105   AGGTTATATCCATTTGGGTGAGAAGGGAGTGAAGCGGGTTGAGGTACCCAAGGGACATCTCGCAGTGTACGTTGGCGAATCAGAAGGTGAGACGAGGAGG   300

Gh1-GhSAUR152   GTGGTAGTGCCTGTGATTCATTTCAATCACCCGCTATTCGGGGAGCTGTTGGAGGAAGCGGAGCGGGTTTACGGGTTCAATCAATCCGGCCGGATCACTT   400
Gr-GrSAUR37     GTGGTAGTGCCTGTGATTTATTTCAATCACCCGCTATTCGGGGAGCTGTTGGAGGAAGCGGAGCGGGTTTACGGGTTCAATCAATCCGGCCGGATCACTT   400
Gb1-GbSAUR105   GTGGTAGTGCCTGTGATTTATTTCAATCACCCGCTATTCGGGGAGCTGTTGGAGGAAGCGGAGCGGGTTTACGGGTTCAATCAATCCGGCCGGATCACTT   400

Gh1-GhSAUR152   TGCCTTGCGGGATTTCAGAGTTCGAGAAGGTTAAGATGAGGATTGCCGATTGGGATCATTGCCGACGGACACAACATCGTTGCTATTTGTA   491
Gr-GrSAUR37     TGCCTTGCGGGATTTCAGAGTTCGAGAAGGTTAAGATGAGAATTGCCGATTGGGATCATTGCCGACGGACACAACATCGTTGCTATTTGTA   491
Gb1-GbSAUR105   TGCCTTGCGGGATTTCAGAGTTCGAGAAGGTTAAGATGAGAATTGCCGATTGGGATCATTGCCGACGGACACAACATCGTTGCTATTTGTA   491
```

GhSAUR171

```
Gh1-GhSAUR171      ATGAGAAAGATAAGAGGATTCAAGATCGGAAAACGGGTTATCCGATTTTCAAAGTGGGTCATCGGCAAAGCTCGGAGGAAACCATATGGATACCGTCGTT   100
Gr-GrSAUR86        ATGAGAAAGATAAGAGGATTCAAGATCGGAAAACGGGTTATCCGATTTTCAAAGTGGGTCATCGGCAAAGCTCGGAGGAAACCATATGGATACCGTCGTT   100
Gb2-GOBAR_DD02815  ATGAGAAAGATAAGAGGATTCAAGATCGGAAAACGGGTTATCCGATTTTCAAAGTGGGTCATCGGCAAAGCTCGGAGGAAACCATATGGATACCGTCGTT   100

Gh1-GhSAUR171      TAAAACAGGGAGGACTGTTTAGCAAATCAAACTCTTTGTCCAAGTTCATCAACTGGGGTCGCCGGTTGAAAAACGCGGCTAAATCTATATGTTCGGTGAA   200
Gr-GrSAUR86        TAAAACAGGGAGGACTGTTTAGCAAATCAAACTCTTTGTCCAAGTTCATCAACTGGGGTCGCCGGTTGAAAAACGGGGCTAAATCTATATGTTCGGTGAA   200
Gb2-GOBAR_DD02815  TAAAACAGGGAGGACTGTTTAGCAAATCAAACTCTTTGTCCAAGTTCATCAACTGGGGTCGCCGGTTGAAAAACGCGGCTAAATCTATATGTTCGGTGAA   200

Gh1-GhSAUR171      ACTCGGGTCTGGTTATGGACCCATAGAGGAAAAACCGATTGAAGTTCCGAAAGGTCATTTGGCCGTCTACGTCGGTCGAAGAAACGGCGGCGATTTTCAT   300
Gr-GrSAUR86        ACTCGGGTCGGCTTATGGACCCATAGAGGAAAAACCGATTGAAGTTCCGAAAGGTCATTTGGCCGTCTACGTCGGTCGAAGAAACGGCGGCGATTTTCAT   300
Gb2-GOBAR_DD02815  ACTCGGGTCTGGTTATGGACCCATAGAGGAAAAACCGATTGAAGTTCCGAAAGGTCATTTGGCCGTCTACGTCGGTCGAAGAAACGGCGGCGATTTTCAT   300

Gh1-GhSAUR171      AGAGTGTTGGTGCCGGTTATTTATTTTAATCACCCTTTGTTCGGCGAGCTATTAAGAGAAGCTGAAAAGGAGTATGGGTTTTGTCACCAAGGTGGAATCA   400
Gr-GrSAUR86        AGAGTGTTGGTGCCGGTTATTTATTTTAATCACCCTTTGTTCGGCGAGCTATTAAGAGAAGCTGAAAAGGAGTATGGGTTTTGTCACCAAGGTGGAATCA   400
Gb2-GOBAR_DD02815  AGAGTGTTGGTGCCGGTTATTTATTTTAATCACCCTTTGTTCGGCGAGCTATTAAGAGAAGTTGAAAAGGAGTATGGGTTTTGTCACCAAGGTGGAATCA   400

Gh1-GhSAUR171      CGATTCCCTGTGGGTTCTCGGAATTTGAGAAGGTTCAGACCCGGATCGCCGCCGGAACCGTCGGAAGGAAAGCCGTTTGGAAGCGTCACTATTG   494
Gr-GrSAUR86        CGATTCCCTGTGGGTTCTCGGAATTTGAGAAGGTTCAGACCCGGATCGCCGCCGGAACCGTCGGAAGGAAAGCCGTTTGGAAGCGTCACTATTG   494
Gb2-GOBAR_DD02815  CGATTCCCTGTGGGTTCTCGGAATTTGAGAAGGTTCAGACCCGGATCGCCGCCGGAACCGTCGGAAGGAAAGCCGTTTGGAAGCGTCACTATTG   494
```

GhSAUR172

```
Gh1-GhSAUR172   ATGGGTAATGGTGAGAAAAATTCAAGGAACTTTCATTTACACATACCCCACCTTCATCATCATCATCATCAAGGGAAGAAGCAAGCAAAGGGTGTGCCAA   100
Ga-GaSAUR50     ATGGGTAATGGTGAGAAAAATTCAAGGAACTTTCATTTACACATACCCCACCTTCATCATCATCATCA...AGGGAAGAAGCAAGCAAAGGGTGTGCCAA    97
Gb1-GbSAUR54    ATGGGTAATGGTGAGAAAAATTCAAGGAACTTTCATTTACACATACCCCACCTTCATCATCATCATCATCAAGGGAAGAAGCAAGCAAAGGGTGTGCCAA   100

Gh1-GhSAUR172   AAGGGTGTTTGGCTATCAAAGTAGGACCAAAAGAAGAAGAACAACAAAGGTTTGTGGTGCCGGTTTTGTATTTTAATCATCCTTTGTTCATGCAATTGTT   200
Ga-GaSAUR50     AAGGGTGTTTGGCTATCAAAGTAGGACCAAAAGAAGAAGAACAACAAAGGTTTGTGGTGCCGGTTTTGTATTTTAATCATCCTTTGTTCATGCAATTGTT   197
Gb1-GbSAUR54    AAGGGTGTTTGGCTATCAAAGTAGGACCAAAAGAAGAAGAACAACAAAGGTTTGTGGTGCCGGTTTTGTATTTTAATCATCCTTTGTTCATGCAATTGTT   200

Gh1-GhSAUR172   GAAAGAAGCTGAGGAAGAGTATGGGTTTGAGCAGAAGGGTATGATTACCATCCCTTGTCATGTAGAAGAGTTTAGGAATGTTCGTGGGCTGATTGATGGT   300
Ga-GaSAUR50     GAAAGAAGCTGAGGAAGAGTATGGGTTTGAGCAGAAGGGTATGATTACCATCCCTTGTCATGTAGAAGAGTTTAGGAATGTTCGTGGGTTGATTGATGGT   297
Gb1-GbSAUR54    GAAAGAAGCTGAGGAAGAGTATGGGTTTGAGCAGAAGGGTATGATTACCATCCCTTGTCATGTAGAAGAGTTTAGGAATGTTCGTGGGCTGATTGATG..   298

Gh1-GhSAUR172   GAAAAGTCACTTCATCATCATCATCATCACCA.TCA.........CCAGCATG.TTGGCTGTTTTAGGGTTTGA.........................   363
Ga-GaSAUR50     GAAAAGTCACTTCATCATCATCATCATCATCA.TCATCATCATCACCATCATG.TTGGGTGTTTTAGGGTTTGA.........................   369
Gb1-GbSAUR54    ..TAATTCAGTTCATGGTCCCATATATATTTGGTTGGTGGATAAATGGGGATGATTATAGAAAAGAAAATATGATGACAAAGGTTGATCATAATTGTAGT   396

Gh1-GhSAUR172   ..............   363
Ga-GaSAUR50     ..............   369
Gb1-GbSAUR54    GCTTTAATTTGTTA   410
```

GhSAUR173

```
Gh1-GhSAUR173      ATGGGGAAAGGCAAAAAGGGCAACCTGATAATCAAGACTTGGGAGCGATGCAAATCCATTGGCCGTGGCCGCTCAAGACACGCCCCAGCTATTTATAAAA   100
Gr-GrSAUR88        ATGGGGAAAGGCAAAAAGGGCAACCTGATAATCAAGACTTGGGAGCGATGCAAATCCATTGGCCGTGGCCGCTCAAGACACGCCCCAGCTATTTATAAAA   100
Gb1-GbSAUR123      ATGGGGAAAGGCAAAAAGGGCAACCTGATAATCAAGACTTGGGAGCGATGCAAATCCATTGGCCGTGGCCGCTCAAGACACGCCCCAGCTATTTATAAAA   100
Gb2-GOBAR_DD04584  ATGGGGAAAGGCAAAAAGGGCAACCTGATAATCAAGACTTGGGAGCGATGCAAATCCATTGGCCGTGGCCGCTCAAGACACGCCCCAGCTATTTATAAAA   100

Gh1-GhSAUR173      AGAGCAAATCATGGCCATCAATTGATGTTTCTCTGGAAGAAGAGAAACGCACAAGGAAGAATCGAGTGGCTCCGGAAGGTTGCTTCACGGTGTATGTTGG   200
Gr-GrSAUR88        AGAGCAAATCATGGCCATCAATTGATGTTTCTCTGGAAGAAGAGAAACGCACAAGGAAGAATCGAGTGGCTCCGGAAGGTTGCTTCACGGTGTATGTTGG   200
Gb1-GbSAUR123      AGAGCAAATCATGGCCATCAATTGATGTTTCTCTGGAAGAAGAGAAACGCACAAGGAAGAATCGAGTGGCTCCGGAAGGTTGCTTCACGGTGTATGTTGG   200
Gb2-GOBAR_DD04584  AGAGCAAATCATGGCCATCAATTGATGTTTCTCTGGAAGAAGAGAAACGCACAAGGAAGAATCGAGTGGCTCCGGAAGGTTGCTTCACGGTGTATGTTGG   200

Gh1-GhSAUR173      ACCCCAGAAACAAAGGTTCGTGATCAAAACCGAATACGCAAATCATCCGCTTTTCAAGATTCTACTCGAGGAAGCCGAGTCTGAATACGGATTCACCAGC   300
Gr-GrSAUR88        ACCCCAGAAACAAAGGTTCGTGATCAAAACCGAATACGCAAATCATCCGCTTTTCAAGATTCTACTCGAGGAAGCCGAGTCTGAATACGGATTCACCAGC   300
Gb1-GbSAUR123      ACCCCAGAAACAAAGGTTCGTGATCAAAACCGAATACGCAAATCATCCGCTTTTCAAGATTCTACTCGAGGAAGCCGAGTCTGAATACGGATTCACCAGC   300
Gb2-GOBAR_DD04584  ACCCCAGAAACAAAGGTTCGTGATCAAAACCGAATACGCAAATCATCCGCTTTTCAAGATTCTACTCGAGGAAGCCGAGTCTGAATACGGATTCACCAGC   300

Gh1-GhSAUR173      GAAGGACCCCTTATGCTCCCCTGCAACGTTGATCTCTTTTGCAAGGTGTTACTGGCCATGGACGATGGCGACAATACCATCCGCCAAGGATGCGGTTTCG   400
Gr-GrSAUR88        GAAGGACCCCTTATGCTCCCCTGCAACGTTGATCTCTTTTGCAAGGTGTTACTGGCCATGGACGATGGCGACAATACCATCCGCCAAGGATGCGGTTTCG   400
Gb1-GbSAUR123      GAAGGACCCCTTATGCTCCCCTGCAACGTTGATCTCTTTTGCAAGGTGTTACTGGCCATGGACGATGGCGACAATACCATCCGCCAAGGATGCGGTTTCG   400
Gb2-GOBAR_DD04584  GAAGGACCCCTTATGCTCCCCTGCAACGTTGATCTCTTTTGCAAGGTGTTACTGGCCATGGACGATGGCGACAATACCATCCGCCAAGGATGCGGTTTCG   400

Gh1-GhSAUR173      CCAATGGTTATGGTTCCTATCGCCTCCTCACCCCACCTTGGATGATGGCGACAAATCAGATCTA   464
Gr-GrSAUR88        CCAATGGTTATGGTTCCTATCGCCTCCTCACCCCACCTTGGATGATGGCGACAAATCAGATCTA   464
Gb1-GbSAUR123      CCAATGGTTATGGTTCCTATCGCCTCCTCACCCCACCTTGGATGATGGCGACAAATCAGATCTA   464
Gb2-GOBAR_DD04584  CCAATGGTTATGGTTCCTATCGCCTCCTCACCCCACCTTGGATGATGGCGACAAATCAGATCTA   464
```

GhSAUR174

```
Gh1-GhSAUR174      ATGAAGAAAATTAGAGGGTTCAAGCTTGGGCGCAAGCTAGTGAAGGTGTTCAAATGGATAATCCCACCTAGAAGAAGAAACTACCGGAATGGTTTCTTGA   100
Gr-GrSAUR91        ATGAAGAAAATTAGAGGGTTCAAGCTTGGGCGCAAGCTAGTGAAGGTGTTCAAATGGATAATCCCACCTAGAAGAAGAAACTACCGGAATGGTTTCTTGA   100
Gb1-GbSAUR137      ATGAAGAAAATTAGAGGGTTCAAGCTTGGGCGCAAGCTAGTGAAGGTGTTCAAATGGATAATCCCACCTAGAAGAAGAAACTACCGGAATGGTTTCTTGA   100
Gb2-GOBAR_DD20646  ATGAAGAAAATTAGAGGGTTCAAGCTTGGGCGCAAGCTAGTGAAGGTGTTCAAATGGATAATCCCACCTAGAAGAAGAAACTACCGGAATGGTTTCTTGA   100

Gh1-GhSAUR174      GGCATCCGACTCGAAGCTACAACCCTTTATCAAGACTCTGCTCTTTCGCGACGTTTCTTCGACAGGGGACCAAAAGGCTGTGTAATTCGGACTCGGATCC   200
Gr-GrSAUR91        GGCATCCGACTCGAAGCTACAACCCTTTATCAAGACTCTGCTCTTTCGCGACGTTTCTTCGACAGGGAACCAGAAGGCTGTGTAATTCGGACTCGGATCC   200
Gb1-GbSAUR137      GGCATCCGACTCGAAGCTACAACCCTTTATCAAGACTCTGCTCTTTCGCGACGTTTCTTCGACAGGGAACCAAAAGGCTGTGTAATTCGGACTCGGATCC   200
Gb2-GOBAR_DD20646  GGCATCCGACTCGAAGCTACAACCCTTTATCAAGACTCTGCTCTTTCGCGACGTTTCTTCGACAGGGAACCAAAAGGCTGTGTAATTCGGACTCGGATCC   200

Gh1-GhSAUR174      GGGTTACATTCAATTGGGTGAAAAGGGAGTGAAGCGGGTAGGAGTGCCGAAGGGGCACCTTGCAGTGTCCGTGGGGGAATCAGACGGTAACATGAGGAGG   300
Gr-GrSAUR91        GGGTTACATTCAATTGGGTGAAAAGGGAGTGAAGCGGGTAGGAGTGCCGAAGGGGCACCTTGCAGTGTACGTGGGGGAATCAGACGGTAACATGAGGAGG   300
Gb1-GbSAUR137      GGGTTACATTCAATTGGGTGAAAAGGGAGTGAAGCGGGTAGGAGTGCCGAAGGGGCACCTTGCAGTGTCCGTGGGGGAATCAGACGGTAACATGAGGAGG   300
Gb2-GOBAR_DD20646  GGGTTACATTCAATTGGGTGAAAAGGGAGTGAAGCGGGTAGGAGTGCCGAAGGGGCACCTTGCAGTGTCCGTGGGGGAATCAGACGGTAACATGAGGAGG   300

Gh1-GhSAUR174      GTGGTAGTGCCTGTGATTTATTTCAATCACCCGCTATTTGGGGAGCTGTTGAAGGAAGCGGAGATGGTTTACGGGTTCAATCAATCGGGTGGGATCACGT   400
Gr-GrSAUR91        GTGGTAGTGCCTGTGATTTATTTCAATCACCCGCTATTTGGGGAGCTGTTGAAGGAAGCGGAGCTGGTTTACGGGTTCAATCAATCGGGTGGGATCACGT   400
Gb1-GbSAUR137      GTGGTAGTGCCTGTGATTTATTTCAATCACCCGCTATTTGGGGAGCTGTTGAAGGAAGCGGAGCTGGTTTACGGGTTCAATCAATCGGGTGGGATCACGT   400
Gb2-GOBAR_DD20646  GTGGTAGTGCCTGTGATTTATTTCAATCACCCGCTATTTGGGGAGCTGTTGAAGGAAGCGGAGCTGGTTTACGGGTTCAATCAATCGGGTGGGATCACGT   400

Gh1-GhSAUR174      TACCATGTGGGATTTCGGAGTTCGAGAAGGTTAAGATGAGAATTGCCGATTGGGATCATTGTCGACGGAAACAACATCGTCGTTATTTTTATTG   494
Gr-GrSAUR91        TACCATGTGGGATTTCGGAGTTCGAGAAGGTTAAGATGAGAATTGCCGATTGGGATCATTGTCGACGGAAACAACATCGTCGTTATTTTTATTG   494
Gb1-GbSAUR137      TACCATGTGGGATTTCGGAGTTCGAGAAGGTTAAGATGAGAATTGCCGATTGGGATCATTGTCGACGGAAACAACATCGTCGTTATTTTTATTG   494
Gb2-GOBAR_DD20646  TACCATGTGGGATTTCGGAGTTCGAGAAGGTTAAGATGAGAATTGCCGATTGGGATCATTGTCGACGGAAACAACATCGTCGTTATTTTTATTG   494
```
